# Synthesis of the Tetracyclic Spiro-naphthoquinone Chartspiroton

**DOI:** 10.1021/acs.orglett.4c00695

**Published:** 2024-04-01

**Authors:** Liesa Röder, Klaus Wurst, Thomas Magauer

**Affiliations:** Department of Organic Chemistry and Center for Molecular Biosciences, University of Innsbruck, 6020 Innsbruck, Austria; Department of General Inorganic and Theoretical Chemistry, University of Innsbruck, 6020 Innsbruck, Austria; Department of Organic Chemistry and Center for Molecular Biosciences, University of Innsbruck, 6020 Innsbruck, Austria

## Abstract

Chartspiroton is a recently discovered naphthoquinone natural product that features a spiro-fused benzofuran lactone. We report its first synthesis via an 11-step linear sequence. The sterically hindered tetra-*ortho*-substituted biaryl subunit was installed by base-induced ring expansion of a readily available 1,3-indandione. This step also liberated the fully substituted naphthalene core unit at the same time. The unique spiro-fused benzofuran lactone of the natural product was constructed via late-stage oxidation of an advanced naphthoquinone.

## Graphical abstract

**Figure:**
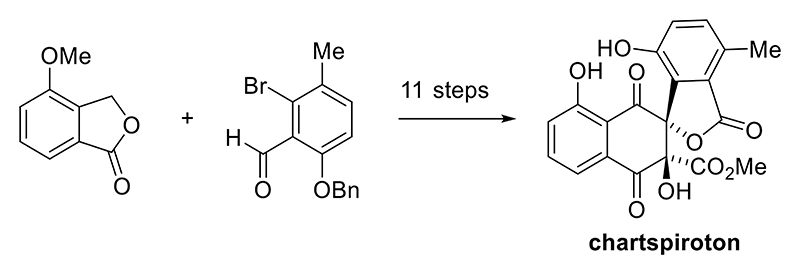

In 2020, Hu and co-workers reported the isolation of chartspiroton (**1**) from the endophytic *Streptomyces* sp. SH-1.2-R-1 in *Dendrobium officinale* (Figure 1A).^1^ *Dendrobium officinale* is well known in traditional Chinese medicine and has demonstrated various clinical benefits, such as hepatoprotective, anticancer, hypoglycemic, antifatigue, and gastric ulcer protection.^2,3^ From a structural perspective, chartspiroton displays a unique 6/6/5/6 tetracyclic polyketide scaffold with a spiro-fused benzofuran lactone moiety^1^ and showcases structural elements that biosynthetically relate with elsamicin B (**2**) and its aglycon chartarin (**3**). Together with the gilvocarcins, as exemplified by defucogilvocarcin M (**4**), this natural product family stands out for its potent antibacterial and antitumor properties.^4,5^ It is no surprise that the structural features and promising biological activities have sparked great interest in the synthetic community.^6−15^ As part of our continuous effort to develop practical and scalable methods to synthesize polysubstituted, highly functionalized (hetero)-arenes, we previously set out to investigate a variety of ring expansion reactions.^16,17^ While this ultimately enabled access to the polyketide natural products chartarin (**3**)^16^ and defucogilvocarcin M (**4**),^17^ we considered those strategies to be unsuited for reaching the more complex structure of chartspiroton (**1**).

As a result, we evaluated alternative ring expansion strategies for their potential to produce the desired molecular architecture. This guided us to 2-arylated 1,3-indandiones as valuable precursors to tetra-*ortho*-substituted biaryl subunits.^18,19^ In seminal work by Radulescu and Gheorgiu, it was shown that indanones undergo acid- and base-mediated ring expansion to tetrasubstituted naphthalenes.^18^ More recently, Zhang and co-workers extended this chemistry to the construction of 1,4-naphthoquinones via a copper(I)-mediated insertion of alkenes into 2-aryl-1,3-indandiones.^19^ The implementation of this ring expansion strategy to the retrosynthesis of chartspiroton (**1**) led to removal of the spiro-lactone and revealed biaryl **5a** as our first key intermediate (Figure 1B).

Ring contraction of the naphthalene component produced the 2,2-disubstituted 1,3-indandione **6**, which was further traced back to **7**. The 2-aryl substituent of **7** was envisioned to be derived from the base-mediated condensation of methoxyisobenzofuranone **8** and aldehyde **9**.

We initiated our synthetic studies toward chartspiroton (**1**) by preparing known methoxyisobenzofuranone **8** and *o,o*-disubstituted aldehyde **9** (Scheme 1). The former was accessible through a two-step sequence starting from inexpensive 3-hydroxybenzoic acid.^20,21^ The aldehyde **9** was synthesized in four steps from 2-bromo-4-fluorotoluene involving formylation^22^ and benzyl protection of the corresponding phenol (for details, see the Supporting Information). Having acquired both components, the aldehyde **9** underwent basic condensation with **8** to furnish the corresponding 1,3-indandione **7** in 58% yield.^23^ As reported by Freedman and co-workers, the use of ethyl propionate as solvent effectively removed the generated water and led to improved yields.^24^ While both *ortho*-substituents of aldehyde **9** were tolerated in this reaction, we encountered difficulties when attempting the C2-functionalization of the 1,3-indandione. We concluded that steric encumbrance arising from the Br- and OBn-substituent prevented any further *C*-alkylation. Instead, we observed exclusive formation of the corresponding *O*-alkylated intermediates **10a** and **10b** in 90% overall yield when reacting **7** with allyl bromide under basic conditions (K_2_CO_3_). The inconsequential mixture of regioisomers **10a** and **10b** were separated by flash column chromatography to allow for structure validation of **10b** via single-crystal X-ray analysis. Both regioisomers underwent the subsequent [3,3]-Claisen rearrangement to give the *C*-alkylated 1,3-indandione **11** in 76% yield. Ozonolysis of the terminal alkene followed by reductive workup employing dimethyl sulfide gave the corresponding aldehyde **12**. Next, **12** was subjected to a two-step sequence involving Pinnick−Lindgren oxidation^25,26^ and methylation to give the corresponding methyl ester **6**.

With methyl ester **14** in hand, we found a suitable precursor to investigate the key biaryl formation through the envisioned ring expansion reaction. Gratifyingly, treatment of **6** with potassium hexamethyldisilazide (KHMDS) at −78 °C induced smooth ring expansion to reveal the tetra*-ortho*-substituted biaryl subunit. Other bases, such as sodium hydride, triethylamine, or potassium *tert*-butoxide, also in combination with Lewis acids (e.g., titanium tetrachloride), were screened as well, but turned out to be inferior. Mechanistically the reaction is assumed to proceed via an intramolecular cyclopropane formation resulting from the nucleophilic attack of the ester enolate **I**. The attack of **I** can occur at either of the carbonyl functionalities of the1,3-indandione (indicated as pathway A or B in Scheme 1). As indicated for **II** (pathway A), collapse of the cyclopropane entails regioselective ring expansion and aromatization to give **5a** (42%) as the major product, together with its 1,4-naphthoquinone **5b** (7%), which was validated via single-crystal X-ray analysis. Quantitative conversion of **5a** to **5b** was achieved upon exposure of **5a** to ceric ammonium nitrate (CAN). For pathway B, attack of the enolate **I** occurred at the conjugated, and thus less reactive ketone. The obtained regiosimeric quinone **13** (24%) features an undesired orientation of the methoxy group with respect to the biaryl axis. The corresponding hydroquinone was also isolated in traces (for details, see the Supporting Information). It should be noted that attempts to construct the crucial biaryl unit employing a Heck reaction, conjugate addition, or Diels−Alder chemistry failed in our hands (see the Supporting Information for a graphical summary).

Having obtained the advanced biaryl **5b**, we proceeded to replace the aromatic bromide substituent. To our surprise, all attempts to directly install the carboxylic acid function were met with failure. Therefore, we chose to couple a furan as a masked carboxylic acid. To this end, a Suzuki−Miyaura coupling reaction of **5b** with potassium furan-2-trifluoroborate **14** provided furan **15** in 50% yield (Scheme 2). Analysis of the remaining material showed that the coupling reaction was accompanied by the formation of a naphtho[1,2-*b*]benzofuran byproduct in up to 10% yield (for details, see the Supporting Information). Despite screening different palladium catalysts, bases, and solvents, the formation of the byproduct could not be completely suppressed.

The ensuing oxidative cleavage of the furan with ruthenium tetroxide yielded the desired carboxylic acid **16** in up to 58% yield. In addition, an inseparable mixture of **16** and spirolactone **17a** was obtained. Structure elucidation through single-crystal X-ray analysis was crucial in revealing that **17a** has the opposite relative stereochemistry at C7 compared with that of chartspiroton (**1**). In contrast, **17b**, which possesses the desired stereochemistry at C7, was not obtained via the oxidative cleavage of the furan. Although it is possible that traces of **17b** were also formed, we did not detect it in the crude NMR. While the isolation of **17a** demonstrated the possibility of accessing the spiro-lactone moiety in a single step, the incorrect stereochemistry required further investigation of the oxidative spiro-lactonization.

Upon exposure of naphthoquinone **16** to epoxidation conditions (hydrogen peroxide, sodium carbonate), direct formation of the spiro-lactones **17a** and **17b** was observed (Scheme 3). The corresponding epoxides were not observed under these conditions.

Unfortunately, spiro-lactone **17a** featuring the incorrect stereochemistry at C7 was favored over **17b** (**17a**/**17b** = 2:1). Nonetheless, subjecting the minor spiro-lactone **17b** to boron tribromide led to global deprotection of the methyl and the benzyl ether to give chartspiroton (**1**) as the sole product in 43% yield. In line with our expectations, exposure of spirolactone **17a** to the same conditions produced 7-*epi*-chartspiroton (**18**). After purification, **18** underwent spontaneous conversion into chartspiroton (**1**) upon standing in deuterated dimethyl sulfoxide (DMSO-*d*_6_). We assume that the free phenol assists in isomerization via reversible lactone opening to furnish *ortho*-quinone methide **19**, which can reclose to form chartspiroton (**1**).^27^ Spectroscopic data for synthetic chartspiroton (**1**) were identical in all respects to those reported in the literature.^1^

In summary, we have successfully achieved the first reported synthesis of the natural product chartspiroton through an **11**-step linear sequence. To establish the tetra*-ortho*-substituted biaryl unit, we employed a base-induced ring expansion reaction of a 2,2-disubstituted-1,3-indandione. The unique spiro-fused benzofuran lactone moiety of the natural product could be installed by a late-stage oxidative cyclization sequence. Additionally, we could demonstrate that the spirolactone with the incorrect relative stereochemistry spontaneously equilibrates at the stage of the phenol to give chartspiroton. Future studies will explore the ring expansion of related indandiones and their use for the synthesis of glycosylated congeners.

## Supplementary Material

The Supporting Information is available free of charge at https://pubs.acs.org/doi/10.1021/acs.orglett.4c00695.

Experimental procedures, characterization data, NMR spectra, and crystallographic data (PDF)

SI

## Figures and Tables

**Figure 1:**
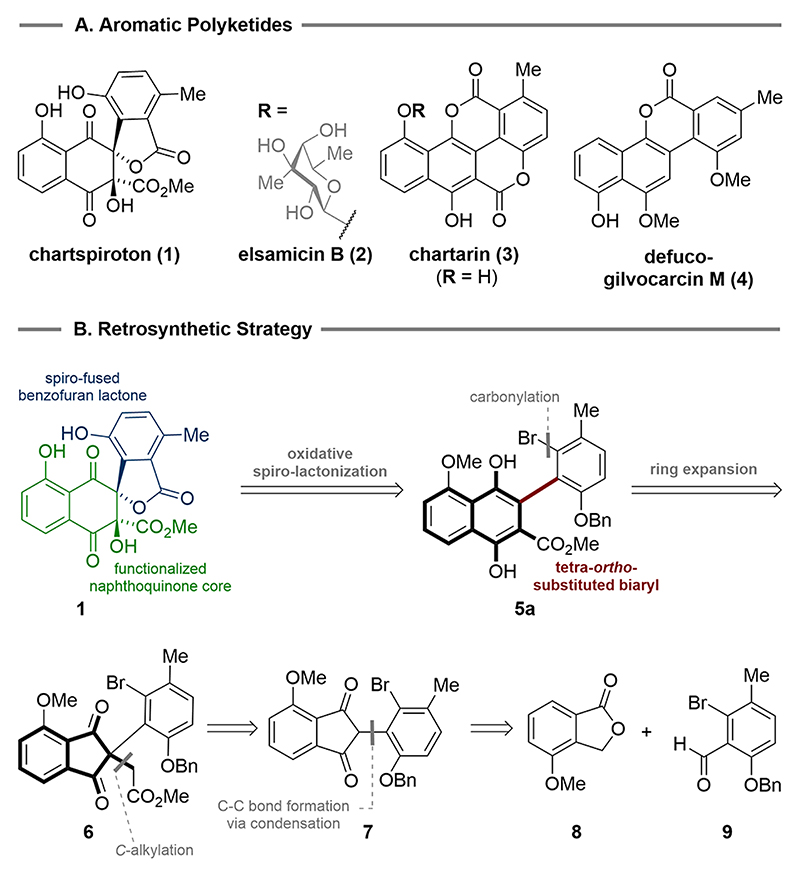
Selected structures of aromatic polyketide natural products and retrosynthetic strategy for chartspiroton (1).

**Scheme 1:**
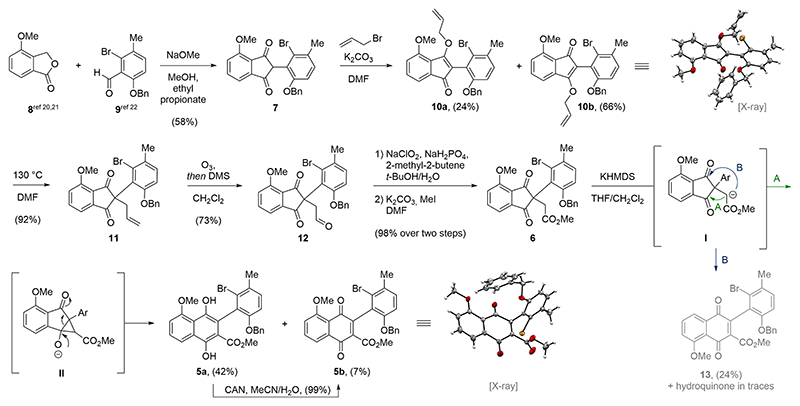
Installation of the tetra-*ortho*-substituted biaryl subunit through ring expansion of a 2,2-disubstituted 1,3-indandione.

**Scheme 2:**
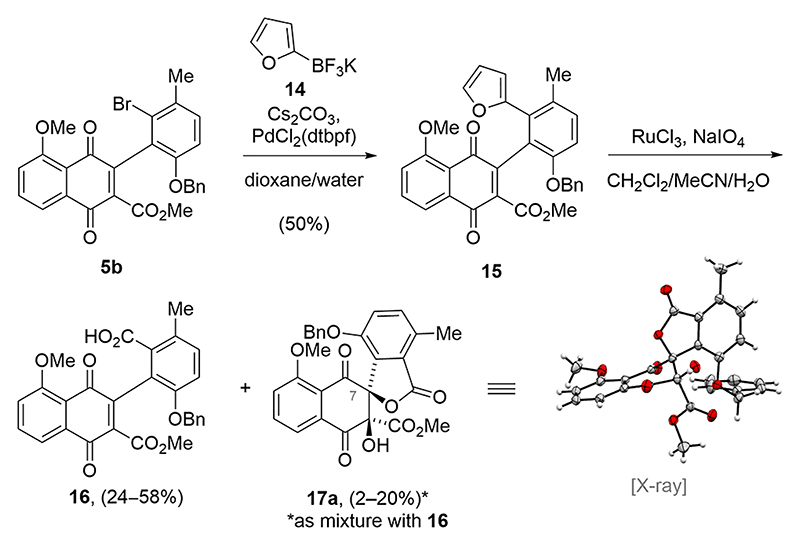
First observation of spiro-lactonization.

**Scheme 3:**
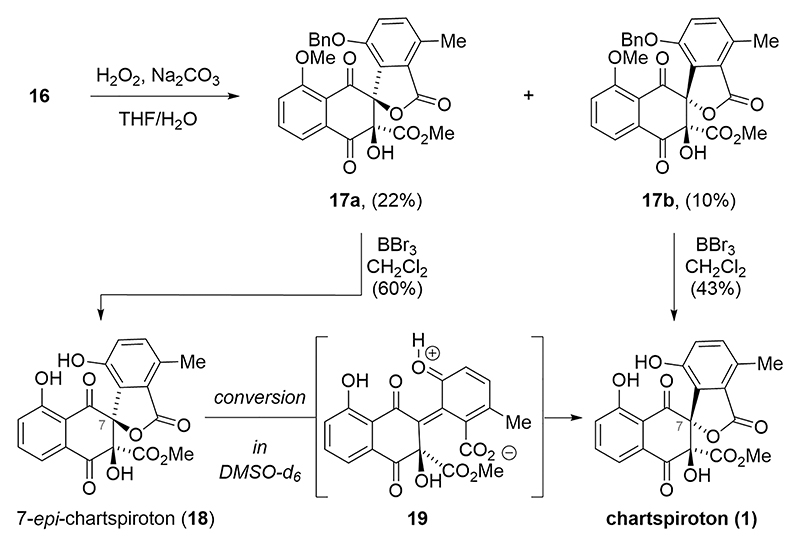
Completion of the synthesis of chartspiroton (1).

## Data Availability

The data underlying this study are available in the published article and its Supporting Information.
