# Correction to Synthesis of the Tetracyclic Spiro-naphthoquinone Chartspiroton

**DOI:** 10.1021/acs.orglett.4c01557

**Published:** 2024-06-03

**Authors:** Liesa Röder, Ivica Zamarija, Klaus Wurst, Thomas Magauer

**Affiliations:** † Department of Organic Chemistry and Center for Molecular Biosciences, University of Innsbruck, 6020 Innsbruck, Austria; ‡ Department of General Inorganic and Theoretical Chemistry, University of Innsbruck, 6020 Innsbruck, Austria

It has come to our attention that Dr. Ivica Zamarija (https://orcid.org/0000-0003-1290-9556) has also contributed to the realization of this work. Consequently, we wish to add him to the author list.

