# Supplementary material for: Synthesis of the Tetracyclic Spiro-naphthoquinone Chartspiroton
Source: Org Lett. Author manuscript; Available in PMC 2024 Apr 29. (PMC11041114; doi:10.1021/acs.orglett.4c00695)
Supplement: SI [file EMS195552-supplement-SI.pdf]

Supporting Information

Synthesis of the Tetracyclic Spiro-Naphthoquinone Chartspiroton.

Liesa Röder[a], Klaus Wurst[b], and Thomas Magauer[a*]

[a]*Department of Organic Chemistry and Center for Molecular Biosciences, University of Innsbruck, Innrain 80–82, 6020 Innsbruck, Austria.*
[b]*Department of General, Inorganic and Theoretical Chemistry, University of Innsbruck, 6020 Innsbruck, Austria.*
*Email: thomas.magauer@uibk.ac.at

Table of Contents

1. General Experimental Details S3

2. Experimental Procedures and Spectroscopic Data S6

2.1. Experimental Procedures S6

2.2. Overview of Failed Routes to the Biaryl S28

2.3. Spectroscopic Data S29

3. Crystallographic Data S49

1. General Experimental Details

1.1. General Working Methods

All reactions were carried out with magnetic stirring and, if moisture or air sensitive, under nitrogen or argon atmosphere using standard Schlenk techniques in oven-dried glassware (130 °C oven temperature). If required, glassware was further dried under vacuum (0.1 mmHg) with a heat-gun at 650 °C. Air- and moisture-sensitive liquids were transferred via syringe or stainless-steel cannula through rubber septa. Solids were added under inert gas counter flow or were dissolved in appropriate solvents. External bath thermometers were used to record all reaction temperatures. Low temperature reactions were carried out in a Dewar vessel filled with acetone and dry ice (–78 °C) or equipped with an electronically regulated cryostat in acetone (between –78 °C and 0 °C) or with distilled water and ice (0 °C). High temperature reactions were conducted in reaction vessels equipped with a reflux condenser or in a pressure tube using a heated silicon oil bath or a metal block. The reactions were magnetically stirred and monitored by NMR spectroscopy or analytical thin-layer chromatography (TLC), using aluminum plates percolated with silica gel (0.25 mm, 60 Å pore size, *Merck*) impregnated with a fluorescent indicator (254 nm). TLC plates were visualized by exposure to ultraviolet light (UV) at 254 nm, were stained by submersion in aqueous potassium permanganate solution ($KMnO_4$) or ceric ammonium molybdate solution (CAM), and were developed by heating with a heat-gun. Flash column chromatography (FCC) was performed as described by Still[1] employing silica gel (60 Å, 40–63 µm, *Merck KGaA*). The yields refer to chromatographically and spectroscopically (^{1}H and ^{13}C NMR) pure material, unless otherwise specified.

1.2. Materials

Tetrahydrofuran (THF) and diethyl ether (Et_2O) were purchased from Merck as “anhydrous” and dried over molecular sieves (4 Å) prior to use. All other solvents were purchased from Acros Organics as ‘extra dry’ reagents. If required solvents were degassed by freeze-pump-thaw under vacuum (0.1 mmHg). All other reagents with a purity > 95% were obtained from commercial sources (Sigma Aldrich, TCI, Acros Organics, Alfa Aesar, Strem Chemicals, BLDpharm, Fisher Scientific, ABCR and others) and used without further purification unless otherwise stated. Solvents for extraction, crystallization and flash column chromatography were purchased in technical grade and distilled under reduced pressure prior to use. Lithium bromide was dried at 160 °C under vacuum (0.1 mmHg) for 12 h; the hot, dried solid was flame dried under vacuum (0.1 mmHg) for 4−5 minutes immediately prior to use. The molarity of *n*-butyllithium and *t*-butyllithium solutions was determined by titration against diphenylacetic acid as an indicator (average of three determinations).[2]

[1] W.C. Still, M. Kahn, A. J. Mitra, *J Org. Chem.* **1978**, *43*, 2923.
[2] W. G. Kofron, L. M. Baclawski, *J. Org. Chem.* **1976**, *41*, 1879.

1.3. NMR Spectroscopy

NMR spectra (^{1}H NMR and ^{13}C NMR) were recorded in deuterated chloroform (chloroform-*d*) or deuterated dimethyl sulfoxide (DMSO-d_6) on a Bruker Avance Neo 400 MHz spectrometer, a Bruker Avance II 600 MHz spectrometer, or a Bruker 700 spectrometer at the Institute of Organic Chemistry and Center for Molecular Biosciences, University of Innsbruck and are reported as follows: chemical shift δ in ppm (multiplicity, coupling constant *J* in Hz, number of protons) for ^{1}H NMR spectra and chemical shift δ in ppm for ^{13}C NMR spectra. Multiplicities are abbreviated as follows: s = singlet, d = doublet, t = triplet, q = quartet, p = quintet, br = broad, m = multiplet, or combinations thereof. In case of combined multiplicities, the multiplicity with the larger coupling constant is stated first. Expect for multiplets, the chemical shift of all signals, as well for centrosymmetric multiplets, is reported as the center of the resonance range. For ^{1}H NMR the residual protic solvent peak served as internal reference (chloroform-*d*: 7.26 ppm, DMSO-d_6: 2.50 ppm). For ^{13}C NMR the central carbon resonance of chloroform-*d* (77.16 ppm) or DMSO-d_6 (39.52 ppm) served as internal reference. NMR spectra were assigned using information ascertained from homonuclear correlation spectroscopy (COSY), heteronuclear multiple bond coherence (HMBC), heteronuclear single quantum coherence (HSQC) and nuclear Overhauser enhancement spectroscopy (NOESY) experiments. All raw fid files were processed, and the spectra analyzed using the software MestReNova 12.0.1 from Mestrelab Research S. L.

1.4. Mass Spectrometry

High resolution mass spectra (HRMS) as ESI-HRMS were recorded on a Thermo Scientific™ QExactive™ Orbitrap Mass Spectrometer at the Institute of Organic Chemistry and Center for Molecular Biosciences, University of Innsbruck. DESI-HRMS as well as LTP-HRMS were recorded on a Thermo Scientific™ LTQ Orbitrap XL™ Hybrid Ion Trap-Orbitrap Mass Spectrometer equipped with a 3-in-1 ambient ionization interface[3] at the Institute of Organic Chemistry and Center for Molecular Biosciences, University of Innsbruck.

1.5. IR Spectroscopy

Infrared spectra (IR) were recorded from 4000 cm^{-1} to 450 cm^{-1} on a PerkinElmer Spectrum BX II FT-IR system. Samples were prepared as a neat film or a film by evaporation of a solution in chloroform-*d*. IR data in frequency of absorption (cm^{-1}) is reported as follows: w = weak, m = medium, s = strong, br = broad or combinations thereof.

1.6. Optical Rotation

Optical rotation values were recorded on a Schmidt+Haensch UniPol L1000 Peltier polarimeter. The specific rotation is calculated as follows:

$$[\alpha]_{\lambda}^{\varphi} = \frac{[\alpha] \cdot 100}{c \cdot d}$$

[3] C. Meisenbichler, F. Kluibenschedl, T. Müller, *Anal. Chem.* **2020**, *92* (21), 14314–14318.

Thereby, the wavelength λ is reported in nm and the measuring temperature ϕ in °C. α represents the recorded optical rotation at the apparatus, *c* the concentration of the analyte in 10 mg/mL and *d* the length of the cuvette in dm. Thus, the specific rotation is given in $10^{-1}\cdot deg\cdot cm^{2}\cdot g^{-1}$. Usage of the sodium D line (*λ* = 589 nm) is indicated by D instead of the wavelength in nm. The respective concentration as well as the solvent is reported at the relevant section of the experimental section.

1.7. Melting Points

Melting points were determined on an SRS MPA120 EZ-Melt melting point apparatus in open glass capillaries and are uncorrected.

1.8. X-Ray Diffraction Analysis

X-Ray diffraction analysis was carried out by Dr. Klaus Wurst at the Institute of Inorganic and Theoretical Chemistry and Center for Molecular Biosciences, University of Innsbruck. The data collections were performed on a Bruker D8 Quest diffractometer (Photon 100 detector) equipped with a microfocus source generator (Incoatec GmbH, Geesthacht, Germany) combined with multi-layer optics (monochromatized Mo *Kα* radiation, *λ* = 71.073 pm). The Bruker Apex III software was applied for the integration, scaling and multi-scan absorption correction of the data. The structure was solved with SHELXS (version 2013/1).[4] Structure refinement (full-matrix least-squares against F^2) with SHELXL (version 2014/7).[5] All non-hydrogen atoms were refined anisotropically. The hydrogen atoms were placed in ideal geometry riding on their parent atoms. Further details are summarized in the tables at the different sections. Plotting of thermal ellipsoids in this document was carried out using MERCURY for Windows at 50% probability level.

[4] G. M. Sheldrick, *Acta Crystallogr. Sect. Found. Adv.* **2015**, *71*, 3–8.
[5] G. M. Sheldrick, *Acta Crystallogr. Sect. C Struct. Chem.* **2015**, *71*, 3–8.

2. Experimental Procedures and Spectroscopic Data

2.1. Experimental Procedures

4-Hydroxyisobenzofuran-1(3*H*)-one S2

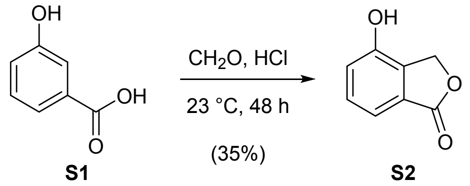

Based on a slightly modified literature procedure,[6] 3-hydroxybenzoic acid **S1** (22.5 g, 163 mmol, 1 equiv) was suspended in a solution of formaldehyde (37wt% in water, 450 mL, 6.00 mol, 37.0 equiv) and concentrated hydrochloric acid (450 mL) at 23 °C. After 48 hours, the reaction was filtered, and the crude product was collected as beige solid by filtration. The product was purified by recrystallization from water (350 mL), followed by flash column chromatography on silica gel (3% methanol in dichloromethane, grading to 30% methanol in dichloromethane) to give hydroxy isobenzofuranone **S2** (8.58 g, 57.2 mmol, 35%) as a colourless solid. The obtained analytical data were in full agreement with those reported in the literature.[6]

4-Methoxyisobenzofuran-1(3*H*)-one 8

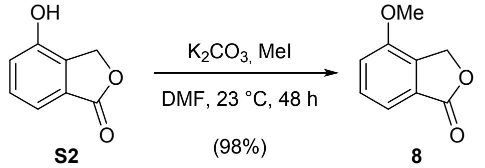

Based on a slightly modified literature procedure,[7] methyl iodide (5.24 mL, 83.9 mmol, 1.20 equiv) was added to a solution of potassium carbonate (19.3 g, 140 mmol, 2.00 equiv) and hydroxy isobenzofuranone **S2** (10.5 g, 69.9 mmol, 1 equiv) in *N*,*N*-dimethylformamide (160 mL) at 23 °C. After 48 hours, the solvent was removed under reduced pressure and the residue was dissolved in water (100 mL). The aqueous layer was extracted with ethyl acetate (3 × 150 mL) and the combined organic layers were washed with an aqueous lithium chloride solution (10wt%, 200 mL). The washed solution was dried over magnesium sulfate, the dried solution was filtered and the filtrate was concentrated under reduced pressure. The residue was purified by flash column chromatography on silica gel (30% ethyl acetate in cyclohexane, grading to 60% ethyl acetate in cyclohexane) to yield methoxyisobenzofuranone

[6] Z.-G. Feng, G. L. Burnett, T. R. R. Pettus, *Synlett* **2018**, *29*, 1517–1519.
[7] ASTRAZENECA PLC - US2009/192156, **2009**, A1.

8 (11.3 g, 68.8 mmol, 98%) as a colorless solid. The obtained analytical data were in full agreement with those reported in the literature.[8]

2-Bromo-6-fluoro-3-methylbenzaldehyde S4

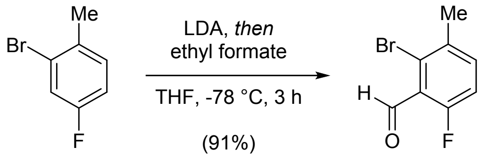

S3 **S4**

Based on a slightly modified literature procedure,[9] a solution of *n*-Butyllithium (2.50 M in hexanes, 41.6 mL, 104 mmol, 1.31 equiv) was added dropwise to a solution of diisopropylamine (15.0 mL, 107 mmol, 1.35 equiv) in tetrahydrofuran (47 mL) at −78 °C. The reaction was stirred at −78 °C for 20 min and was then allowed to warm up to 0 °C. After further 20 min, the freshly prepared lithium diisopropylamide solution (1.03 M in tetrahydrofuran, 92.2 mL, 94.9 mmol, 1.20 equiv) was added dropwise to a solution of fluorobenzene **S3** (10.0 mL, 79.1 mmol, 1 equiv) in tetrahydrofuran (392 mL) at −78 °C. After two hours, ethyl formate (19.6 mL, 237 mmol, 3.00 equiv) was added dropwise over 30 minutes with a syringe pump at −78 °C. Two and a half hours upon the addition of ethyl formate, water (296 mL) was added at −78 °C and the resulting biphasic reaction mixture was allowed to warm up to 23 °C. The layers were separated, and the aqueous layer was extracted with ethyl acetate (3 × 200 mL). The combined organic layers were washed with a saturated aqueous solution of sodium chloride (200 mL). The washed solution was dried over magnesium sulfate, the dried solution was filtered and the filtrate was concentrated under reduced pressure. The residue was purified by flash column chromatography on silica gel (5% diethyl ether in petroleum ether, grading to 10% diethyl ether in petroleum ether) to yield benzaldehyde **S4** (15.6 g, 72.1 mmol, 91%) as a yellow solid. The obtained analytical data were in full agreement with those reported in the literature.[9]

2-Bromo-6-hydroxy-3-methylbenzaldehyde S5

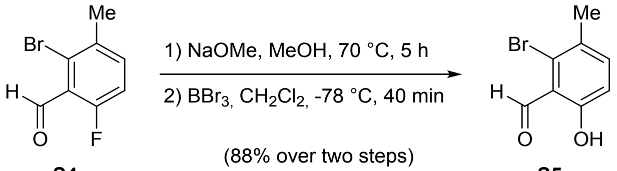

S4 **S5**

Based on a slightly modified literature procedure,[9] sodium metal (6.63 g, 289 mmol, 4.00 equiv) was added in small pieces to an oven dried flask under argon. Afterwards, methanol (66 mL) was slowly

[8] B. A. Egan, M. Paradowski, L. H. Thomas, R. Marquez, *Org. Lett.* **2011**, *13* (8), 2086–2089.
[9] WO 2017/151489 A1, September 08, **2017**.

added with a syringe at 0 °C. Upon addition, the cooling bath was removed, and the solution was stirred at 23 °C until all sodium pieces were completely dissolved.
In a second oven dried flask under argon, benzaldehyde **S4** (15.6 g, 72.1 mmol, 1 equiv) was dissolved in methanol (294 mL) at 23 °C. To this solution, the freshly prepared sodium methoxide solution was added with a syringe at 23 °C. Upon addition, the reaction was stirred at 70 °C. After five hours, the reaction was allowed to cool down to 23 °C and the solvent was removed under reduced pressure. The residue was dissolved in water (100 mL) and the aqueous layer was extracted with ethyl acetate (3 × 150 mL). The combined organic layers were washed with a saturated aqueous solution of sodium chloride (150 mL). The washed solution was dried over magnesium sulfate, the dried solution was filtered and the filtrate was concentrated under reduced pressure to give the crude methoxy benzaldehyde as a colorless solid (16.4 g, 71.6 mmol), which was used in the next step without further purification.

To a solution of methoxy benzaldehyde (16.4 g, 71.6 mmol, 1 equiv) in dichloromethane (200 mL) was slowly added a solution of boron tribromide (1.00 M in dichloromethane, 93.1 mL, 93.1 mmol, 1.3 equiv) at −78 °C. After one and a half hour, water (75 mL) was added at −78 °C and the resulting biphasic reaction mixture was allowed to warm up to 23 °C. The layers were separated, and the aqueous layer was extracted with dichloromethane (3 × 100 mL). The combined organic layers were washed with a saturated aqueous solution of sodium chloride (150 mL). The washed solution was dried over magnesium sulfate, the dried solution was filtered and the filtrate was concentrated under reduced pressure. The residue was purified by flash column chromatography on silica gel (5% diethyl ether in petroleum ether) to yield phenol **S5** (13.6 g, 63.2 mmol, 88% over two steps) as a colorless solid. The obtained analytical data were in full agreement with those reported in the literature.[9]

6-(Benzyloxy)-2-bromo-3-methylbenzaldehyde 9

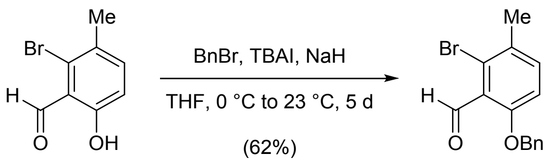

S5 **9**

Sodium hydride (60% dispersion in mineral oil, 1.90 g, 48.4 mmol, 1.30 equiv) was added to a solution of phenol **S5** (8.00 g, 37.2 mmol, 1 equiv) in tetrahydrofuran (365 mL) at 0 °C. After 15 min, tetra-*n*-butylammonium iodide (4.12 g, 11.2 mmol, 30.0 mol%) and benzyl bromide (5.75 mL, 48.4 mmol, 1.30 equiv) were successively added at 0 °C. The reaction was stirred in the dark and was allowed to warm up to 23 °C. After five days, a saturated aqueous solution of ammonium chloride (200 mL) was added at 23 °C. The layers were separated, and the aqueous layer was extracted with ethyl acetate (3 × 150 mL). The combined organic layers were washed with a saturated aqueous solution of sodium chloride (150 mL). The washed solution was dried over magnesium sulfate, the dried solution was

filtered and the filtrate was concentrated under reduced pressure. The residue was purified by flash column chromatography on silica gel (5% diethyl ether in petroleum ether, grading to 10% diethyl ether in petroleum ether) to yield benzaldehyde **9** (7.00 g, 22.9 mmol, 62%) as a pale-yellow solid.

TLC (50% diethyl ether in petroleum ether): $R_f = 0.51$ (UV, $KMnO_4$).

mp: 83 – 85 °C.

^{1}H NMR (400 MHz, chloroform-*d*) δ 10.47 (s, 1H), 7.45 – 7.35 (m, 4H), 7.35 – 7.29 (m, 2H), 6.91 (d, *J* = 8.5 Hz, 1H), 5.15 (s, 2H), 2.37 (s, 3H).

^{13}C NMR (101 MHz, chloroform-*d*) δ 191.1, 158.7, 136.1, 135.3, 131.9, 128.8, 128.2, 127.2, 126.4, 124.8, 112.4, 71.1, 22.6.

IR (Diamond-ATR, neat) $\tilde{\upsilon}_{max}$: 3032 (w), 2922 (w), 2867 (w), 1692 (s), 1590 (m), 1563 (s), 1451 (s), 1414 (w), 1379 (s), 1265 (s), 1188 (m), 1079 (w), 1012 (s), 941 (w), 877 (w), 849 (w), 806 (m), 733 (s), 696 (s), 659 (w), 606 (w), 502 (w), 463 (w) cm^{-1}.

HRMS (ESI) calc. for $C_{15}H_{13}BrNaO_2$ $[M+Na]^+$: 326.9991; found: 326.9989.

2-(6-(Benzyloxy)-2-bromo-3-methylphenyl)-4-methoxy-1*H*-indene-1,3(2*H*)-dione 7

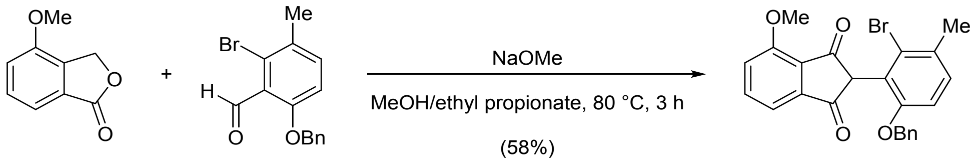

Based on a slightly modified literature procedure,[10] sodium metal (6.22 g, 271 mmol, 3.94 equiv) was added in small pieces to an oven dried flask under argon atmosphere. Afterwards, methanol (168 mL) was slowly added with a syringe at 0 °C. Upon addition, the cooling bath was removed, and the solution was stirred at 23 °C until all sodium pieces were completely dissolved. In a second oven dried flask under argon, methoxyisobenzofuranone **8** (11.3 g, 68.7 mmol, 1 equiv) and benzaldehyde **9** (20.9 g, 68.7 mmol, 1.00 equiv) were dissolved in ethyl propionate (480 mL) at 23 °C. To this solution, the freshly prepared sodium methoxide solution was added with a syringe at 23 °C. Upon addition, the reaction was stirred at 50 °C. After three hours, the reaction was allowed to cool down to 23 °C and the solvent was removed under reduced pressure. The residue was dissolved in water (300 mL) and the aqueous layer was acidified with acetic acid (pH = 3−4) and stirred for ten minutes at 23 °C. The acidified aqueous layer was extracted with ethyl acetate (3 × 300 mL) and the combined organic layers

[10] S. L. Shapiro, K. Geiger, L. Freedman, *J. Org. Chem.* **1960**, *25* (11), 1860–1865.

were washed with a saturated aqueous solution of sodium chloride (400 mL). The washed solution was dried over magnesium sulfate, the dried solution was filtered and the filtrate was concentrated under reduced pressure. The residue was purified by flash column chromatography on silica gel (20% ethyl acetate in cyclohexane, grading to 100% ethyl acetate in cyclohexane) to yield 1,3-indandione **7** (17.9 g, 39.6 mmol, 58%*) as a colorless solid.

*Despite subjecting 1,3-indandione **7** to purification by flash column chromatography on silica gel, complete purification remained elusive. However, the minor remaining impurities were separated during the subsequent transformation.

TLC (50% ethyl acetate in cyclohexane): $R_f = 0.44$ (UV, $KMnO_4$).

mp: 128 − 130 °C.

^{1}H NMR (400 MHz, chloroform-*d*) δ 7.59 – 7.54 (m, 1H), 7.36 – 7.33 (m, 1H), 7.17 – 7.14 (m, 1H), 7.11 – 7.08 (m, 1H), 7.07 – 7.01 (m, 3H), 6.93 – 6.89 (m, 2H), 6.72 (d, *J* = 8.4 Hz, 1H), 5.08 (s, 1H), 4.69 (q, *J* = 10.8 Hz, 2H), 3.93 (s, 3H), 2.42 (s, 3H).

^{13}C NMR (101 MHz, chloroform-*d*) δ 198.6, 195.8, 157.1, 154.3, 143.3, 136.9, 135.3, 131.3, 130.7, 130.5, 128.2, 127.9, 127.7, 127.6, 123.9, 117.3, 114.7, 110.4, 71.0, 60.3, 56.3, 23.8.

IR (Diamond-ATR, neat) $\tilde{\upsilon}_{max}$: 2938 (w), 2887 (w), 1747 (w), 1712 (s), 1598 (m), 1481 (m), 1381 (w), 1353 (w), 1286 (s), 1226 (w), 1186 (w), 1110 (w), 1047 (w), 1010 (m), 945 (w), 912 (w), 800 (w), 730 (w), 698 (w), 671 (w), 606 (w) cm^{-1}.

HRMS (ESI) calc. for $C_{24}H_{20}BrO_4$ $[M+H]^+$: 451.0539; found: 451.0533.

3-(Allyloxy)-2-(6-(benzyloxy)-2-bromo-3-methylphenyl)-7-methoxy-1*H*-inden-1-one 10b
3-(Allyloxy)-2-(6-(benzyloxy)-2-bromo-3-methylphenyl)-4-methoxy-1*H*-inden-1-one 10a

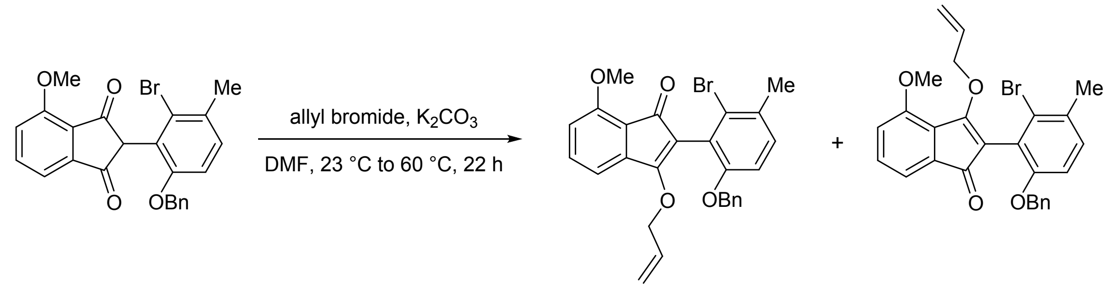

7 **10b** (66%) **10a** (24%)

Potassium carbonate (7.42 g, 53.7 mmol, 1.50 equiv) was added to a solution of 1,3-indandione **7** (16.2 g, 35.8 mmol, 1 equiv) in *N*,*N*-dimethylformamide (336 mL) at 23 °C. After 15 min, allyl bromide (4.03 mL, 46.6 mmol, 1.30 equiv) was added dropwise at 23 °C. Upon addition, the reaction was stirred

at 60 °C. After 22 h, the reaction was allowed to cool down to 23 °C and the solvent was removed under reduced pressure. The crude mixture was dissolved in water (150 mL) and the aqueous layer was extracted with ethyl acetate (3 × 150 mL). The combined organic layers were washed with a saturated aqueous solution of sodium chloride (200 mL). The washed solution was dried over magnesium sulfate, the dried solution was filtered and the filtrate was concentrated under reduced pressure. The residue was purified by flash column chromatography on silica gel (10% ethyl acetate in cyclohexane) to yield separated allyloxy indandione **10b** (11.67 g, 23.75 mmol, 66%) as a yellow solid and allyloxy indandione **10a** (4.13 g, 8.40 mmol, 24%) as a yellow-orange solid.

Allyloxy indandione 10b

TLC (50% ethyl acetate in cyclohexane): R_f = 0.56 (UV, $KMnO_4$).

mp: 145 − 147 °C.

^{1}H NMR (400 MHz, chloroform-*d*) δ 7.37 (dd, *J* = 8.5, 7.1 Hz, 1H), 7.32 – 7.20 (m, 5H), 7.15 – 7.12 (m, 1H), 7.01 – 6.99 (m, 1H), 6.93 – 6.90 (m, 1H), 6.79 (d, *J* = 8.4 Hz, 1H), 5.86 (ddt, *J* = 17.3, 10.7, 5.5 Hz, 1H), 5.25 – 5.17 (m, 2H), 5.04 (s, 2H), 4.49 – 4.38 (m, 2H), 3.95 (s, 3H), 2.37 (s, 3H).

^{13}C NMR (101 MHz, chloroform-*d*) δ 191.8, 169.2, 156.6, 155.4, 143.1, 137.2, 134.5, 132.3, 130.9, 130.8, 129.9, 128.6, 127.8, 127.0, 123.8, 118.4, 116.8, 115.4, 112.4, 111.9, 108.3, 71.3, 71.1, 56.4, 23.3.

IR (Diamond-ATR, neat) $\tilde{\upsilon}_{max}$: 3066 (w), 3031 (w), 2930 (w), 2843 (w), 1699 (s), 1633 (m), 1598 (m), 1478 (s), 1390 (m), 1318 (s), 1285 (m), 1229 (w), 1163 (w), 1107 (w), 1058 (s), 1017 (m), 969 (w), 895 (w), 866 (w), 802 (w), 738 (w), 697 (w), 586 (w), 423 (w) cm^{-1}.

HRMS (ESI) calc. for $C_{27}H_{24}BrO_4$ $[M+H]^+$: 491.0852; found: 491.0855.

Crystal structure: 10b precipitate as crystalline material in a solvent mixture of 10% ethyl acetate in cyclohexane after being purified by flash column chromatography on silica gel.
See chapter 3, page S49 for more details.

Allyloxy indandione 10a

TLC (50% ethyl acetate in cyclohexane): R_f = 0.61 (UV, $KMnO_4$).

mp: 114 − 116 °C.

^{1}H NMR (400 MHz, chloroform-*d*) δ 7.33 – 7.22 (m, 6H), 7.18 (dd, *J* = 7.1, 0.8 Hz, 1H), 7.15 – 7.12 (m, 1H), 7.03 – 7.00 (m, 1H), 6.79 (d, *J* = 8.4 Hz, 1H), 5.85 (ddt, *J* = 17.2, 10.7, 4.7 Hz, 1H), 5.40 (dq, *J* = 17.2, 1.8 Hz, 1H), 5.21 (dq, *J* = 10.6, 1.6 Hz, 1H), 5.05 (s, 2H), 4.48 (qdt, *J* = 13.4, 4.7, 1.7 Hz, 2H), 3.92 (s, 3H), 2.37 (d, *J* = 0.6 Hz, 3H).

^{13}C NMR (101 MHz, chloroform-*d*) δ 193.1, 173.7, 156.5, 153.6, 137.1, 134.7, 132.1, 132.0, 130.9, 130.8, 130.0, 128.6, 127.8, 127.1, 125.1, 124.1, 118.0, 117.2, 114.7, 111.8, 107.6, 71.6, 71.0, 56.3, 23.3.

IR (Diamond-ATR, neat) $\tilde{\upsilon}_{max}$: 3064 (w), 3031 (w), 2942 (w), 2839 (w), 1699 (m), 1619 (s), 1584 (m), 1480 (m), 1396 (m), 1320 (s), 1272 (s), 1177 (w), 1113 (w), 1050 (w), 1014 (m), 965 (m), 903 (w), 870 (w), 804 (w), 763 (w), 736 (w), 697 (w), 623 (w), 575 (w), 540 (w) cm^{-1}.

HRMS (ESI) calc. for $C_{27}H_{24}BrO_4$ $[M+H]^+$: 491.0852; found: 491.0858.

2-Allyl-2-(6-(benzyloxy)-2-bromo-3-methylphenyl)-4-methoxy-1*H*-indene-1,3(2*H*)-dione 11

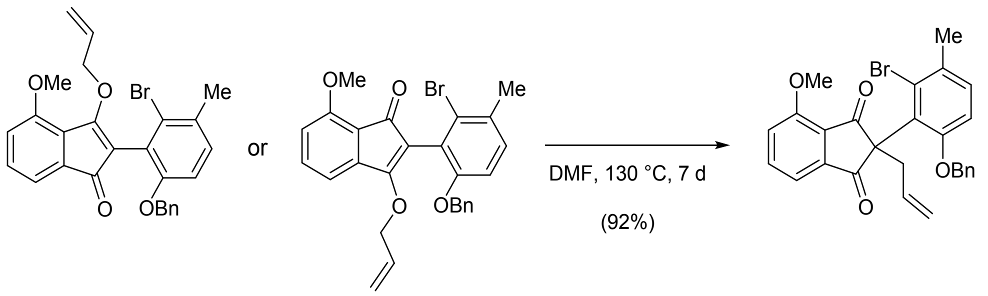

10a **10b** **11**

Allyloxy indandione **10a** (4.00 g, 8.14 mmol, 1 equiv) or **10b** (4.00 g, 8.14 mmol, 1 equiv) respectively was dissolved in *N*,*N*-dimethylformamide (225 mL) in a pressure vial at 23 °C. The vial was flushed with argon, sealed and the reaction was stirred at 130 °C. After seven days, the reaction was allowed to cool down to 23 °C and the solvent was removed under reduced pressure. The residue was purified by flash column chromatography on silica gel (20% ethyl acetate in cyclohexane) to yield *C*-alkylated 1,3-indandione **11** (3.69 g, 7.51 mmol, 92%) as a yellow solid.

TLC (50% ethyl acetate in cyclohexane): $R_f = 0.53$ (UV, $KMnO_4$).

mp: 43 – 45 °C.

^{1}H NMR (400 MHz, chloroform-*d*) δ 7.57 – 7.51 (m, 1H), 7.31 – 7.28 (m, 1H), 7.21 – 7.16 (m, 3H), 7.14 – 7.08 (m, 3H), 7.06 (d, *J* = 8.2 Hz, 1H), 6.77 (d, *J* = 8.4 Hz, 1H), 5.78 – 5.67 (m, 1H), 4.93 – 4.85 (m, 2H), 4.79 (s, 2H), 3.93 (s, 3H), 3.52 (dd, *J* = 14.7, 6.4 Hz, 1H), 3.22 (dd, *J* = 14.7, 7.1 Hz, 1H), 2.36 (s, 3H).

^{13}C NMR (101 MHz, chloroform-*d*) δ 200.4, 198.6, 157.3, 157.2, 142.7, 136.8, 135.7, 133.9, 133.3, 130.6, 128.5, 127.9, 127.8, 127.8, 126.9, 125.3, 118.3, 117.2, 114.9, 112.4, 71.6, 65.1, 56.3, 40.0, 24.8.

IR (Diamond-ATR, neat) $\tilde{\nu}_{max}$: 2926 (w), 1743 (w), 1706 (s), 1639 (w), 1600 (m), 1483 (m), 1453 (m), 1377 (w), 1283 (s), 1202 (m), 1100 (w), 1057 (w), 1004 (m), 949 (w), 922 (w), 851 (w), 806 (w), 733 (w), 696 (w), 608 (w), 502 (w), 426 (w) cm^{-1}.

HRMS (ESI) calc. for $C_{27}H_{24}BrO_4$ $[M+H]^+$: 491.0852; found: 491.0848.

2-(2-(6-(Benzyloxy)-2-bromo-3-methylphenyl)-4-methoxy-1,3-dioxo-2,3-dihydro-1*H*-inden-2-yl)acetaldehyde 12

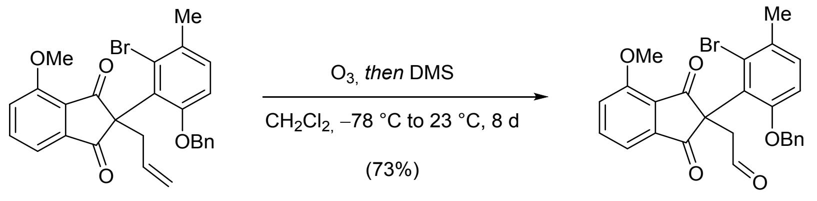

Ozone was bubbled through a solution of *C*-alkylated 1,3-indandione **11** (7.35 g, 14.9 mmol, 1 equiv) in dichloromethane (146 mL) at −78 °C. After one hour, the blue-greenish reaction solution was sparged with a stream of oxygen to remove any dissolved ozone. Dimethyl sulfide (4.43 mL, 59.9 mmol, 4.00 equiv) was added and the reaction was allowed to warm up to 23 °C. After eight days, the solvent was removed under reduced pressure and the residue was purified by flash column chromatography on silica gel (20% ethyl acetate in cyclohexane, grading to 25% ethyl acetate in cyclohexane) to yield aldehyde **12** (5.36 g, 10.9 mmol, 73%) as a yellow solid.

TLC (50% ethyl acetate in cyclohexane): $R_f = 0.41$ (UV, CAM, $KMnO_4$).

mp: 57 – 59 °C.

^{1}H NMR (400 MHz, chloroform-*d*) δ 9.80 (t, *J* = 1.5 Hz, 1H), 7.68 (dd, *J* = 8.2, 7.6 Hz, 1H), 7.43 (d, *J* = 7.4 Hz, 1H), 7.29 – 7.24 (m, 3H), 7.21 – 7.14 (m, 4H), 6.85 (d, *J* = 8.5 Hz, 1H), 4.92 (s, 2H), 3.98 (s, 3H), 3.28 (dd, *J* = 18.6, 1.5 Hz, 1H), 3.09 (dd, *J* = 18.6, 1.7 Hz, 1H), 2.32 (s, 3H).

^{13}C NMR (101 MHz, chloroform-*d*) δ 199.6, 199.0, 197.3, 157.9, 157.6, 141.6, 137.5, 135.4, 133.1, 131.3, 128.7, 128.2, 127.8, 127.7, 126.2, 123.8, 117.9, 115.7, 113.1, 71.5, 62.8, 56.4, 45.8, 24.5.

IR (Diamond-ATR, neat) $\tilde{\nu}_{max}$: 2924 (w), 2851 (w), 2038 (w), 1992 (w), 1708 (s), 1600 (m), 1483 (m), 1455 (w), 1373 (w), 1286 (m), 1200 (w), 1151 (w), 1059 (w), 1008 (m), 914 (w), 806 (w), 737 (w), 698 (w), 606 (w), 512 (w), 426 (w) cm^{-1}.

HRMS (ESI) calc. for $C_{26}H_{21}BrNaO_5$ $[M+Na]^+$: 515.0465; found: 515.0460.

Methyl 2-(2-(6-(benzyloxy)-2-bromo-3-methylphenyl)-4-methoxy-1,3-dioxo-2,3-dihydro-1*H*-inden-2-yl)acetate 6

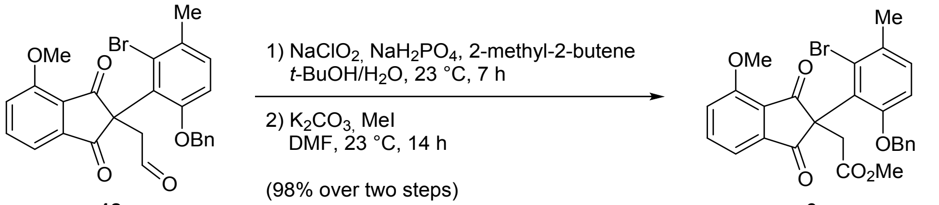

2-Methyl-2-butene (54.0 mL, 521 mmol, 48.0 equiv) was added to a solution of aldehyde **12** (5.36 g, 10.9 mmol, 1 equiv) in *tert*-butyl alcohol (306 mL) at 23 °C. Afterwards, a solution of sodium chlorite (9.04 g, 100 mmol, 9.20 equiv) and sodium dihydrogen phosphate (8.99 g, 75.0 mmol, 6.90 equiv) in water (128 mL) was added dropwise and the reaction was stirred at 23 °C. After seven hours, the volatile components were removed under reduced pressure and the residue was dissolved in water (100 mL). The aqueous layer was extracted with ethyl acetate (3 × 120 mL) and the combined organic layers were washed with a saturated aqueous solution of sodium chloride (160 mL). The washed solution was dried over magnesium sulfate, the dried solution was filtered and the filtrate was concentrated under reduced pressure to yield the carboxylic acid (5.53 g, 10.9 mmol) as a pale-yellow solid, which was used in the next step without further purification.

The unpurified carboxylic acid (5.53 g, 10.9 mmol, 1 equiv) was dissolved in *N*,*N*-dimethylformamide (105 mL) and potassium carbonate (2.25 g, 16.3 mmol, 1.50 equiv) was added at 23 °C. After ten minutes, methyl iodide (815 µL, 13.0 mmol, 1.20 equiv) was added and the reaction was stirred at 23 °C. After 14 h, the solvent was removed under reduced pressure. The crude residue was dissolved in an aqueous solution of lithium chloride (10wt%, 60 mL), and the aqueous layer was extracted with ethyl acetate (3 × 80 mL). The combined organic layers were washed with a saturated aqueous solution of sodium chloride (160 mL). The washed solution was dried over magnesium sulfate, the dried solution was filtered and the filtrate was concentrated under reduced pressure. The residue was purified by flash column chromatography on silica gel (20% ethyl acetate in cyclohexane, grading to 40% ethyl acetate in cyclohexane) to yield methyl ester **6** (5.57 g, 10.6 mmol, 98% over two steps) as a beige solid.

TLC (50% ethyl acetate in cyclohexane): $R_f = 0.33$ (UV, $KMnO_4$).

mp: 54 − 56 °C.

^{1}H NMR (400 MHz, chloroform-*d*) δ 7.57 (dd, *J* = 8.1, 7.6 Hz, 1H), 7.30 (dd, *J* = 7.5, 0.5 Hz, 1H), 7.25 – 7.22 (m, 3H), 7.14 – 7.06 (m, 4H), 6.72 (d, *J* = 8.4 Hz, 1H), 4.83 (q, *J* = 12.3 Hz, 2H), 3.90 (s, 3H), 3.89 (d, *J* = 16.7 Hz, 1H), 3.75 (d, *J* = 16.7 Hz, 1H), 3.44 (s, 3H), 2.34 (s, 3H).

^{13}C NMR (101 MHz, chloroform-*d*) δ 198.4, 196.2, 170.5, 157.4, 156.8, 143.3, 136.7, 136.1, 133.9, 130.8, 128.6, 128.0, 127.9, 127.4, 127.4, 125.0, 116.9, 115.1, 113.5, 72.1, 61.3, 56.2, 51.8, 39.4, 24.9.

IR (Diamond-ATR, neat) $\tilde{\upsilon}_{max}$: 3008 (w), 2951 (w), 2842 (w), 1736 (m), 1710 (s), 1600 (m), 1483 (m), 1453 (m), 1347 (w), 1286 (s), 1200 (m), 1059 (w), 1004 (m), 949 (w), 912 (w), 853 (w), 804 (w), 733 (w), 698 (w), 649 (w), 606 (w) cm^{-1}.

HRMS (ESI) calc. for $C_{27}H_{23}BrNaO_6$ $[M+Na]^+$: 545.0570; found: 545.0559.

Methyl 3-(6-(benzyloxy)-2-bromo-3-methylphenyl)-1,4-dihydroxy-5-methoxy-2-naphthoate 5a Methyl 3-(6-(benzyloxy)-2-bromo-3-methylphenyl)-5-methoxy-1,4-dioxo-1,4-dihydronaphthalene -2-carboxylate 5b

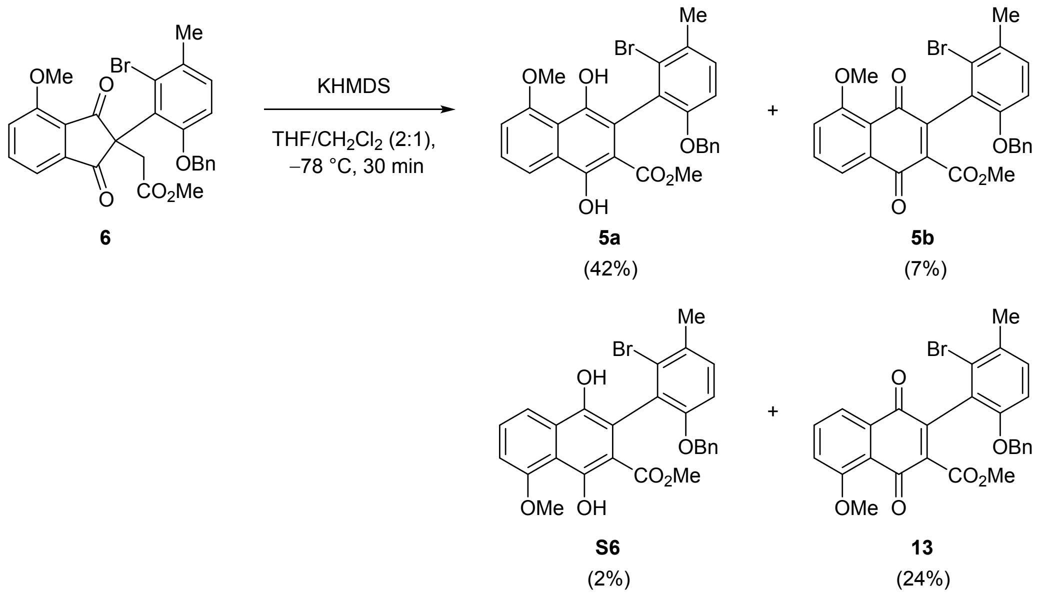

A solution of potassium bis(trimethylsilyl)amide (1.00 M in tetrahydrofuran, 450 µL, 449 µmol, 1.0 equiv) was added dropwise to a solution of methyl ester **6** (235 mg, 449 µmol, 1 equiv) in tetrahydrofuran (4 mL) and dichloromethane (2 mL) at −78 °C. Upon addition, the reaction was stirred for further 30 min, before water (4 mL) was added at −78 °C. The resulting biphasic reaction mixture was allowed to warm up to 23 °C and the layers were separated. The aqueous layer was extracted with ethyl acetate (3 × 6 mL) and the combined organic layers were washed with a saturated aqueous solution of sodium chloride (8 mL). The washed solution was dried over magnesium sulfate, the dried solution was filtered and the filtrate was concentrated under reduced pressure. The residue was purified by flash column chromatography on silica gel (10% ethyl acetate in cyclohexane, grading to 30% ethyl acetate in cyclohexane) to yield the desired dihydroxynaphthalene **5a** (99.3 mg, 190 µmol, 42%) as a yellow solid and naphthoquinone **5b** (17.1 mg, 32.8 µmol, 7%) as an orange solid, accompanied by recovered staring material **6** (46.8 mg, 89.4 µmol, 20%) as a yellow solid as well as naphthoquinone **13** (57.2 mg,

110 µmol, 24%) as an orange solid and dihydroxynaphthalene **S6** (4.3 mg, 8.2 µmol, 2%) as a yellow solid, with the last two possessing the undesired orientation of the methoxy group.

Dihydroxynaphthalene 5a

TLC (50% ethyl acetate in cyclohexane): R_f = 0.55 (UV, $KMnO_4$).

mp: 54 − 56 °C.

^{1}H NMR (700 MHz, chloroform-*d*) δ 12.00 – 11.98 (m, 1H), 9.19 – 9.17 (m, 1H), 8.16 – 8.13 (m, 1H), 7.46 – 7.43 (m, 1H), 7.20 – 7.16 (m, 3H), 7.14 – 7.09 (m, 3H), 7.02 – 7.00 (m, 1H), 6.86 – 6.84 (m, 1H), 5.02 – 4.97 (m, 2H), 4.02 (s, 3H), 3.42 (s, 3H), 2.41 (s, 3H).

^{13}C NMR (176 MHz, chloroform-*d*) δ 171.9, 155.9, 154.8, 154.2, 143.4, 137.6, 130.7, 130.5, 129.1, 128.4, 127.5, 127.5, 127.3, 126.9, 126.1, 118.5, 118.1, 117.1, 112.0, 108.5, 107.1, 70.9, 56.4, 52.1, 23.2.

IR (Diamond-ATR, neat) $\tilde{\upsilon}_{max}$: 3393 (m), 2948 (w), 1736 (w), 1655 (m), 1604 (m), 1453 (s), 1373 (s), 1318 (m), 1283 (m), 1232 (s), 1163 (m), 1102 (w), 1063 (m), 998 (m), 953 (w), 914 (w), 886 (w), 843 (w), 792 (m), 753 (m), 698 (w), 585 (w) cm^{-1}.

HRMS (ESI) calc. for $C_{27}H_{23}BrNaO_6$ $[M+Na]^+$: 545.0570; found: 545.0562.

Naphthoquinone 5b

TLC (50% ethyl acetate in cyclohexane): R_f = 0.43 (UV, $KMnO_4$).

mp: 162 − 164 °C.

^{1}H NMR (400 MHz, chloroform-*d*) δ 7.82 (dd, *J* = 7.7, 1.1 Hz, 1H), 7.75 – 7.70 (m, 1H), 7.35 – 7.26 (m, 4H), 7.26 – 7.19 (m, 2H), 7.19 – 7.15 (m, 1H), 6.78 (d, *J* = 8.5 Hz, 1H), 5.02 (s, 2H), 3.97 (s, 3H), 3.66 (s, 3H), 2.35 (s, 3H).

^{13}C NMR (101 MHz, chloroform-*d*) δ 182.1, 181.6, 164.0, 160.1, 154.9, 146.4, 138.1, 136.9, 135.4, 134.0, 131.6, 130.4, 128.5, 127.8, 126.9, 125.3, 123.9, 119.9, 119.4, 118.4, 111.2, 70.9, 56.6, 52.5, 22.5.

IR (Diamond-ATR, neat) $\tilde{\upsilon}_{max}$: 3032 (w), 3008 (w), 2951 (w), 2924 (w), 2844 (w), 1741 (m), 1665 (s), 1585 (m), 1471 (m), 1381 (w), 1349 (m), 1281 (s), 1237 (s), 1200 (w), 1159 (m), 1106 (w), 988 (m), 951 (w), 910 (w), 867 (w), 839 (w), 804 (w), 735 (w), 628 (w) cm^{-1}.

HRMS (ESI) calc. for $C_{27}H_{21}BrNaO_6$ $[M+Na]^+$: 543.0414; found: 543.0403.

Crystal structure: 5b underwent crystallization by dissolution in a minimal amount of acetonitrile followed by gradual solvent evaporation.
See chapter 3, page S50 for more details.

Naphthoquinone 13

TLC (50% ethyl acetate in cyclohexane): $R_f = 0.41$ (UV, $KMnO_4$).

mp: 189 − 191 °C.

^{1}H NMR (400 MHz, chloroform-*d*) δ 7.81 – 7.77 (m, 1H), 7.71 (t, *J* = 8.0 Hz, 1H), 7.36 – 7.33 (m, 1H), 7.31 – 7.26 (m, 4H), 7.25 – 7.21 (m, 1H), 7.20 – 7.17 (m, 1H), 6.81 (d, *J* = 8.5 Hz, 1H), 5.02 (q, *J* = 13.1 Hz, 2H), 4.03 (s, 3H), 3.67 (s, 3H), 2.36 (s, 3H).

^{13}C NMR (101 MHz, chloroform-*d*) δ 183.1, 181.1, 164.3, 160.1, 155.0, 142.2, 142.0, 136.8, 135.4, 134.1, 131.8, 130.6, 128.6, 127.8, 127.0, 125.5, 123.1, 119.9, 119.7, 118.3, 111.4, 71.0, 56.7, 52.5, 22.6.

IR (Diamond-ATR, neat) $\tilde{\upsilon}_{max}$: 2948 (w), 1741 (m), 1665 (m), 1585 (m), 1473 (m), 1381 (w), 1332 (w), 1273 (s), 1159 (w), 1122 (w), 1049 (w), 1014 (w), 986 (w), 943 (w), 873 (w), 806 (w), 741 (w) cm^{-1}.

HRMS (ESI) calc. for $C_{27}H_{21}BrNaO_6$ $[M+Na]^+$: 543.0414; found: 543.0410.

Dihydroxynaphthalene S6

TLC (50% ethyl acetate in cyclohexane): $R_f = 0.49$ (UV, $KMnO_4$).

mp: *Due to the fast oxidation of **S6** towards naphthoquinone **13**, no melting point could be measured.*

^{1}H NMR (400 MHz, chloroform-*d*) δ 10.88 (s, 1H), 7.89 (dd, *J* = 8.4, 0.9 Hz, 1H), 7.52 – 7.46 (m, 1H), 7.23 – 7.15 (m, 6H), 6.97 – 6.93 (m, 1H), 6.88 (d, *J* = 8.4 Hz, 1H), 5.01 (s, 2H), 4.80 (s, 1H), 4.08 (s, 3H), 3.50 (s, 3H), 2.40 (s, 3H).

^{13}C NMR (176 MHz, chloroform-*d*) δ 183.1*, 181.1*, 169.2, 164.3*, 160.1*, 157.7, 155.6, 155.0*, 151.4, 142.2*, 142.0*, 140.7, 136.9, 136.8*, 135.4*, 134.1*, 131.8*, 131.5, 131.1, 130.6*, 128.9, 128.6*, 128.6, 128.4, 128.1, 127.8*, 127.8, 127.0*, 126.9, 126.2, 125.5*, 123.1*, 119.9*, 119.7*, 118.3*, 117.4, 116.2, 116.0, 112.4, 111.4*, 111.3, 106.7, 71.0*, 70.9, 56.7*, 56.6, 52.5*, 51.9, 23.2, 22.6*.
*Note: The obtained mixture of two signal sets is arising from the fast oxidation of **S6** towards naphthoquinone **13**. All signals belonging to oxidized naphthoquinone **13** and therefore not to the product, are indicated with an asterisk**

IR (Diamond-ATR, neat) $\tilde{\upsilon}_{max}$: *Due to the fast oxidation of **S6** towards naphthoquinone **13**, no IR spectra was recorded.*

HRMS (ESI) calc. for $C_{27}H_{23}BrNaO_6$ $[M+Na]^+$: 545.0570; found: 545.0571.

Methyl 3-(6-(benzyloxy)-2-bromo-3-methylphenyl)-5-methoxy-1,4-dioxo-1,4-dihydronaphthalene-2-carboxylate 5b

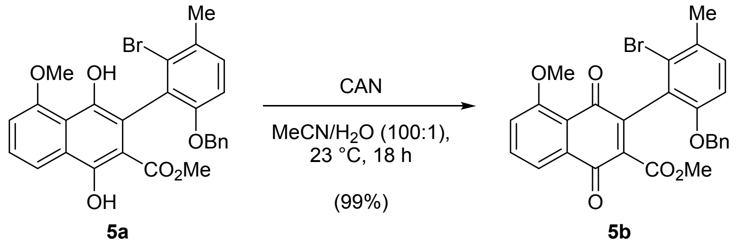

Ceric ammonium nitrate (1.43 g, 2.61 mmol, 1.20 equiv) was added to a solution of dihydroxynaphthalene **5a** (1.14 g, 2.18 mmol, 1 equiv) in acetonitrile (21.5 mL) and water (210 µL) at 23 °C. After 18 h, water (10 mL) was added, and the aqueous layer was extracted with ethyl acetate (3 × 8 mL). The combined organic layers were washed with a saturated aqueous solution of sodium chloride (10 mL). The washed solution was dried over magnesium sulfate, the dried solution was filtered and the filtrate was concentrated under reduced pressure. The residue was purified by flash column chromatography on silica gel (20% ethyl acetate in cyclohexane, grading to 30% ethyl acetate in cyclohexane) to yield naphthoquinone **5b** (1.13 g, 2.17 mmol, 99%) as an orange solid.

*For the analytical data of naphthoquinone **5b** see page S16.*

Methyl 3-(6-(benzyloxy)-2-(furan-2-yl)-3-methylphenyl)-5-methoxy-1,4-dioxo-1,4-dihydronaphthalene-2-carboxylate 15

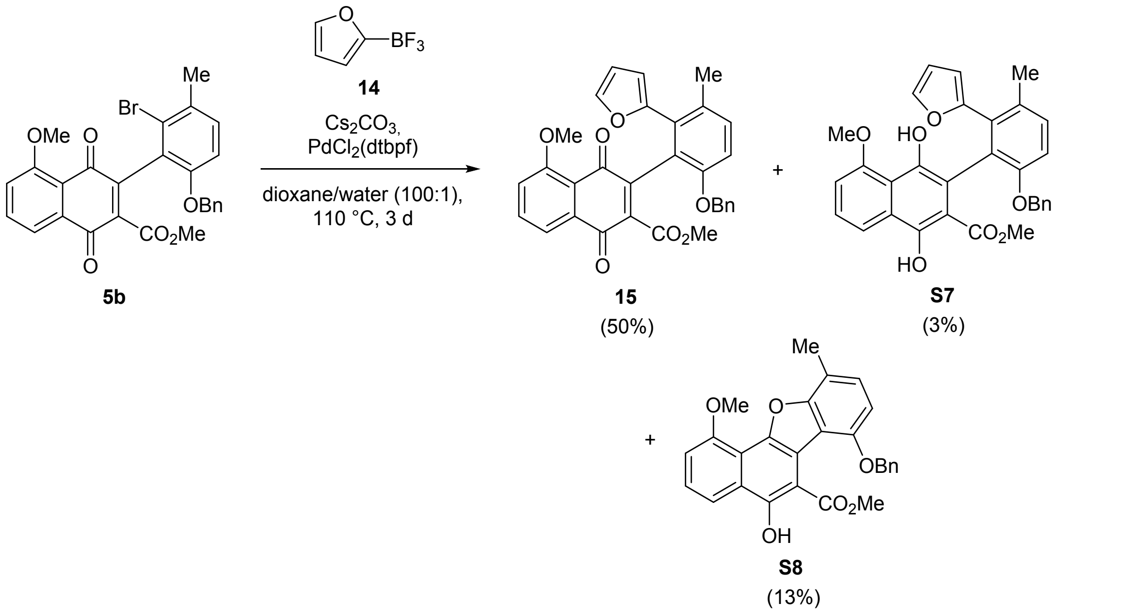

1,1'-Bis(di-*t*-butylphosphino)ferrocene palladium dichloride (10.0 mg, 15.3 µmol, 20.0 mol%) was added to a solution of cesium carbonate (75.0 mg, 230 µmol, 3.00 equiv), potassium trifluoro(furan-2-yl)borate **14** (26.7 mg, 153 µmol, 2.00 equiv) and bromo naphthoquinone **5b** (40.0 mg, 76.7 µmol, 1 equiv) in dioxane (1.2 mL) and water (3 drops) at 23 °C. Upon addition, the reaction was stirred at 110 °C. After 12 h, the reaction was allowed to cool down to 23 °C and additional 1,1'-bis(di-*t*-butylphosphino)ferrocene palladium dichloride (10.0 mg, 15.3 µmol, 20.0 mol%) and potassium trifluoro(furan-2-yl)borate **14** (13.4 mg, 76.5 µmol, 1.00 equiv) were added at 23 °C. Upon addition, the reaction was again stirred at 110 °C. After three days in total, the reaction was allowed to cool down to 23 °C and filtered through a pad of Celite. The pad was rinsed with ethyl acetate (10 mL) and the filtrate was concentrated under reduced pressure. The residue was purified by flash column chromatography on silica gel (10% ethyl acetate in cyclohexane, grading to 40% ethyl acetate in cyclohexane) to yield furan naphthoate **15** (19.5 mg, 38.3 µmol, 50%) as a red amorphous solid, furan dihydroxynaphthalene **S7** (1.3 mg, 2.5 µmol, 3%) as a yellow solid as well as the benzofuran side-product **S8** (4.4 mg, 9.9 µmol, 13%), which was obtained as a pale-brown solid.

Furan naphthoate 15

TLC (50% ethyl acetate in cyclohexane): R_f = 0.43 (UV, $KMnO_4$).

^{1}H NMR (400 MHz, chloroform-*d*) δ 7.74 (dd, *J* = 7.7, 1.2 Hz, 1H), 7.70 – 7.65 (m, 1H), 7.32 (dd, *J* = 1.8, 0.8 Hz, 1H), 7.31 – 7.27 (m, 3H), 7.24 – 7.17 (m, 4H), 6.84 (d, *J* = 8.5 Hz, 1H), 6.28 (dd, *J* = 3.3, 1.8 Hz, 1H), 6.25 (dd, *J* = 3.3, 0.8 Hz, 1H), 5.04 (s, 2H), 3.94 (s, 3H), 3.60 (s, 3H), 2.24 (s, 3H).

^{13}C NMR (101 MHz, chloroform-*d*) δ 182.8, 182.0, 164.1, 159.9, 154.1, 151.2, 148.0, 142.1, 137.2, 136.9, 135.1, 133.9, 132.2, 131.0, 130.1, 128.4, 127.6, 126.9, 122.7, 120.2, 119.2, 118.2, 112.5, 110.9, 110.7, 70.8, 56.6, 52.2, 20.2.

IR (Diamond-ATR, neat) $\tilde{\upsilon}_{max}$: 2951 (w), 1739 (m), 1665 (s), 1585 (s), 1471 (m), 1349 (m), 1281 (s), 1249 (s), 1196 (w), 1157 (m), 1104 (w), 1024 (m), 990 (m), 955 (w), 912 (w), 810 (w), 737 (m) cm^{-1}.

HRMS (ESI) calc. for $C_{31}H_{24}NaO_7$ $[M+Na]^+$: 531.1414; found: 531.1401.

Furan dihydroxynaphthalene S7

TLC (50% ethyl acetate in cyclohexane): R_f = 0.57 (UV, $KMnO_4$).

mp: 189 − 191 °C.

^{1}H NMR (400 MHz, chloroform-*d*) δ 11.74 (s, 1H), 9.06 (s, 1H), 8.05 (dd, *J* = 8.5, 1.0 Hz, 1H), 7.40 – 7.36 (m, 1H), 7.21 (dd, *J* = 1.9, 0.8 Hz, 1H), 7.18 – 7.11 (m, 6H), 7.00 – 6.91 (m, 2H), 6.10 (dd, *J* = 3.2, 1.8 Hz, 1H), 5.89 (dd, *J* = 3.3, 0.8 Hz, 1H), 5.03 (s, 2H), 3.99 (s, 3H), 3.45 (s, 3H), 2.21 (s, 3H).

^{13}C NMR (101 MHz, chloroform-*d*) δ 172.0, 155.7, 154.0, 153.5, 152.4, 143.6, 141.3, 138.0, 132.0, 130.7, 129.9, 129.4, 128.3, 127.4, 126.9, 126.8, 125.6, 118.1, 118.0, 116.5, 113.8, 110.3, 108.8, 108.3, 107.9, 70.9, 56.4, 51.9, 20.1.

IR (Diamond-ATR, neat) $\tilde{\upsilon}_{max}$: 3395 (w), 3067 (w), 3030 (w), 2948 (w), 2863 (w), 1734 (w), 1655 (m), 1604 (m), 1584 (w), 1453 (s), 1439 (s), 1390 (s), 1375 (s), 1318 (s), 1283 (s), 1254 (s), 1230 (s), 1161 (s), 1126 (m), 1063 (m), 1021 (m), 1000 (m), 955 (w), 912 (w), 881 (w), 792 (m), 733 (s), 698 (w), 598 (w), 456 (w) cm^{-1}.

HRMS (ESI) calc. for $C_{31}H_{26}NaO_7$ $[M+Na]^+$: 533.1571; found: 533.1560.

Benzofuran S8

TLC (50% ethyl acetate in cyclohexane): R_f = 0.64 (UV, $KMnO_4$).

mp: 117 − 119 °C.

^{1}H NMR (400 MHz, chloroform-*d*) δ 10.12 (s, 1H), 8.09 (dd, *J* = 8.4, 1.0 Hz, 1H), 7.52 (t, *J* = 8.1 Hz, 1H), 7.45 – 7.33 (m, 5H), 7.18 (dd, *J* = 8.1, 1.0 Hz, 1H), 7.17 – 7.14 (m, 1H), 6.79 (d, *J* = 8.1 Hz, 1H), 5.19 (s, 2H), 4.15 (s, 3H), 3.51 (s, 3H), 2.64 (s, 3H).

^{13}C NMR (101 MHz, chloroform-*d*) δ 171.0, 156.3, 155.3, 153.2, 152.5, 145.3, 136.9, 128.7, 128.3, 127.7, 127.4, 126.4, 125.7, 116.8, 116.3, 115.3, 114.9, 114.2, 109.6, 105.6, 104.4, 71.3, 56.6, 52.3, 14.7.

IR (Diamond-ATR, neat) $\tilde{\upsilon}_{max}$: 2951 (w), 1669 (m), 1581 (m), 1510 (s), 1457 (m), 1339 (m), 1267 (s), 1247 (m), 1214 (m), 1163 (m), 1094 (m), 1061 (m), 1002 (m), 965 (w), 910 (w), 841 (w), 784 (m), 737 (m), 696 (w) cm^{-1}.

HRMS (ESI) calc. for $C_{27}H_{22}NaO_6$ $[M+Na]^+$: 465.1309; found: 465.1301.

Methyl 3-(6-(benzyloxy)-2-(furan-2-yl)-3-methylphenyl)-5-methoxy-1,4-dioxo-1,4-dihydronaphthalene-2-carboxylate 15

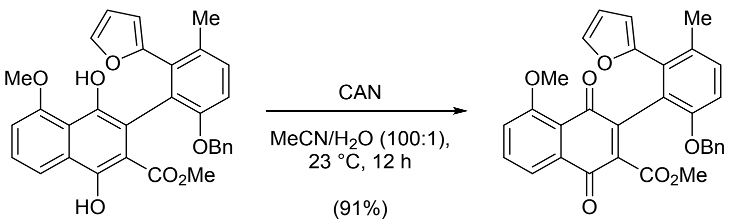

S7 **15**

Ceric ammonium nitrate (27.7 mg, 50.5 µmol, 1.20 equiv) was added to a solution of furan dihydroxynaphthalene **S7** (21.5 mg, 42.1 µmol, 1 equiv) in acetonitrile (500 µL) and water (1 drop) at 23 °C. After 12 h, water (1 mL) was added, and the aqueous layer was extracted with ethyl acetate (3 × 2 mL). The combined organic layers were washed with a saturated aqueous solution of sodium chloride (3 mL). The washed solution was dried over magnesium sulfate, the dried solution was filtered and the filtrate was concentrated under reduced pressure. The residue was purified by flash column chromatography on silica gel (10% ethyl acetate in cyclohexane, grading to 20% ethyl acetate in cyclohexane) to yield furan naphthoquinone **15** (19.5 mg, 38.3 µmol, 91%) a red amorphous solid.

*For the analytical data of furan naphthoquinone **15** see page S19.*

3-(Benzyloxy)-2-(8-methoxy-3-(methoxycarbonyl)-1,4-dioxo-1,4-dihydronaphthalen-2-yl)-6-methylbenzoic acid 16

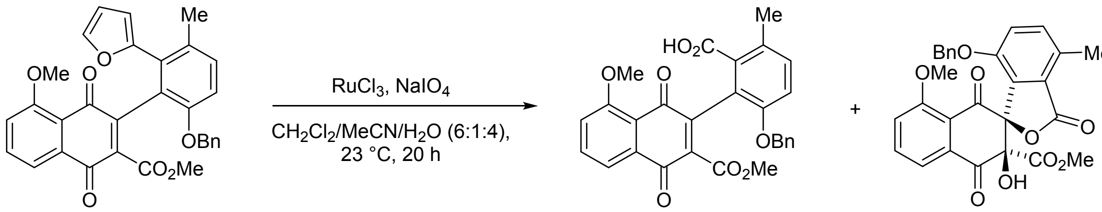

15 **16** (24–58%) **17a** (2–20%)* *as mixture with **16**

Ruthenium trichloride (3.73 mg, 18.0 µmol, 5.00 mol%) and sodium periodate (616 mg, 2.88 mmol, 8.00 equiv) were dissolved in dichloromethane (1.44 mL), acetonitrile (480 µL) and water (1.92 mL) at 23 °C. After one hour, a solution of furan naphthoquinone **15** (183 mg, 360 µmol, 1 equiv) in dichloromethane (1.44 mL) was added dropwise at 23 °C. The reaction was stirred for 20 h at 23 °C and then water (5 mL) was added. The aqueous layer was extracted with dichloromethane (3 × 6 mL) and the combined organic layers were washed with a saturated aqueous solution of sodium bicarbonate (8 mL). The washed solution was dried over magnesium sulfate, the dried solution was filtered and the filtrate was concentrated under reduced pressure. The residue was purified by flash column chromatography on silica gel (20% ethyl acetate in cyclohexane, grading to 60% ethyl acetate in cyclohexane) to yield carboxylic acid **16** (102 mg, 210 µmol, 58%) as pale-yellow amorphous solid, accompanied by an inseparable mixture of carboxylic acid **16** with spiro-lactone **17a** (~3.52 mg, 7.24 µmol, 2%).

Carboxylic acid 16

TLC (50% ethyl acetate in cyclohexane): $R_f = 0.33$ (UV, CAM, $KMnO_4$).

^{1}H NMR (400 MHz, chloroform-*d*) δ 13.46 (s, 1H), 7.61 – 7.51 (m, 2H), 7.23 – 7.13 (m, 4H), 7.06 (dd, *J* = 8.1, 1.5 Hz, 1H), 6.91 – 6.87 (m, 3H), 4.82 (d, *J* = 11.4 Hz, 1H), 4.71 (d, *J* = 11.5 Hz, 1H), 3.89 (s, 3H), 3.53 (s, 3H), 2.64 (s, 3H).

^{13}C NMR (101 MHz, chloroform-*d*) δ 187.5, 171.6, 170.8, 165.6, 160.4, 150.6, 137.1, 136.2, 135.7, 134.8, 132.7, 130.7, 128.6, 127.9, 127.3, 126.4, 118.4, 118.0, 116.4, 115.6, 97.6, 70.1, 56.4, 52.4, 16.5.

IR (Diamond-ATR, neat) $\tilde{\upsilon}_{max}$: 3029 (w), 2955 (w), 2872 (w), 1765 (s), 1693 (m), 1654 (m), 1623 (m), 1579 (s), 1503 (m), 1464 (m), 1363 (m), 1301 (m), 1272 (s), 1223 (s), 1181 (m), 1138 (w), 1037 (w), 977 (m), 911 (w), 825 (w), 798 (w), 730 (w) cm^{-1}.

HRMS (ESI) calc. for $C_{28}H_{22}NaO_8$ $[M+Na]^+$: 509.1207; found: 509.1200.

Methyl (1*S*,3'*R*)-7-(benzyloxy)-3'-hydroxy-8'-methoxy-4-methyl-1',3,4'-trioxo-3',4'-dihydro-1'*H*,3*H*-spiro[isobenzofuran-1,2'-naphthalene]-3'-carboxylate 17a

Methyl (1*R*,3'*R*)-7-(benzyloxy)-3'-hydroxy-8'-methoxy-4-methyl-1',3,4'-trioxo-3',4'-dihydro-1'*H*,3*H*-spiro[isobenzofuran-1,2'-naphthalene]-3'-carboxylate 17b

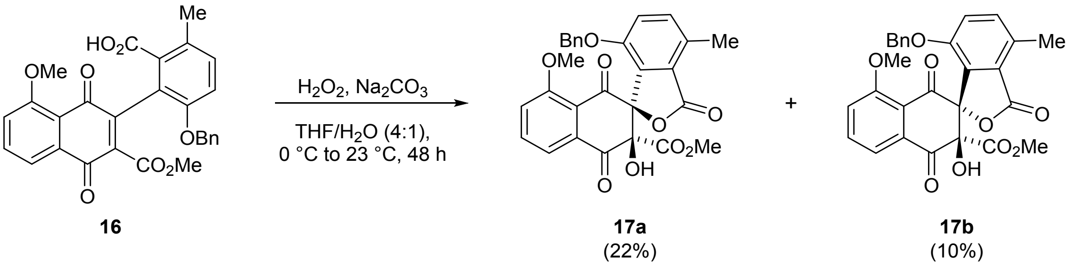

A solution of hydrogen peroxide (30wt% in water, 8.46 µL, 82.9 µmol, 1.20 equiv) was added dropwise to a solution of carboxylic acid **16** (33.6 mg, 69.1 µmol, 1 equiv) in tetrahydrofuran (5 mL) at 0 °C. After five minutes, a solution of sodium carbonate (8.78 mg, 82.9 µmol, 1.20 equiv) in water (1.25 mL) was added dropwise at 0 °C. Upon addition, the reaction was allowed to warm up to 23 °C overnight. After 48 h, water (4 mL) was added and the aqueous layer was extracted with ethyl acetate (3 × 5 mL). The combined organic layers were washed with a saturated aqueous solution of sodium bicarbonate (10 mL). The washed solution was dried over magnesium sulfate, the dried solution was filtered and the filtrate was concentrated under reduced pressure. The residue was purified by preparative HPLC (20% ethyl acetate in *n*-hexane, grading to 50% ethyl acetate in *n*-hexane in 30 min) to yield separated spiro-lactone **17a** (7.74 mg, 15.4 µmol, 22%) as a pale-yellow amorphous solid and spiro-lactone **17b** (3.41 mg, 6.79 µmol, 10%) as a colorless amorphous solid.

Spiro-lactone 17a

TLC (50% ethyl acetate in cyclohexane): R_f = 0.32 (UV, CAM).

^{1}H NMR (400 MHz, chloroform-*d*) δ 7.83 (dd, *J* = 7.7, 1.1 Hz, 1H), 7.74 – 7.69 (m, 1H), 7.45 – 7.41 (m, 2H), 7.40 – 7.34 (m, 3H), 7.32 – 7.27 (m, 1H), 7.25 – 7.22 (m, 1H), 7.06 (d, *J* = 8.4 Hz, 1H), 5.20 (d, *J* = 12.9 Hz, 1H), 5.11 (d, *J* = 12.9 Hz, 1H), 4.36 (s, 1H), 3.99 (s, 3H), 3.26 (s, 3H), 2.55 (d, *J* = 0.8 Hz, 3H).

^{13}C NMR (101 MHz, chloroform-*d*) δ 190.5, 183.1, 167.7, 166.2, 160.1, 153.1, 136.2, 135.0, 134.3, 132.1, 130.4, 128.8, 128.5, 128.3, 127.6, 126.1, 124.7, 120.5, 119.2, 118.9, 92.3, 83.7, 72.0, 56.8, 53.6, 16.7.

IR (Diamond-ATR, neat) $\tilde{\upsilon}_{max}$: 3408 (w), 3308 (w), 2924 (s), 2859 (m), 1724 (w), 1455 (m), 1375 (m), 1330 (w), 1306 (w), 1235 (w), 1153 (m), 1084 (w), 981 (w), 910 (w), 851 (w), 743 (w), 631 (m), 565 (w), 500 (w), 465 (w) cm^{-1}.

HRMS (ESI) calc. for $C_{28}H_{22}NaO_9$ $[M+Na]^+$: 525.1156; found: 525.1149.

Crystal structure: 17a crystallized by gradual solvent evaporation from a solution of deuterated chloroform, which was overlayed with diethyl ether.
See chapter 3, page S51 for more details.

Spiro-lactone 17b

TLC (50% ethyl acetate in cyclohexane): $R_f = 0.22$ (UV, CAM).

^{1}H NMR (700 MHz, chloroform-*d*) δ 7.51 – 7.47 (m, 1H), 7.46 – 7.44 (m, 1H), 7.28 – 7.21 (m, 4H), 7.11 (d, J = 8.2 Hz, 1H), 6.86 (d, J = 7.4 Hz, 2H), 6.77 (d, J = 8.3 Hz, 1H), 4.69 (d, J = 12.4 Hz, 1H), 4.60 (d, J = 12.4 Hz, 1H), 4.44 (s, 1H), 3.96 (s, 3H), 3.75 (s, 3H), 2.57 (s, 3H).

^{13}C NMR (176 MHz, chloroform-*d*) δ 189.5, 184.3, 168.6, 167.3, 160.5, 150.8, 136.6, 135.6, 135.0, 133.8, 132.2, 131.6, 128.7, 128.3, 127.6, 125.6, 122.4, 119.3, 118.0, 117.0, 90.9, 83.6, 70.1, 56.7, 54.6, 16.6.

IR (Diamond-ATR, neat) $\tilde{\upsilon}_{max}$: 3460 (w), 3375 (w), 2924 (m), 2852 (w), 1773 (s), 1701 (s), 1621 (w), 1588 (m), 1503 (s), 1470 (m), 1379 (m), 1274 (s), 1231 (s), 1190 (s), 1019 (s), 961 (m), 889 (m), 825 (m), 724 (s), 647 (m), 600 (m), 540 (w), 499 (w), 429 (m) cm^{-1}.

HRMS (ESI) calc. for $C_{28}H_{22}NaO_9$ $[M+Na]^+$: 525.1156; found: 525.1148.

7-*Epi*-chartspiroton 18

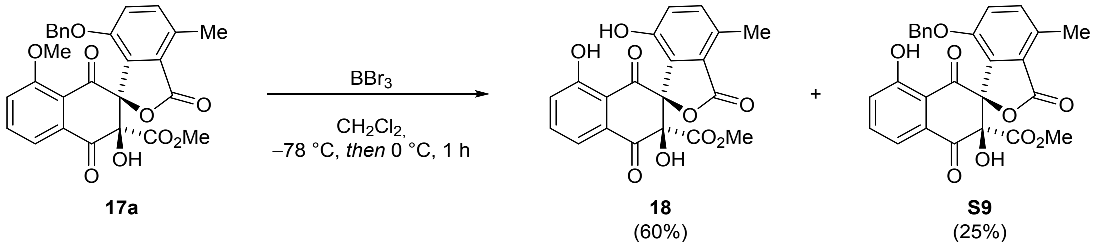

A solution of boron tribromide (1.00 M in dichloromethane, 8.96 µL, 8.96 µmol, 1.00 equiv) was added to a solution of spiro-lactone **17a** (4.50 mg, 8.96 µmol, 1 equiv) in dichloromethane (200 µL) at −78 °C. Upon addition, the reaction was stirred at 0 °C. After one hour, the ice bath was removed and water (1 mL) was added. The layers were separated and the aqueous layer was extracted with dichloromethane (3 × 2 mL). The combined organic layers were washed with a saturated aqueous solution of sodium bicarbonate (4 mL). The washed solution was dried over magnesium sulfate, the dried solution was filtered and the filtrate was concentrated under reduced pressure. The residue was purified by preparative HPLC (10% ethyl acetate in *n*-hexane, grading to 60% ethyl acetate in *n*-hexane in 30 min) to yield

separated 7-*epi*-chartspiroton **18** (2.14 mg, 5.37 µmol, 60%) as a colorless amorphous solid along with benzyl ether **S9** (1.10 mg, 2.25 µmol, 25%), which was obtained as a pale-yellow oil.

7-*Epi*-chartspiroton 18

*7-Epi-chartspiroton **18** was found to start converting into chartspiroton (**1**) during the recording of the NMR spectra in deuterated dimethyl sulfoxide. Due to the fast conversion, no IR spectrum for 7-epi-chartspiroton **18** was measured and proton and carbon NMR spectra of 7-epi-chartspiroton **18** show signals of chartspiroton (**1**) as well.*

TLC (50% ethyl acetate in cyclohexane): R_f = 0.17 (UV, CAM, $KMnO_4$).

^{1}H NMR (400 MHz, DMSO-d_6) δ 10.93 (s, 1H), 10.40 (s, 1H), 7.86 – 7.81 (m, 1H), 7.65 (dd, J = 7.5, 1.1 Hz, 1H), 7.42 (dd, J = 8.5, 1.1 Hz, 1H), 7.30 – 7.26 (m, 1H), 7.02 – 6.98 (m, 1H), 3.56 (s, 3H), 2.46 (s, 3H).

^{13}C NMR (101 MHz, DMSO-d_6) δ 192.1*, 188.8*, 168.7*, 167.6*, 166.3, 160.4*, 160.1, 151.1, 150.1*, 137.4*, 137.0, 135.5*, 133.9*, 133.8, 129.5*, 128.9*, 128.6, 124.3, 124.1*, 123.9*, 121.3, 121.2*, 119.1, 118.5*, 117.6*, 90.2*, 84.5*, 83.3, 53.8*, 53.1, 15.8*.

*Signals, which belong to chartspiroton (**1**) as well, are indicated with an asterisk.**

HRMS (ESI) calc. for $C_{20}H_{14}NaO_9$ $[M+Na]^+$: 421.0530; found: 421.0514.

Benzyl ether S9

TLC (50% ethyl acetate in cyclohexane): R_f = 0.45 (UV, CAM, $KMnO_4$).

^{1}H NMR (700 MHz, chloroform-d) δ 11.15 (s, 1H), 7.70 – 7.65 (m, 2H), 7.39 – 7.28 (m, 7H), 7.09 (d, J = 8.4 Hz, 1H), 5.17 – 5.04 (m, 2H), 4.44 (s, 1H), 3.26 (s, 3H), 2.58 (s, 3H).

^{13}C NMR (176 MHz, chloroform-d) δ 191.3, 189.1, 167.3, 166.2, 161.8, 152.5, 136.8, 135.6, 134.7, 133.2, 132.3, 129.6, 128.9, 128.5, 127.7, 126.1, 125.1, 120.3, 118.7, 90.2, 83.5, 71.6, 53.6, 16.6.

IR (Diamond-ATR, neat) $\tilde{\upsilon}_{max}$: 3450 (w), 2959 (w), 2930 (w), 1779 (s), 1709 (m), 1666 (m), 1604 (w), 1503 (s), 1456 (m), 1381 (w), 1353 (w), 1250 (s), 1190 (s), 1151 (m), 1105 (w), 986 (m), 911 (w), 862 (w), 829 (m), 786 (w), 740 (m), 697 (w), 571 (w), 513 (w), 464 (w) cm^{-1}.

HRMS (ESI) calc. for $C_{27}H_{20}NaO_9$ $[M+Na]^+$: 511.1000; found: 511.0984.

Chartspiroton (1)

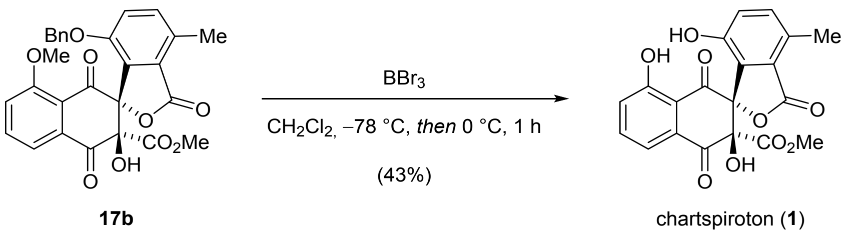

A solution of boron tribromide (1.00 M in dichloromethane, 11.3 µL, 11.3 µmol, 1.00 equiv) was added to a solution of spiro-lactone **17b** (5.70 mg, 11.3 µmol, 1 equiv) in dichloromethane (200 µL) at −78 °C. Upon addition, the reaction was stirred at 0 °C. After one hour, the ice bath was removed and water (1 mL) was added. The layers were separated and the aqueous layer was extracted with dichloromethane (3 × 2 mL). The combined organic layers were washed with a saturated aqueous solution of sodium bicarbonate (4 mL). The washed solution was dried over magnesium sulfate, the dried solution was filtered and the filtrate was concentrated under reduced pressure. The residue was purified by preparative HPLC (40% ethyl acetate in *n*-hexane, grading to 60% ethyl acetate in *n*-hexane in 30 min) to yield chartspiroton (**1**) (1.95 mg, 4.90 µmol, 43%) as a pale-yellow amorphous solid.

TLC (50% ethyl acetate in cyclohexane): R_f = 0.22 (UV, CAM, $KMnO_4$).

^{1}H NMR (700 MHz, DMSO-d_6) δ 11.22 (s, 1H), 10.39 (s, 1H), 7.83 (*t*, J = 7.9 Hz, 1H), 7.65 (d, *J* = 7.4 Hz, 1H), 7.40 (d, *J* = 8.3 Hz, 1H), 7.34 (s, 1H), 7.22 (d, *J* = 8.0 Hz, 1H), 6.80 (d, *J* = 8.0 Hz, 1H), 3.66 (s, 3H), 2.47 (s, 3H).

^{13}C NMR (176 MHz, DMSO-d_6) δ 192.5, 189.3, 169.1, 168.1, 160.9, 150.5, 137.9, 135.9, 134.4, 130.0, 129.4, 124.6, 124.4, 121.6, 118.9, 118.1, 90.6, 85.0, 54.3, 16.2.

IR (Diamond-ATR, neat) $\tilde{\upsilon}_{max}$: 3390 (w), 3078 (w), 3037 (w), 2957 (w), 2926 (w), 2856 (w), 2724 (w), 2666 (w), 1773 (s), 1709 (m), 1660 (m), 1606 (w), 1577 (w), 1505 (m), 1456 (m), 1316 (m), 1223 (s), 1165 (m), 1097 (w), 1023 (m), 901 (w), 831 (m), 777 (w), 734 (m) cm^{-1}.

HRMS (ESI) calc. for $C_{20}H_{14}NaO_9$ $[M+Na]^+$: 421.0530; found: 421.0519.

^{1}H NMR comparison for chartspiroton (1)

**Natural**[11] (500 MHz, DMSO-d_6) δ_H [ppm]	**Synthetic** (700 MHz, DMSO-d_6) δ_H [ppm]	$\Delta\delta_H$* [ppm]
2.48 (s, 3H)	2.47 (s, 3H)	+0.01
3.67 (s, 3H)	3.66 (s, 3H)	+0.01
6.81 (d, J = 8.15 Hz, 1H)	6.80 (d, J = 8.0 Hz, 1H)	+0.01
7.22 (d, J = 8.15 Hz, 1H)	7.22 (d, J = 8.0 Hz, 1H)	0
7.37 (s, 1H)	7.34 (s, 1H)	+0.03
7.40 (d, J = 8.40 Hz, 1H)	7.40 (d, J = 8.3 Hz, 1H)	0
7.66 (d, J = 7.35 Hz, 1H)	7.65 (d, J = 7.4 Hz, 1H)	+0.01
7.84 (brt, J = 8.40, 7.35 Hz, 1H)	7.83 (t, J = 7.9 Hz, 1H)	+0.01
10.44 (s, 1H)	10.39 (s, 1H)	+0.05
11.23 (s, 1H)	11.22 (s, 1H)	+0.01

^{13}C NMR comparison for chartspiroton (1)

**Natural**[11] (125 MHz, DMSO-d_6) δ_C [ppm]	**Synthetic** (176 MHz, DMSO-d_6) δ_C [ppm]	$\Delta\delta_C$* [ppm]
16.23	16.23	0
54.25	54.26	-0,01
84.95	84.96	-0,01
90.65	90.65	0
118.12	118.12	0
118.92	118.92	0
121.63	121.62	+0,01
124.41	124.42	-0,01
124.54	124.55	-0,01
129.31	129.36	-0,05
129.99	130.00	-0,01
134.33	134.35	-0,02
135.93	135.94	-0,01
137.90	137.91	-0,01
150.60	150.52	+0,08
160.91	160.91	0
168.06	168.06	0
169.12	169.12	0
189.28	189.29	-0,01
192.52	192.53	-0,01

*compared to the cited reference.[11]

[11] H. Zhao, A. Yang, J. Liu, S. Bao, R. Peng, Y. Hu, T. Yuan, S. Hou, T. Xie, Q. Zhang, X. Chen, X. Wang, L. Hu, *Org. Lett.* **2020**, *22* (10), 3739–3743.

2.2. Overview of Failed Routes to the Biaryl

Failed Route 1: Conjugated Addition

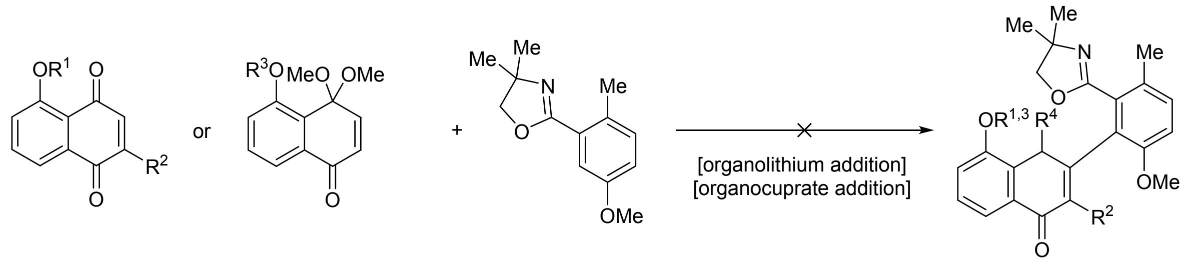

R^1 = Me, Ac, MOM
R^2 = H, Br

R^3 = Me, TBS, Bz

R^4 = O or $(OMe)_2$

Tetrahedron Lett. **1989**, *30*, 38, 5093–5096.

- No desired product
- No reactivity, decomposition or complex reaction mixtures observed

Failed Route 2: Heck-Coupling

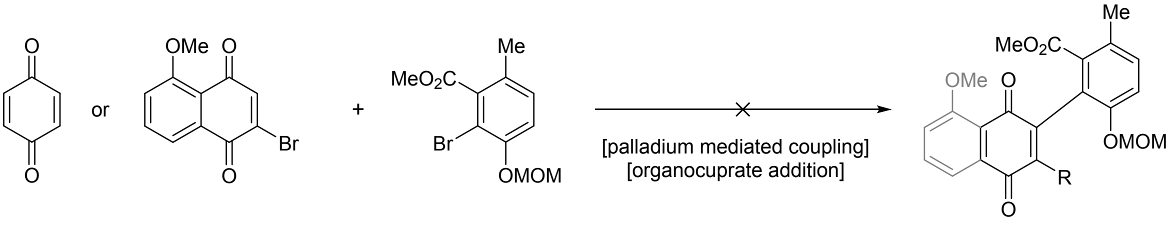

R = H, Br

- No desired product
- No reactivity or complex reaction mixtures observed

Failed Route 3: Diels–Alder Cycloaddition

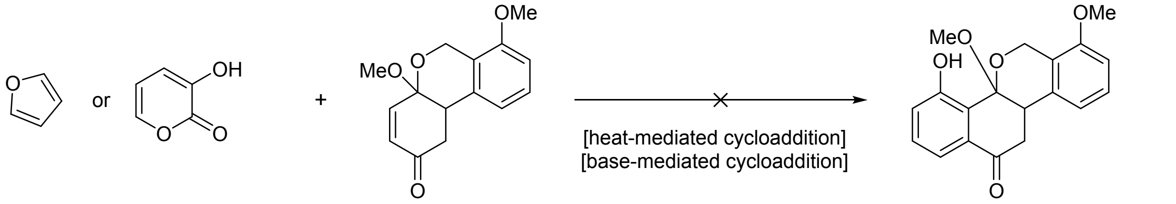

- No desired product
- No reactivity or complex reaction mixtures observed

2.3. Spectroscopic Data

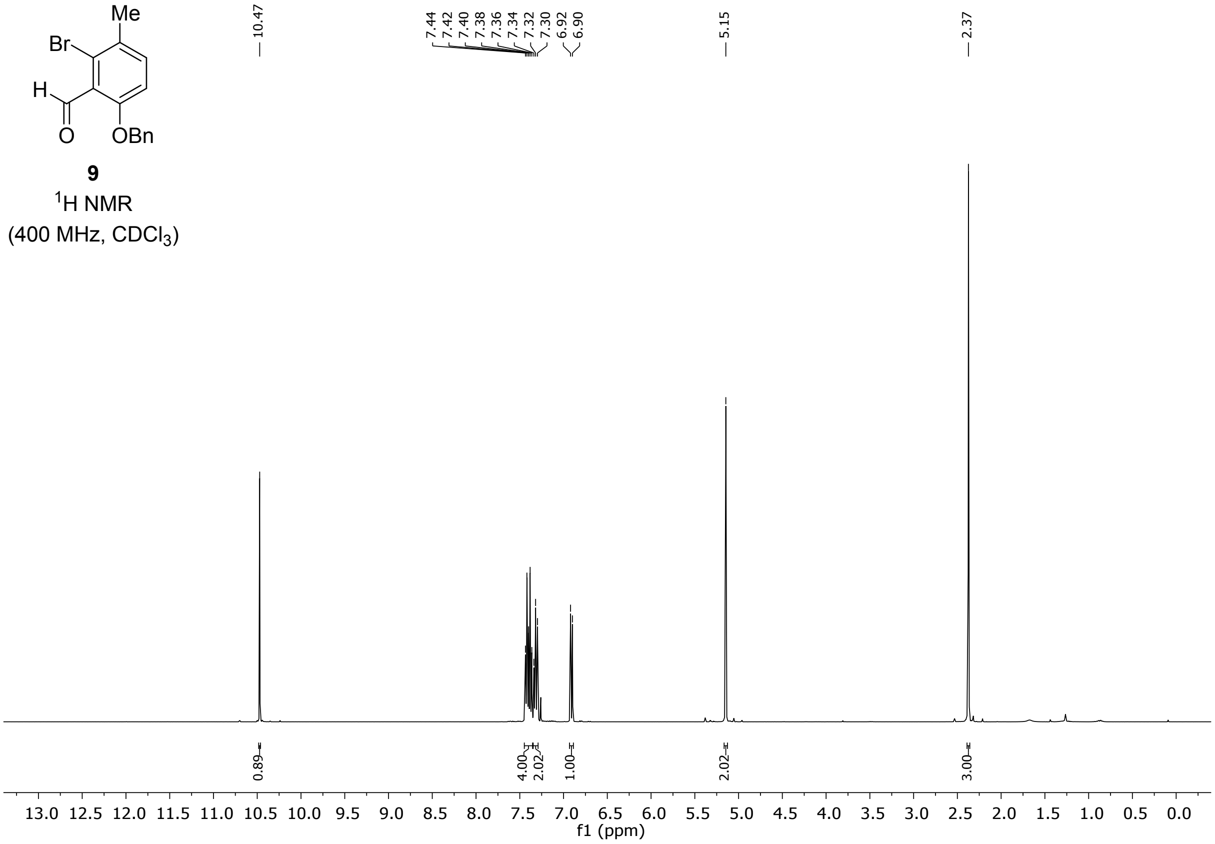

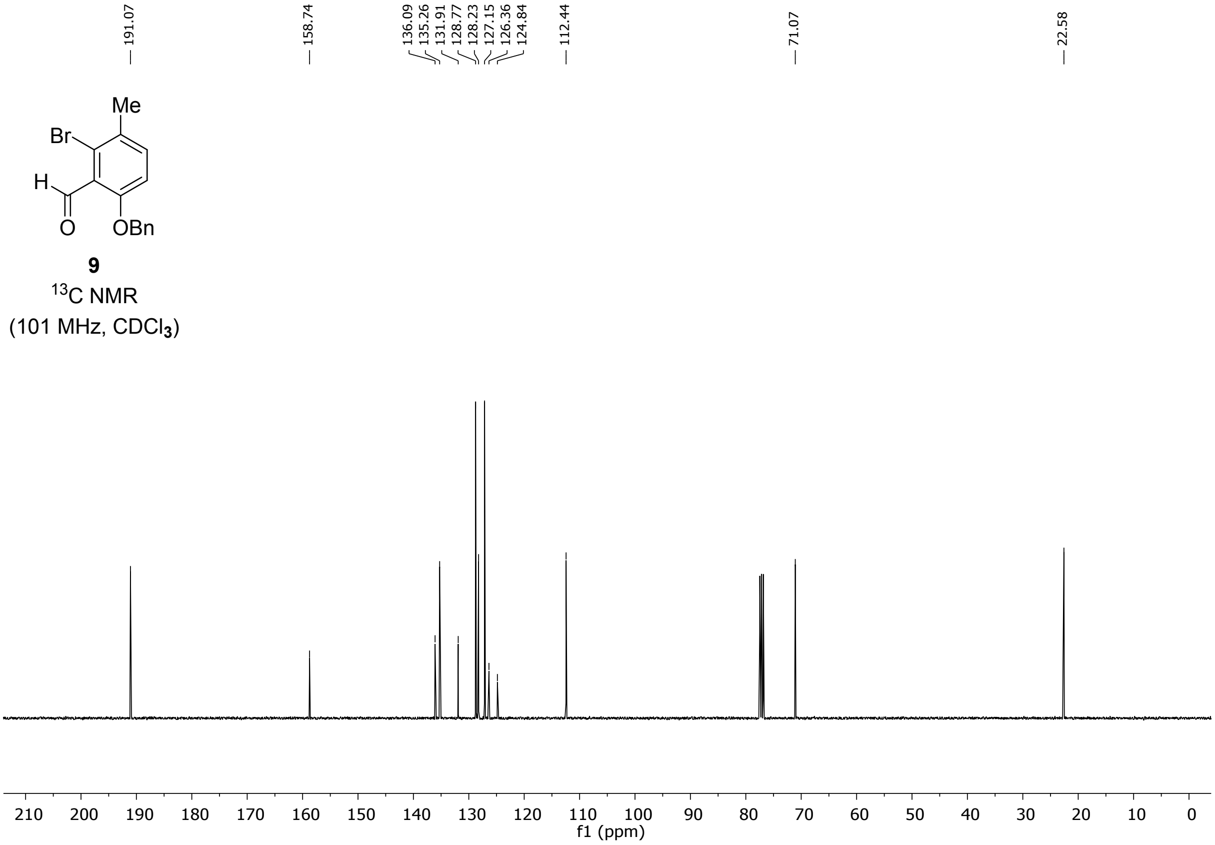

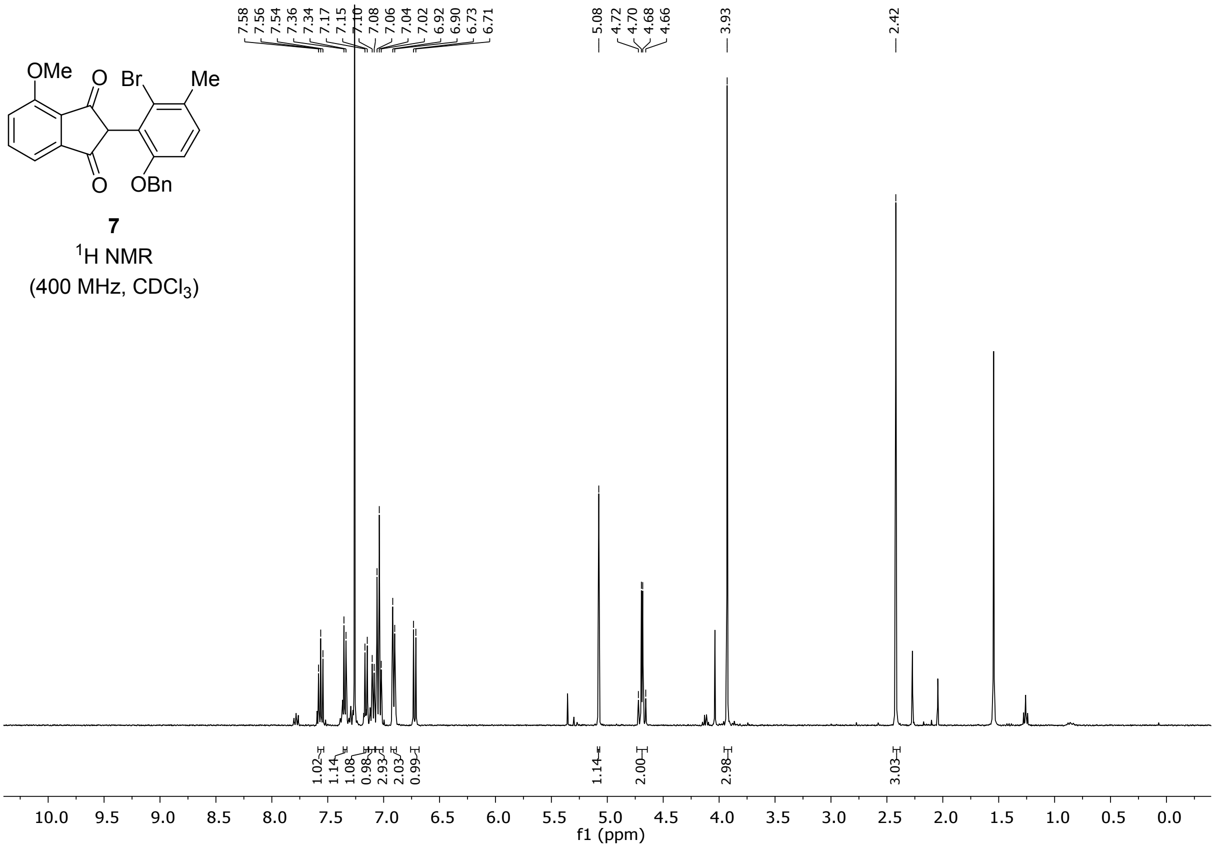
OMe
O
Br
Me
O
OBn
7
^{1}H NMR
(400 MHz, CDCl$_3$)
7.58 7.56 7.54 7.36 7.34 7.17 7.15 7.10 7.08 7.06 7.04 7.02 6.92 6.90 6.73 6.71
5.08 4.72 4.70 4.68 4.66
3.93
2.42
1.02 1.14 1.08 0.98 2.93 2.03 0.99 1.14 2.00 2.98 3.03
10.0 9.5 9.0 8.5 8.0 7.5 7.0 6.5 6.0 5.5 5.0 4.5 4.0 3.5 3.0 2.5 2.0 1.5 1.0 0.5 0.0
f1 (ppm)

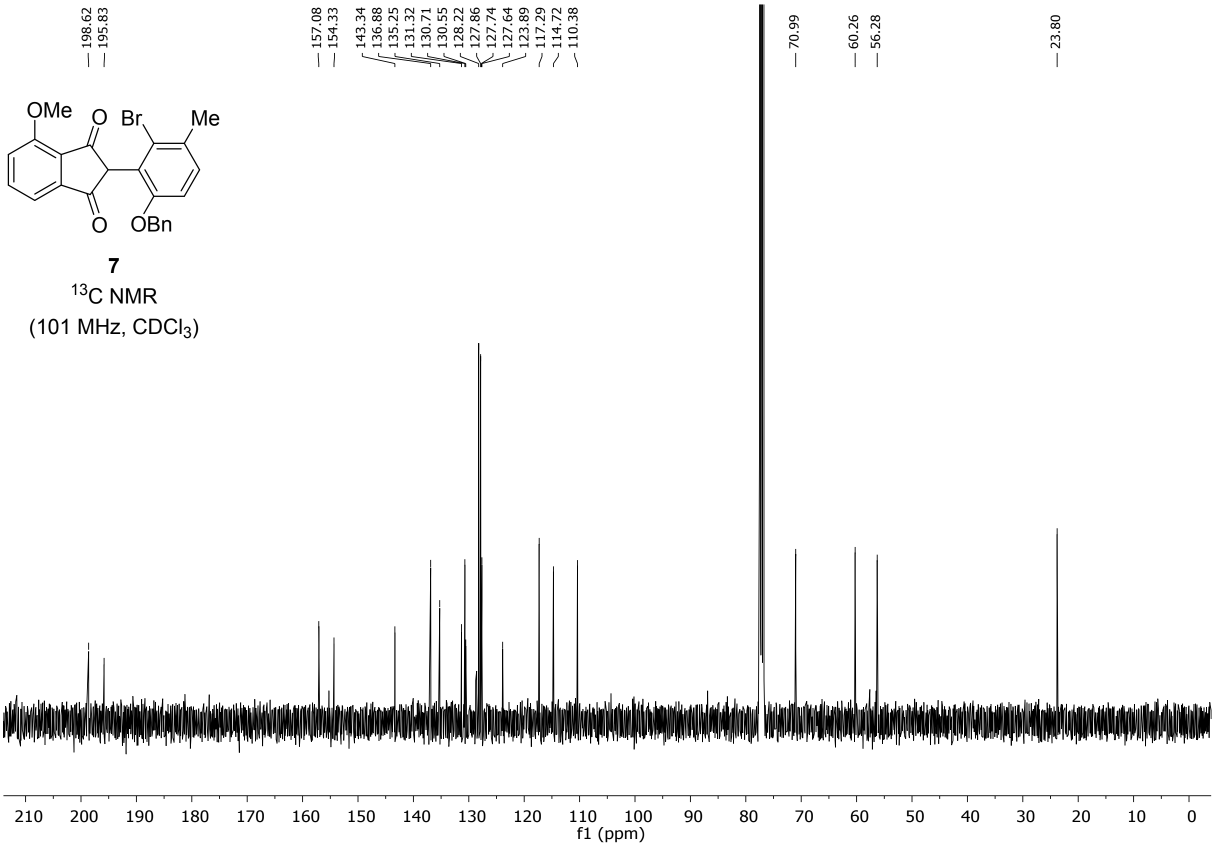
198.62 195.83
157.08 154.33
143.34 136.88 135.25 131.32 130.71 130.55 128.22 127.86 127.74 127.64 123.89 117.29 114.72 110.38
70.99
60.26 56.28
23.80
OMe
O
Br
Me
O
OBn
7
^{13}C NMR
(101 MHz, CDCl$_3$)
210 200 190 180 170 160 150 140 130 120 110 100 90 80 70 60 50 40 30 20 10 0
f1 (ppm)

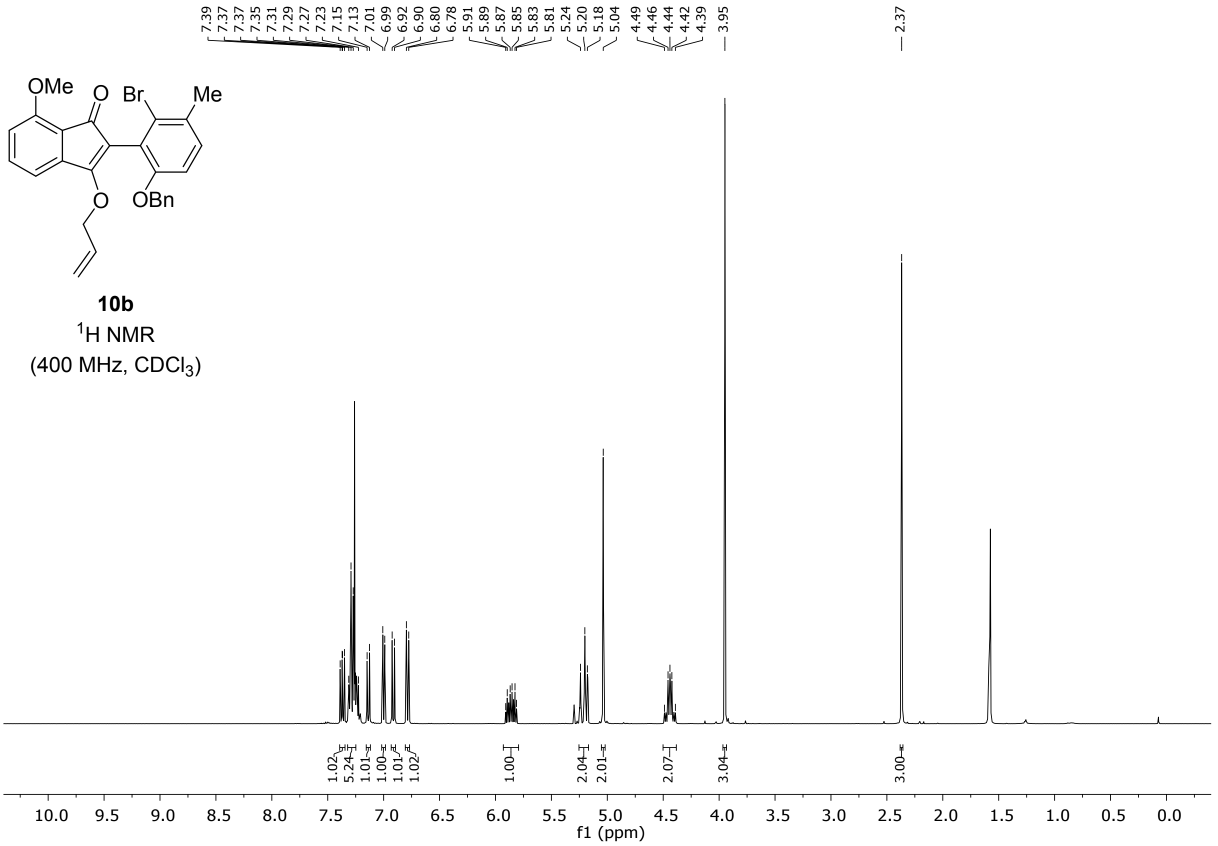
7.39 7.37 7.37 7.35 7.31 7.29 7.27 7.23 7.15 7.13 7.01 6.99 6.92 6.90 6.80 6.78 5.91 5.89 5.87 5.85 5.83 5.81 5.24 5.20 5.18 5.04 4.49 4.46 4.44 4.42 4.39 3.95 2.37
OMe
O
Br
Me
O
OBn
10b
^{1}H NMR
(400 MHz, $CDCl_3$)
1.02 5.24 1.01 1.00 1.01 1.02 1.00 2.04 2.01 2.07 3.04 3.00
10.0 9.5 9.0 8.5 8.0 7.5 7.0 6.5 6.0 5.5 5.0 4.5 4.0 3.5 3.0 2.5 2.0 1.5 1.0 0.5 0.0
f1 (ppm)

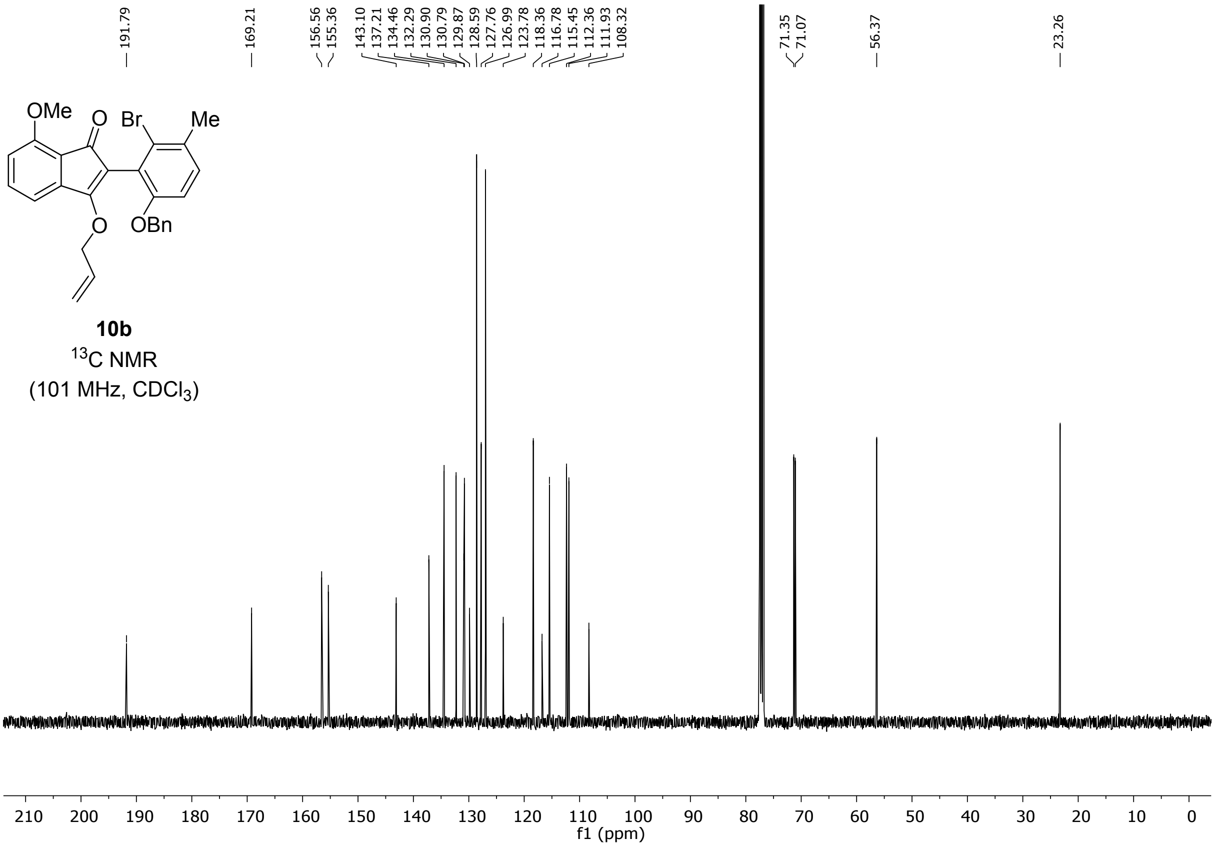
191.79 169.21 156.56 155.36 143.10 137.21 134.46 132.29 130.90 130.79 129.87 128.59 127.76 126.99 123.78 118.36 116.78 115.45 112.36 111.93 108.32 71.35 71.07 56.37 23.26
OMe
O
Br
Me
O
OBn
10b
^{13}C NMR
(101 MHz, $CDCl_3$)
210 200 190 180 170 160 150 140 130 120 110 100 90 80 70 60 50 40 30 20 10 0
f1 (ppm)

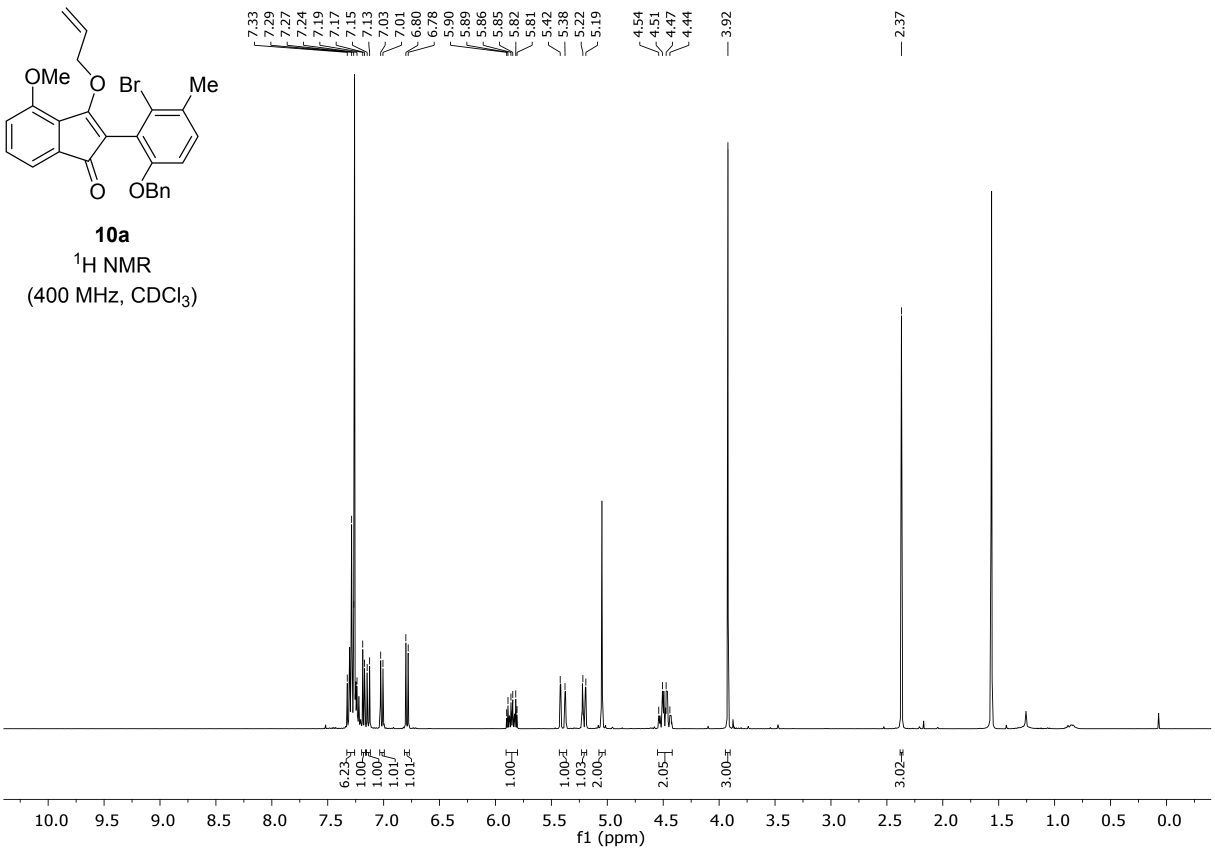
OMe
O
Br
Me
O
OBn
10a
^{1}H NMR
(400 MHz, CDCl$_3$)
7.33 7.29 7.27 7.24 7.19 7.17 7.15 7.13 7.03 7.01 6.80 6.78 5.90 5.89 5.86 5.85 5.82 5.81 5.42 5.38 5.22 5.19 4.54 4.51 4.47 4.44 3.92 2.37
6.23 1.00 1.00 1.01 1.01 1.00 1.00 1.03 2.00 2.05 3.00 3.02
10.0 9.5 9.0 8.5 8.0 7.5 7.0 6.5 6.0 5.5 5.0 4.5 4.0 3.5 3.0 2.5 2.0 1.5 1.0 0.5 0.0
f1 (ppm)

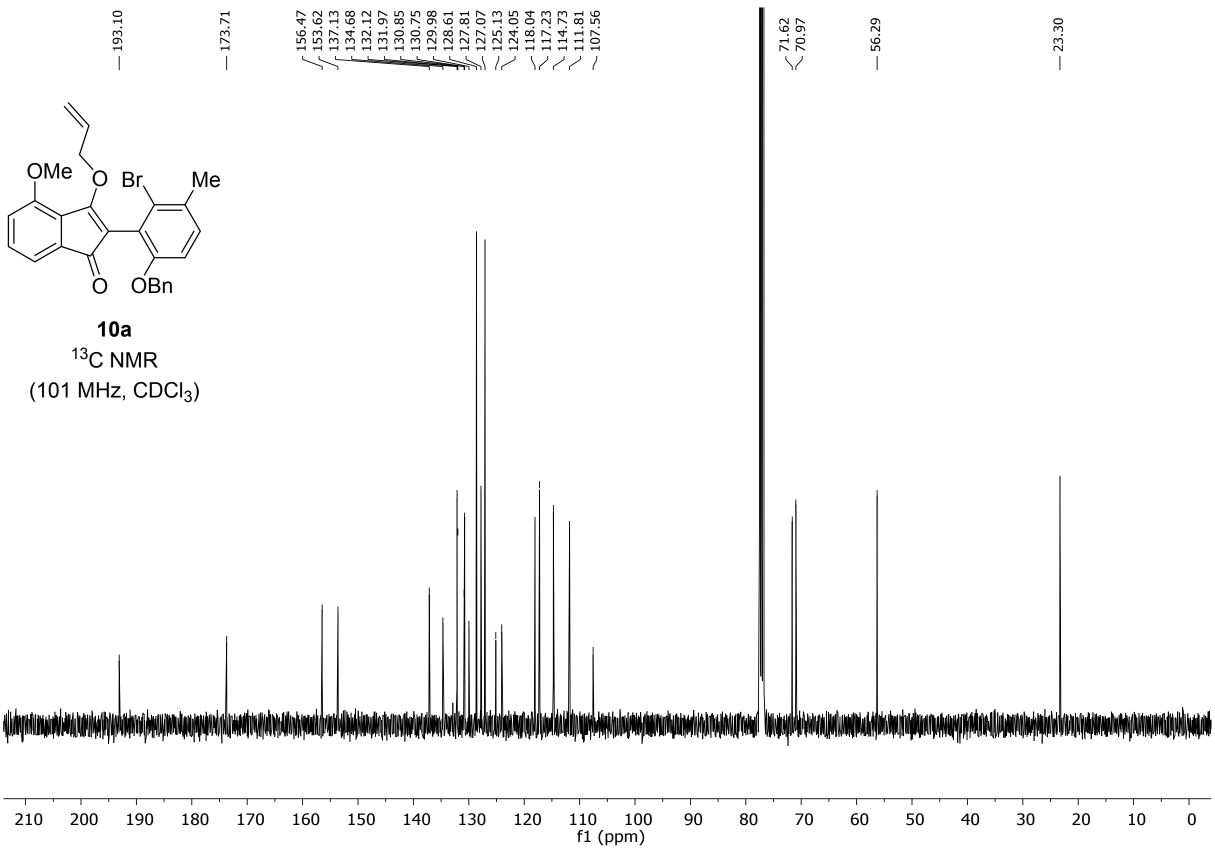
193.10 173.71 156.47 153.62 137.13 134.68 132.12 131.97 130.85 130.75 129.98 128.61 127.81 127.07 125.13 124.05 118.04 117.23 114.73 111.81 107.56 71.62 70.97 56.29 23.30
OMe
O
Br
Me
O
OBn
10a
^{13}C NMR
(101 MHz, CDCl$_3$)
210 200 190 180 170 160 150 140 130 120 110 100 90 80 70 60 50 40 30 20 10 0
f1 (ppm)

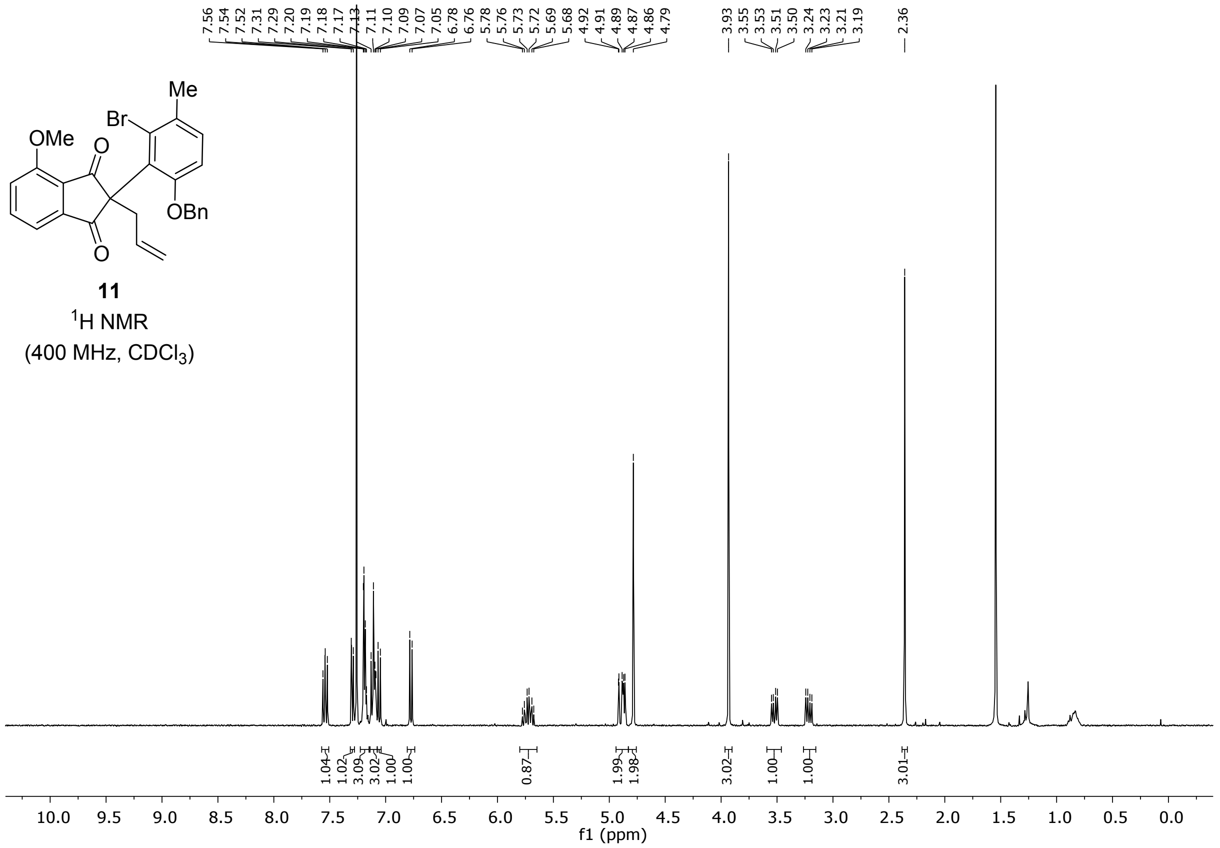
7.56 7.54 7.52 7.31 7.29 7.20 7.19 7.18 7.17 7.13 7.11 7.10 7.09 7.07 7.05 6.78 6.76 5.78 5.76 5.73 5.72 5.69 5.68 4.92 4.91 4.89 4.87 4.86 4.79
3.93 3.55 3.53 3.51 3.50 3.24 3.23 3.21 3.19
2.36
Me
Br
OMe
O
OBn
O
11
^{1}H NMR
(400 MHz, CDCl$_3$)
1.04 1.02 3.09 3.02 1.00 1.00 0.87 1.99 1.98 3.02 1.00 1.00 3.01
10.0 9.5 9.0 8.5 8.0 7.5 7.0 6.5 6.0 5.5 5.0 4.5 4.0 3.5 3.0 2.5 2.0 1.5 1.0 0.5 0.0
f1 (ppm)

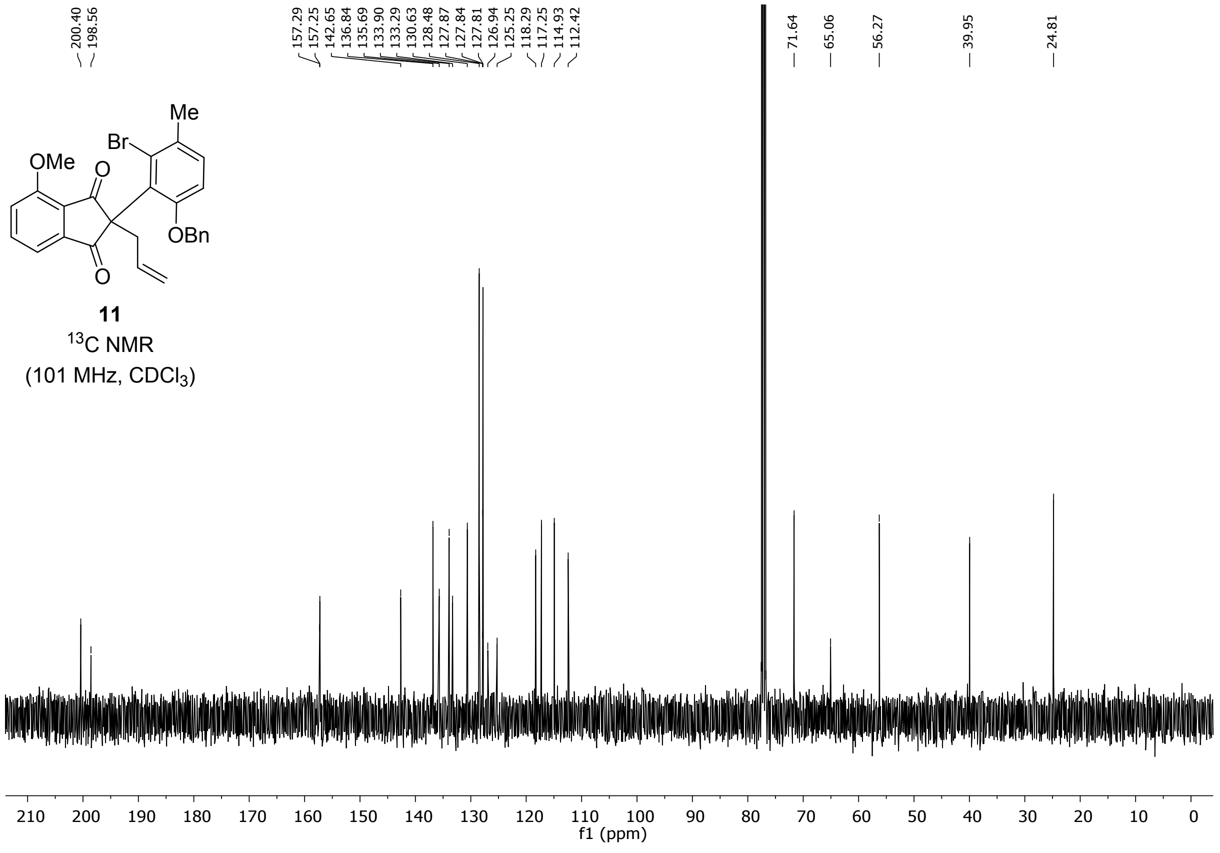
200.40 198.56
157.29 157.25 142.65 136.84 135.69 133.90 133.29 130.63 128.48 127.87 127.84 127.81 126.94 125.25 118.29 117.25 114.93 112.42
71.64 65.06 56.27 39.95 24.81
Me
Br
OMe
O
OBn
O
11
^{13}C NMR
(101 MHz, CDCl$_3$)
210 200 190 180 170 160 150 140 130 120 110 100 90 80 70 60 50 40 30 20 10 0
f1 (ppm)

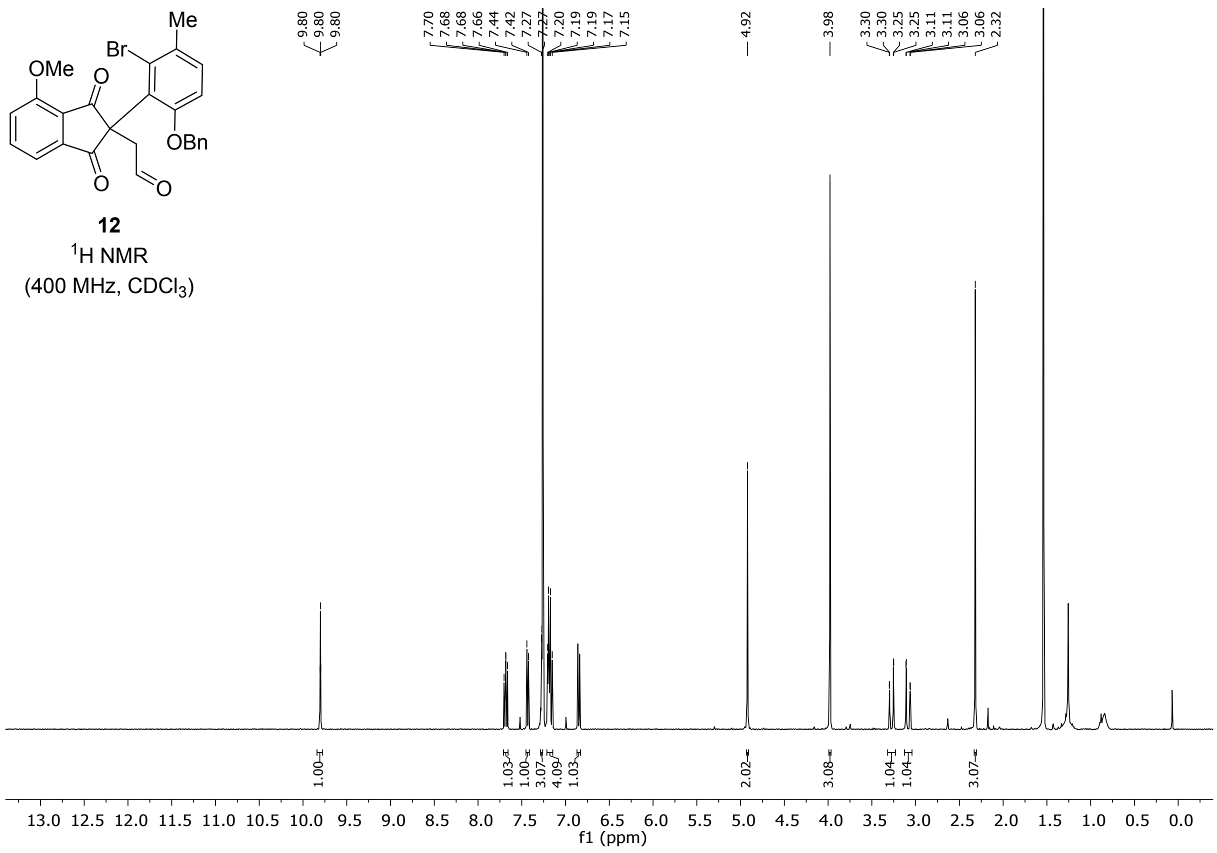
Me
Br
OMe
O
OBn
O
O
12
^{1}H NMR
(400 MHz, $CDCl_3$)
9.80 9.80 9.80
7.70 7.68 7.68 7.66 7.44 7.42 7.27 7.27 7.20 7.19 7.19 7.17 7.15
4.92
3.98
3.30 3.30 3.25 3.25 3.11 3.11 3.06 3.06 2.32
1.00
1.03 1.00 3.07 4.09 1.03
2.02
3.08
1.04 1.04
3.07
13.0 12.5 12.0 11.5 11.0 10.5 10.0 9.5 9.0 8.5 8.0 7.5 7.0 6.5 6.0 5.5 5.0 4.5 4.0 3.5 3.0 2.5 2.0 1.5 1.0 0.5 0.0
f1 (ppm)

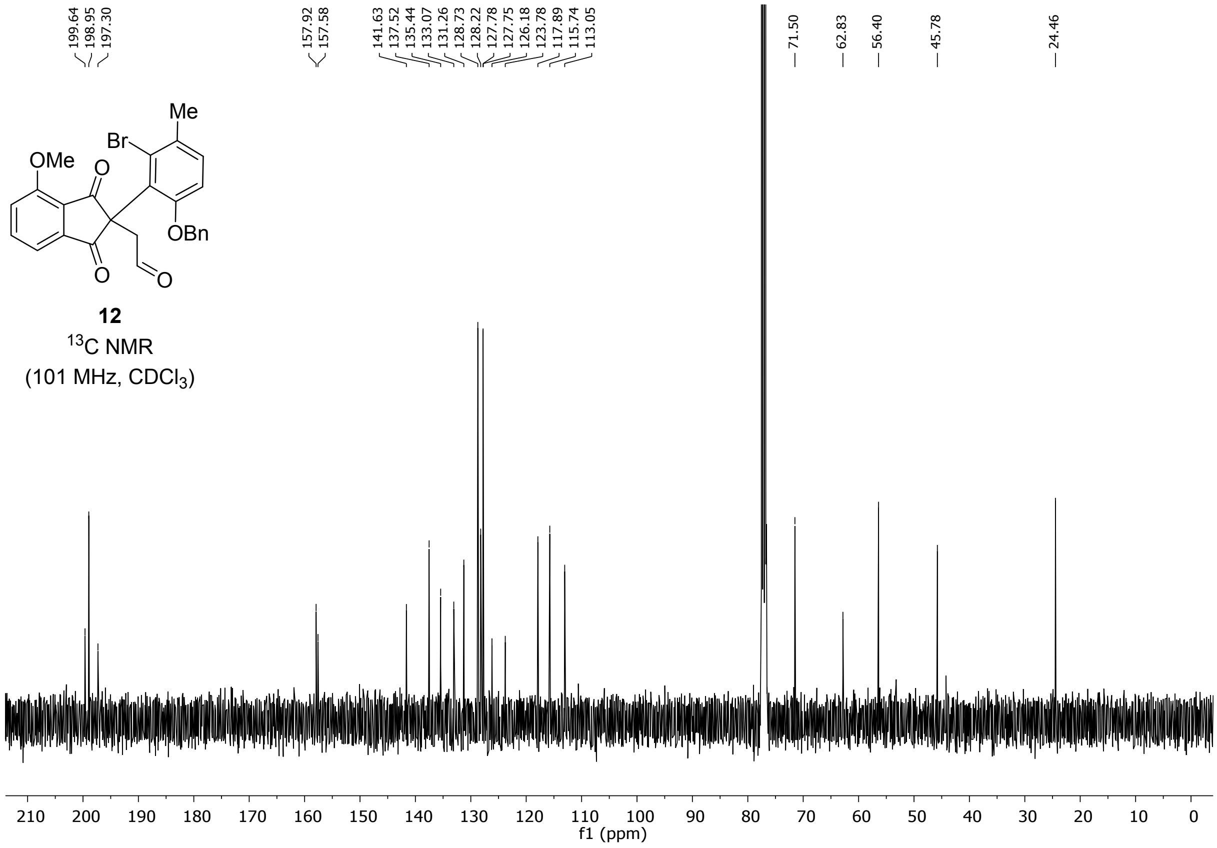
199.64 198.95 197.30
157.92 157.58
141.63 137.52 135.44 133.07 131.26 128.73 128.22 127.78 127.75 126.18 123.78 117.89 115.74 113.05
71.50
62.83
56.40
45.78
24.46
Me
Br
OMe
O
OBn
O
O
12
^{13}C NMR
(101 MHz, $CDCl_3$)
210 200 190 180 170 160 150 140 130 120 110 100 90 80 70 60 50 40 30 20 10 0
f1 (ppm)

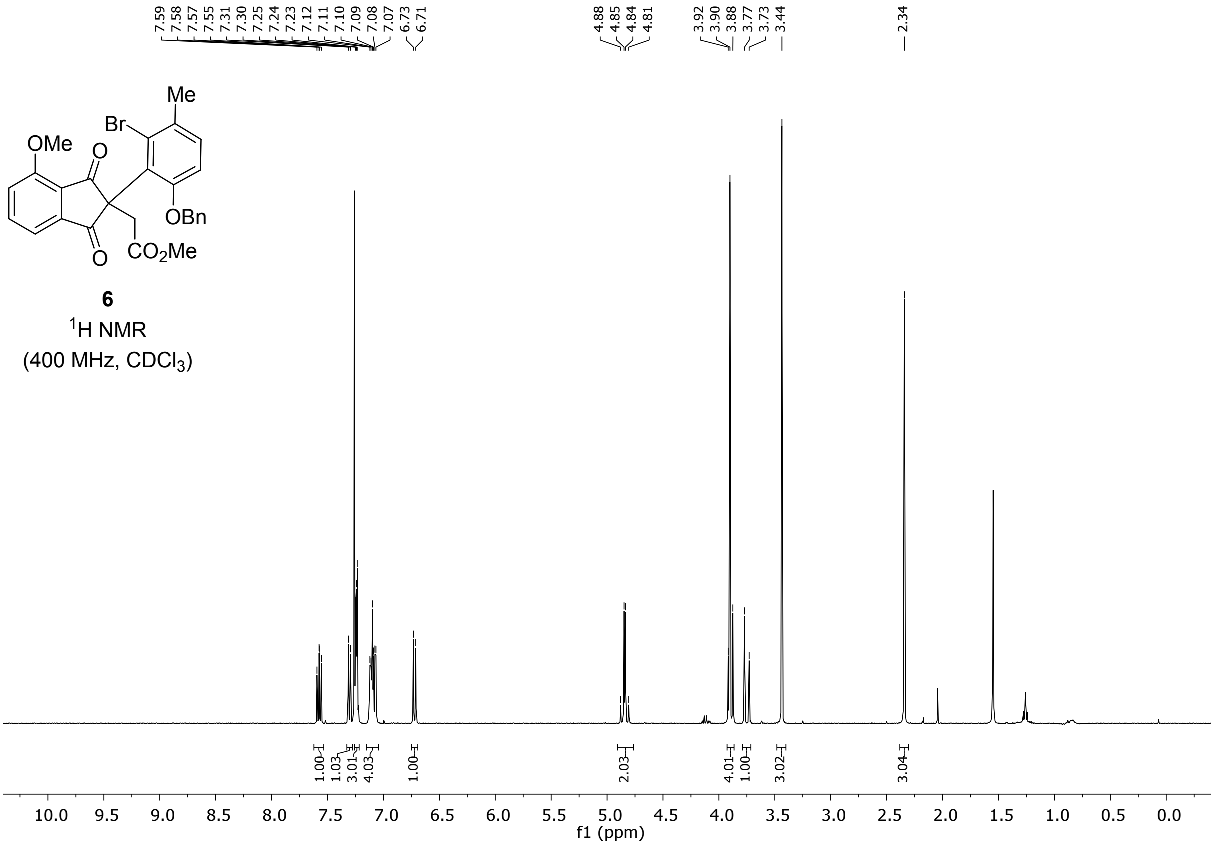
7.59 7.58 7.57 7.55 7.31 7.30 7.25 7.24 7.23 7.12 7.11 7.10 7.09 7.08 7.07 6.73 6.71
4.88 4.85 4.84 4.81
3.92 3.90 3.88 3.77 3.73 3.44
2.34
Me
Br
OMe
O
OBn
O
CO2Me
6
1H NMR
(400 MHz, CDCl3)
1.00 1.03 3.01 4.03 1.00 2.03 4.01 1.00 3.02 3.04
10.0 9.5 9.0 8.5 8.0 7.5 7.0 6.5 6.0 5.5 5.0 4.5 4.0 3.5 3.0 2.5 2.0 1.5 1.0 0.5 0.0
f1 (ppm)

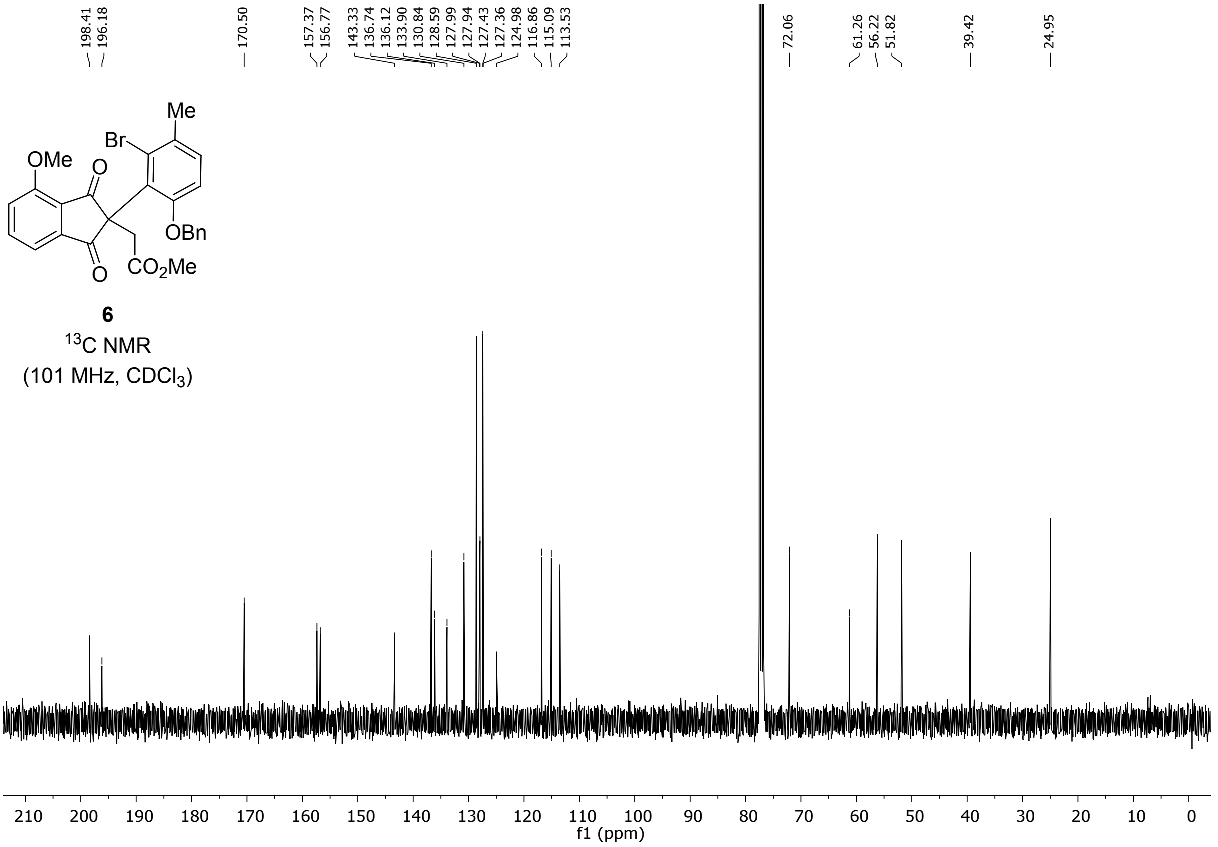
198.41 196.18
170.50
157.37 156.77
143.33 136.74 136.12 133.90 130.84 128.59 127.99 127.94 127.43 127.36 124.98 116.86 115.09 113.53
72.06
61.26 56.22 51.82
39.42
24.95
Me
Br
OMe
O
OBn
O
CO2Me
6
13C NMR
(101 MHz, CDCl3)
210 200 190 180 170 160 150 140 130 120 110 100 90 80 70 60 50 40 30 20 10 0
f1 (ppm)

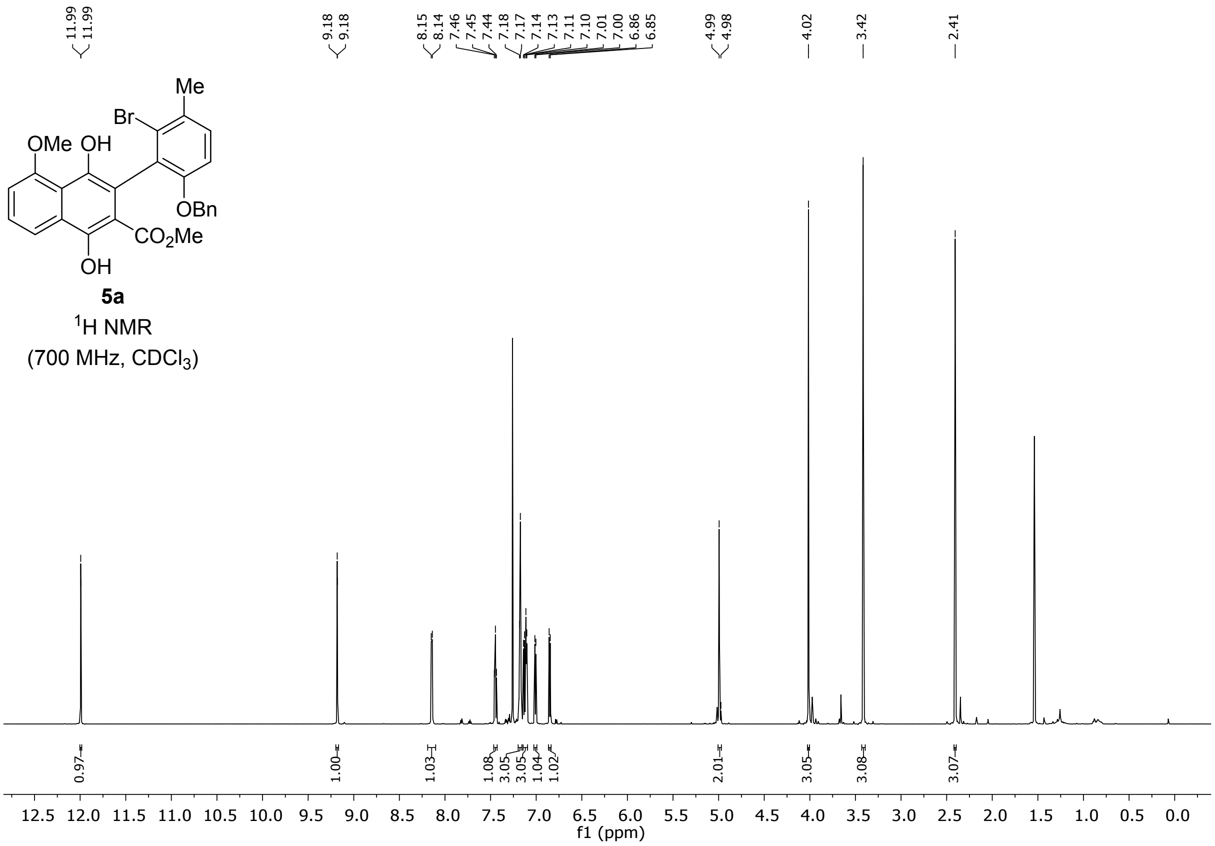
11.99
11.99
9.18
9.18
8.15
8.14
7.46
7.45
7.44
7.18
7.17
7.14
7.13
7.11
7.10
7.01
7.00
6.86
6.85
4.99
4.98
4.02
3.42
2.41
Me
Br
OMe OH
OBn
CO2Me
OH
5a
1H NMR
(700 MHz, CDCl3)
0.97
1.00
1.03
1.08
3.05
3.05
1.04
1.02
2.01
3.05
3.08
3.07
f1 (ppm)

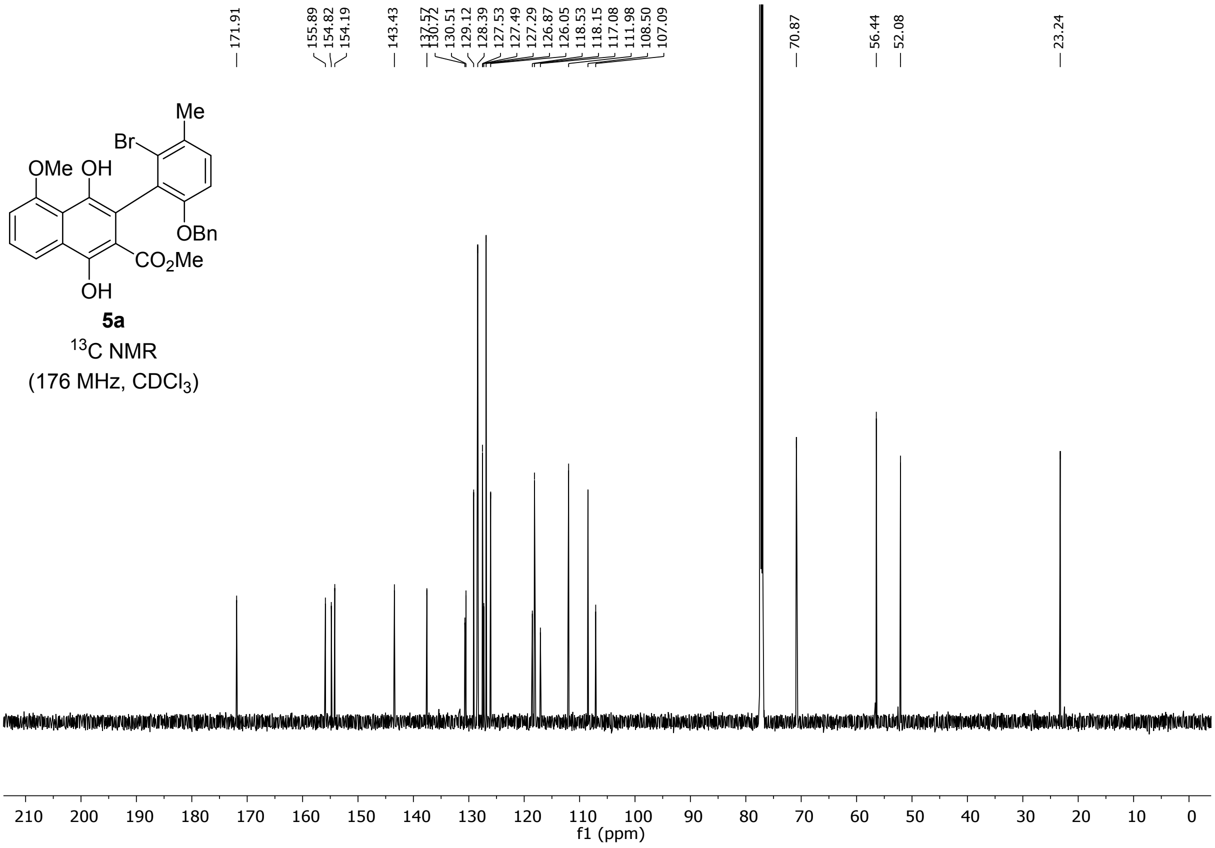
171.91
155.89
154.82
154.19
143.43
137.57
130.72
130.51
129.12
128.39
127.53
127.49
127.29
126.87
126.05
118.53
118.15
117.08
111.98
108.50
107.09
70.87
56.44
52.08
23.24
Me
Br
OMe OH
OBn
CO2Me
OH
5a
13C NMR
(176 MHz, CDCl3)
f1 (ppm)

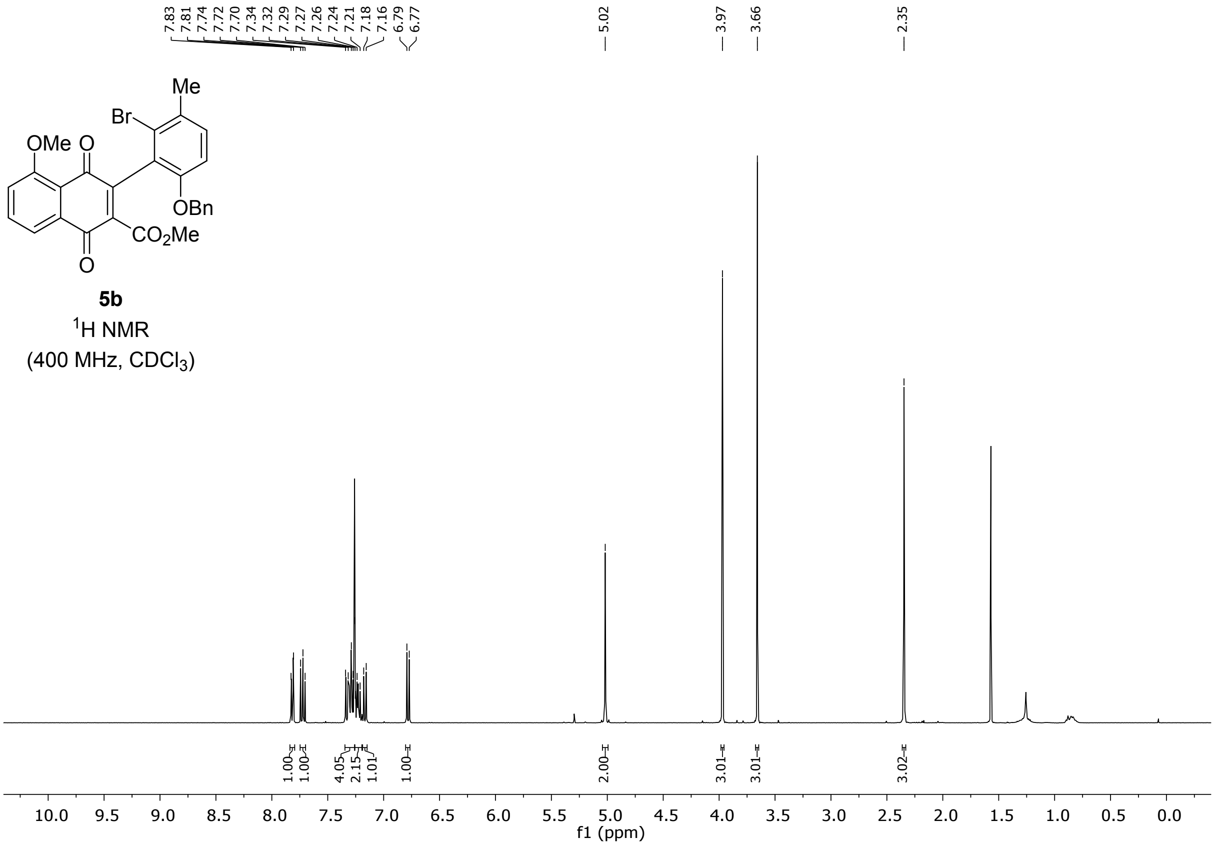
7.83 7.81 7.74 7.72 7.70 7.34 7.32 7.29 7.27 7.26 7.24 7.21 7.18 7.16 6.79 6.77
5.02
3.97
3.66
2.35
Me
Br
OMe O
OBn
CO2Me
O
5b
1H NMR
(400 MHz, CDCl3)
1.00 1.00 4.05 2.15 1.01 1.00 2.00 3.01 3.01 3.02
f1 (ppm)

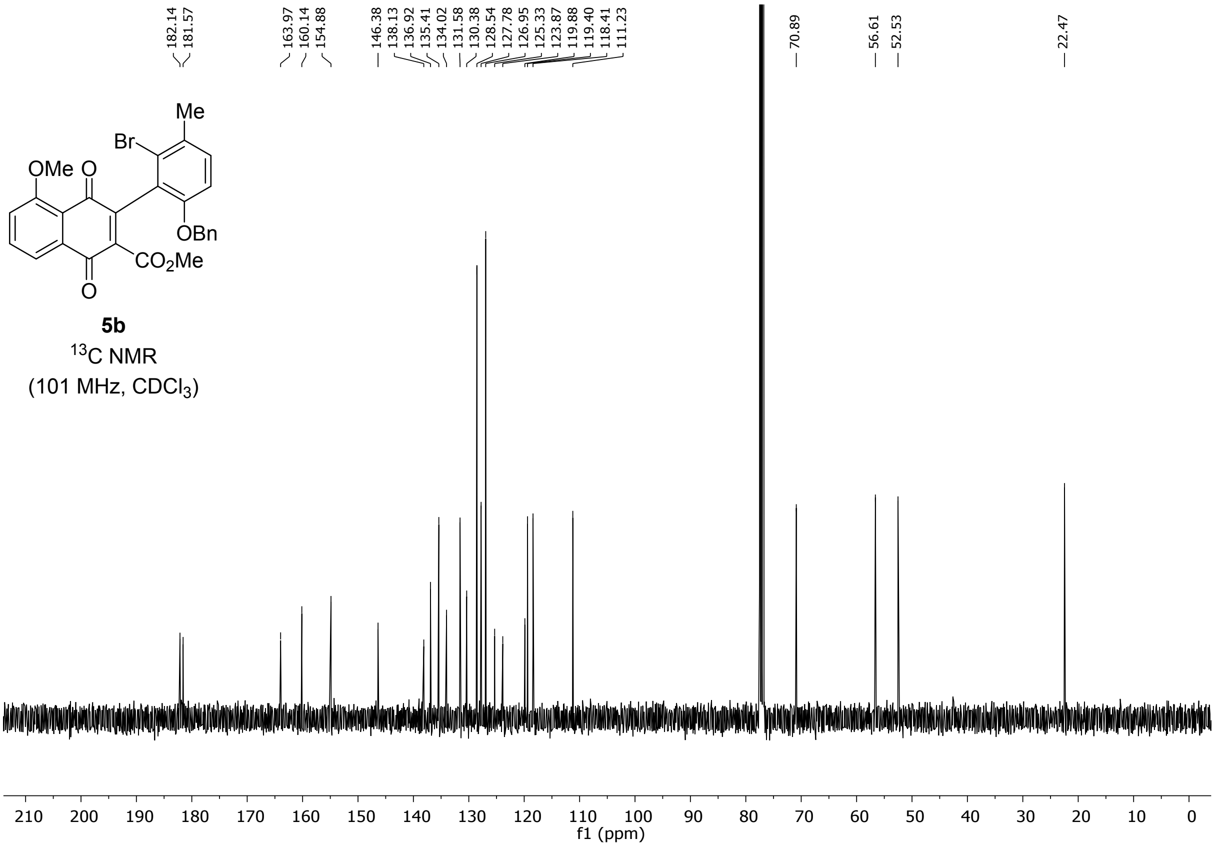
182.14 181.57
163.97 160.14 154.88
146.38 138.13 136.92 135.41 134.02 131.58 130.38 128.54 127.78 126.95 125.33 123.87 119.88 119.40 118.41 111.23
70.89
56.61 52.53
22.47
Me
Br
OMe O
OBn
CO2Me
O
5b
13C NMR
(101 MHz, CDCl3)
f1 (ppm)

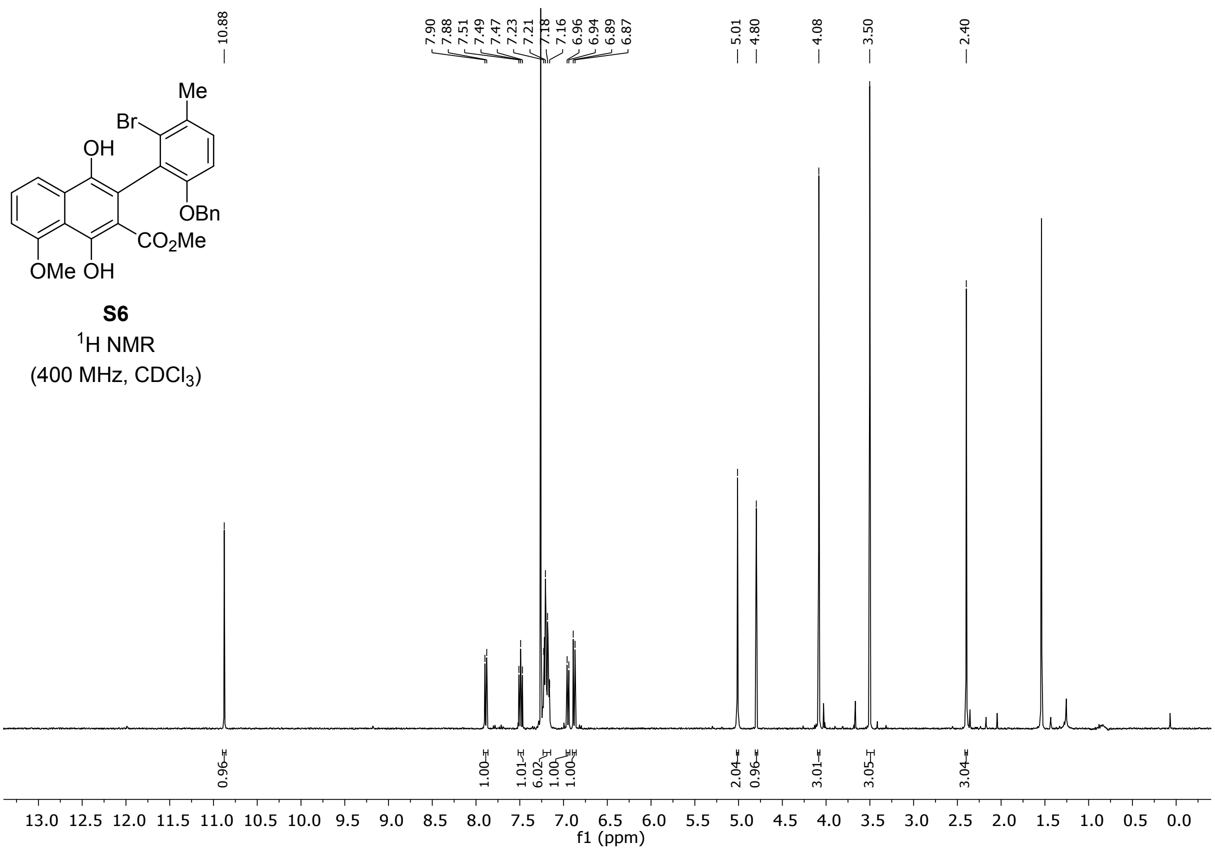
10.88
7.90
7.88
7.51
7.49
7.47
7.23
7.21
7.18
7.16
6.96
6.94
6.89
6.87
5.01
4.80
4.08
3.50
2.40
Me
Br
OH
OBn
CO2Me
OMe OH
S6
1H NMR
(400 MHz, CDCl3)
0.96
1.00
1.01
6.02
1.00
1.00
2.04
0.96
3.01
3.05
3.04
13.0 12.5 12.0 11.5 11.0 10.5 10.0 9.5 9.0 8.5 8.0 7.5 7.0 6.5 6.0 5.5 5.0 4.5 4.0 3.5 3.0 2.5 2.0 1.5 1.0 0.5 0.0
f1 (ppm)

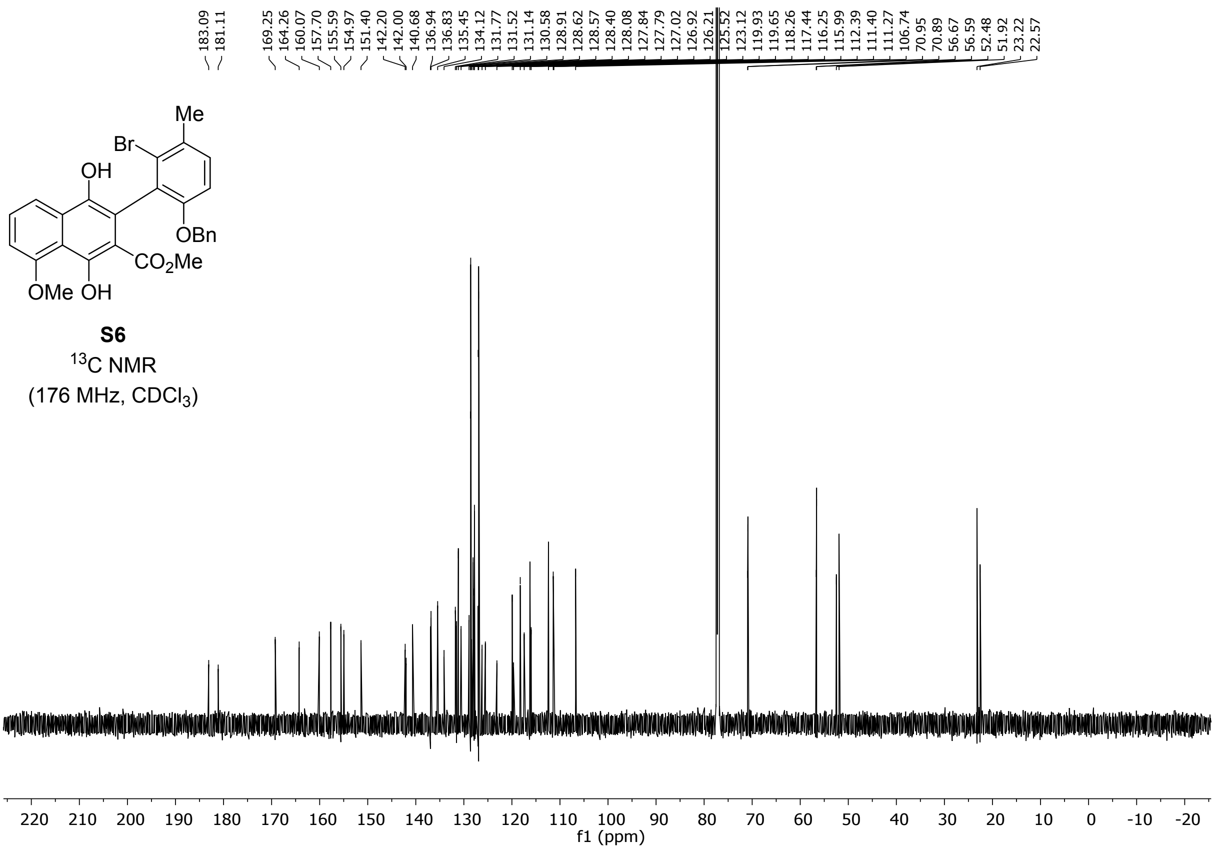
183.09
181.11
169.25
164.26
160.07
157.70
155.59
154.97
151.40
142.20
142.00
140.68
136.94
136.83
135.45
134.12
131.77
131.52
131.14
130.58
128.91
128.62
128.57
128.40
128.08
127.84
127.79
127.02
126.92
126.21
125.52
123.12
119.93
119.65
118.26
117.44
116.25
115.99
112.39
111.40
111.27
106.74
70.95
70.89
56.67
56.59
52.48
51.92
23.22
22.57
Me
Br
OH
OBn
CO2Me
OMe OH
S6
13C NMR
(176 MHz, CDCl3)
220 210 200 190 180 170 160 150 140 130 120 110 100 90 80 70 60 50 40 30 20 10 0 -10 -20
f1 (ppm)

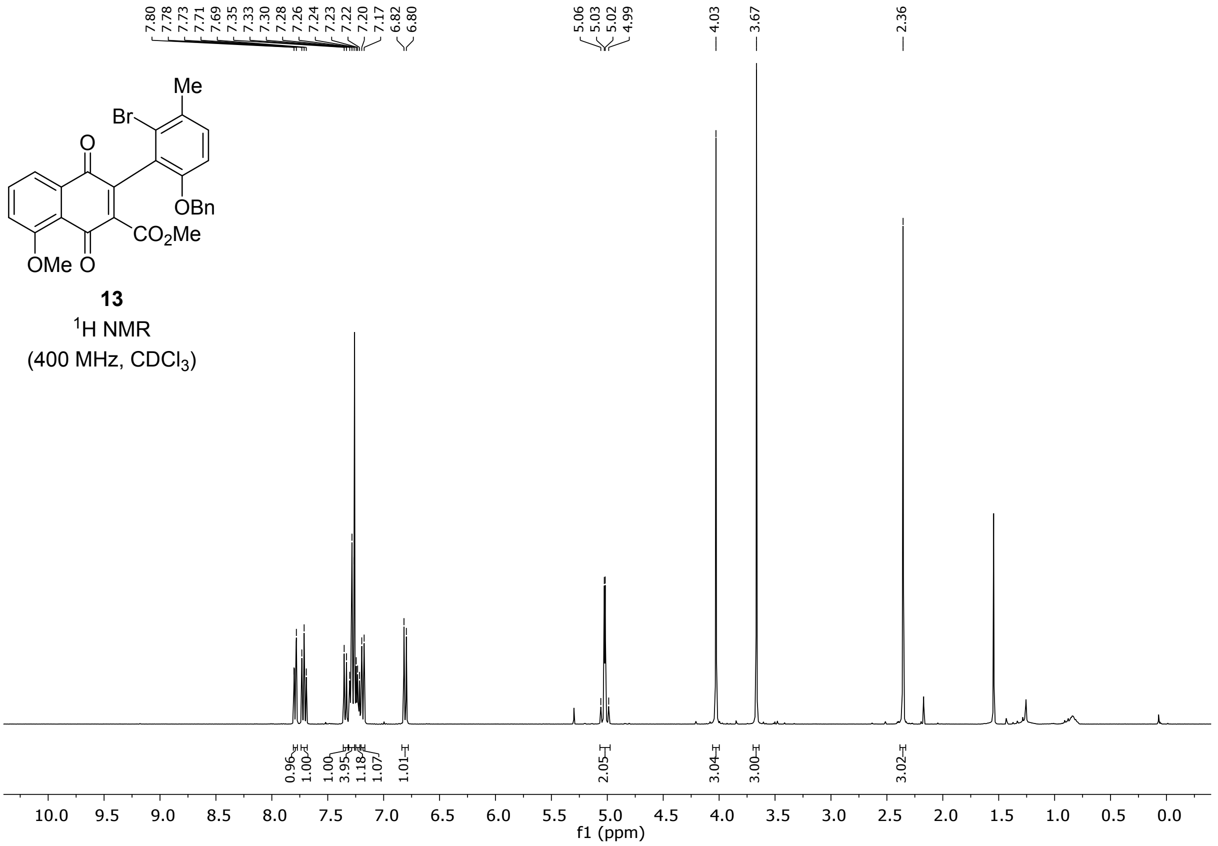
7.80 7.78 7.73 7.71 7.69 7.35 7.33 7.30 7.28 7.26 7.24 7.23 7.22 7.20 7.17 6.82 6.80
5.06 5.03 5.02 4.99
4.03
3.67
2.36
Me
Br
O
OBn
CO2Me
OMe O
13
1H NMR
(400 MHz, CDCl3)
0.96 1.00 1.00 3.95 1.18 1.07 1.01
2.05
3.04
3.00
3.02
10.0 9.5 9.0 8.5 8.0 7.5 7.0 6.5 6.0 5.5 5.0 4.5 4.0 3.5 3.0 2.5 2.0 1.5 1.0 0.5 0.0
f1 (ppm)

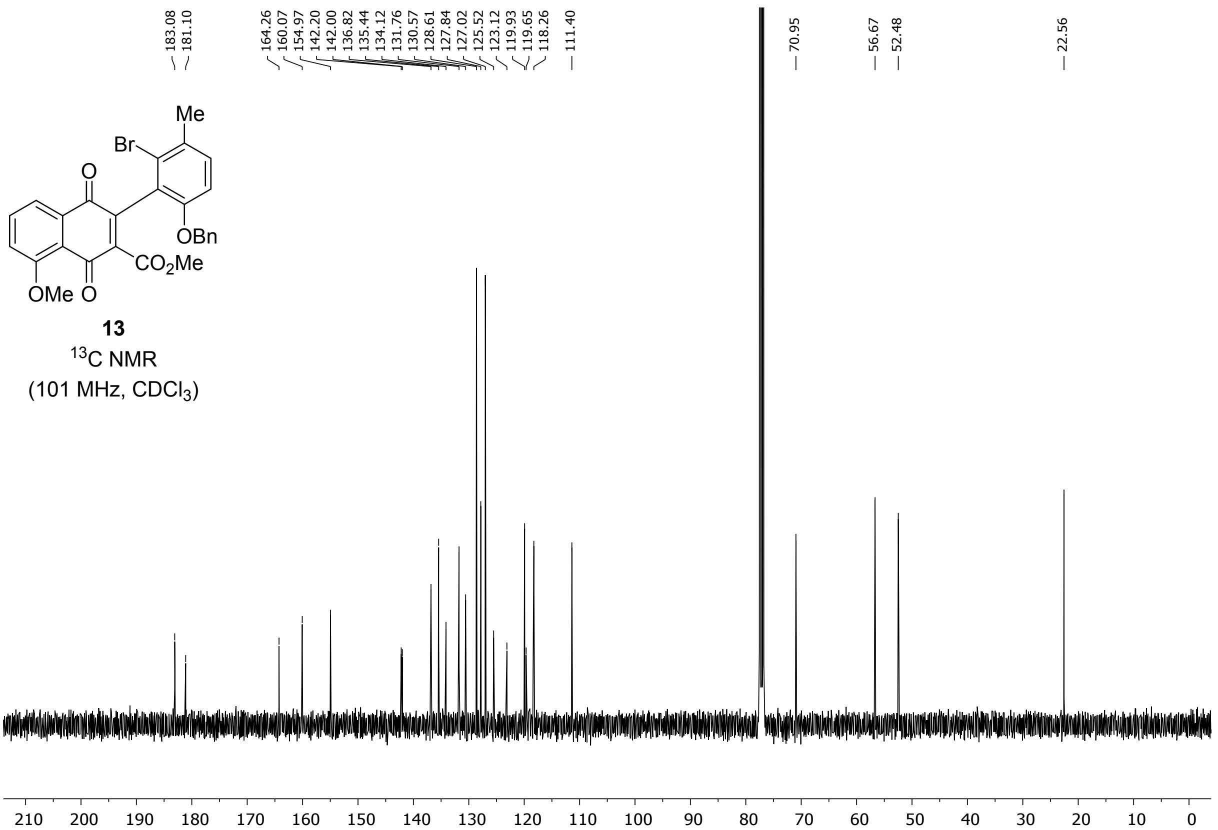
183.08 181.10
164.26 160.07 154.97 142.20 142.00 136.82 135.44 134.12 131.76 130.57 128.61 127.84 127.02 125.52 123.12 119.93 119.65 118.26
111.40
70.95
56.67 52.48
22.56
Me
Br
O
OBn
CO2Me
OMe O
13
13C NMR
(101 MHz, CDCl3)
210 200 190 180 170 160 150 140 130 120 110 100 90 80 70 60 50 40 30 20 10 0
f1 (ppm)

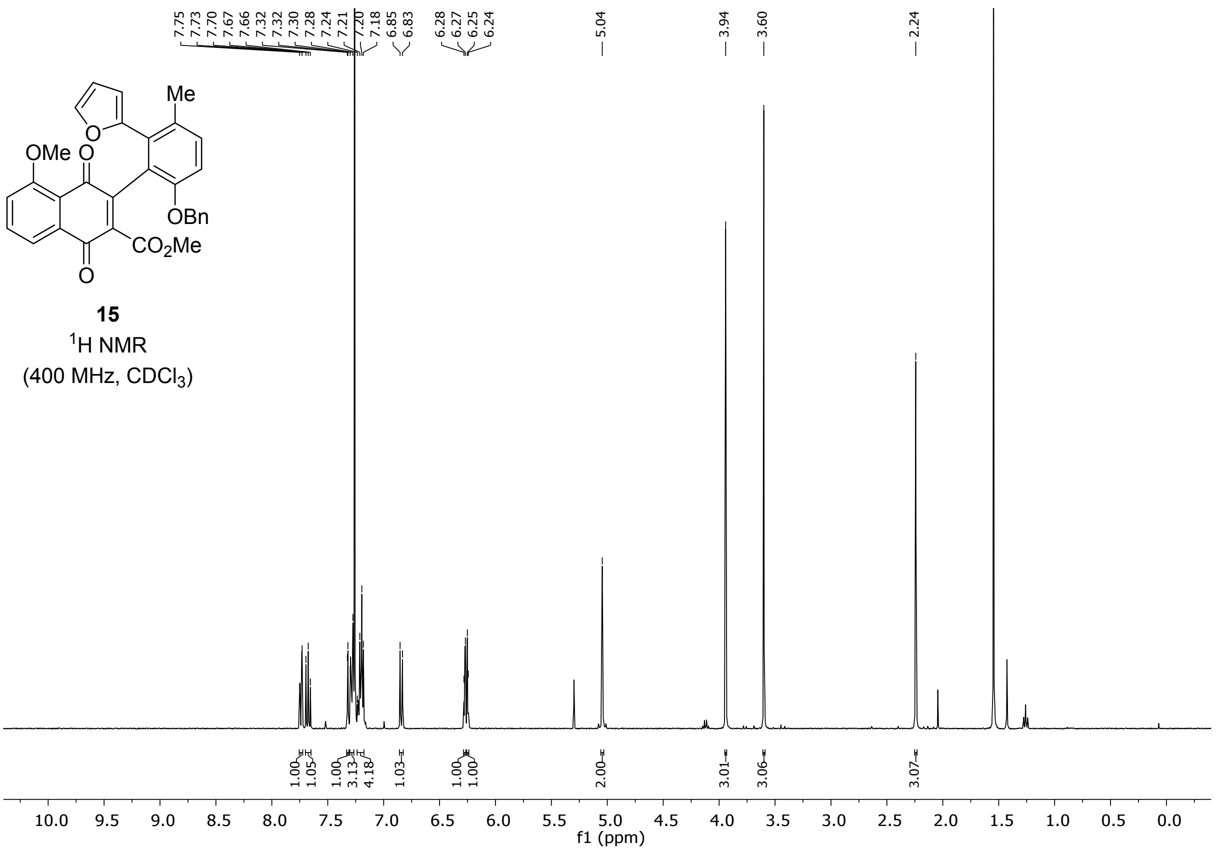
7.75 7.73 7.70 7.67 7.66 7.32 7.32 7.30 7.28 7.24 7.21 7.20 7.18 6.85 6.83 6.28 6.27 6.25 6.24
5.04
3.94
3.60
2.24
Me
OMe O
O
OBn
CO_2Me
O
15
^1H NMR
(400 MHz, $CDCl_3$)
1.00 1.05 1.00 3.13 4.18 1.03 1.00 1.00 2.00 3.01 3.06 3.07
10.0 9.5 9.0 8.5 8.0 7.5 7.0 6.5 6.0 5.5 5.0 4.5 4.0 3.5 3.0 2.5 2.0 1.5 1.0 0.5 0.0
f1 (ppm)

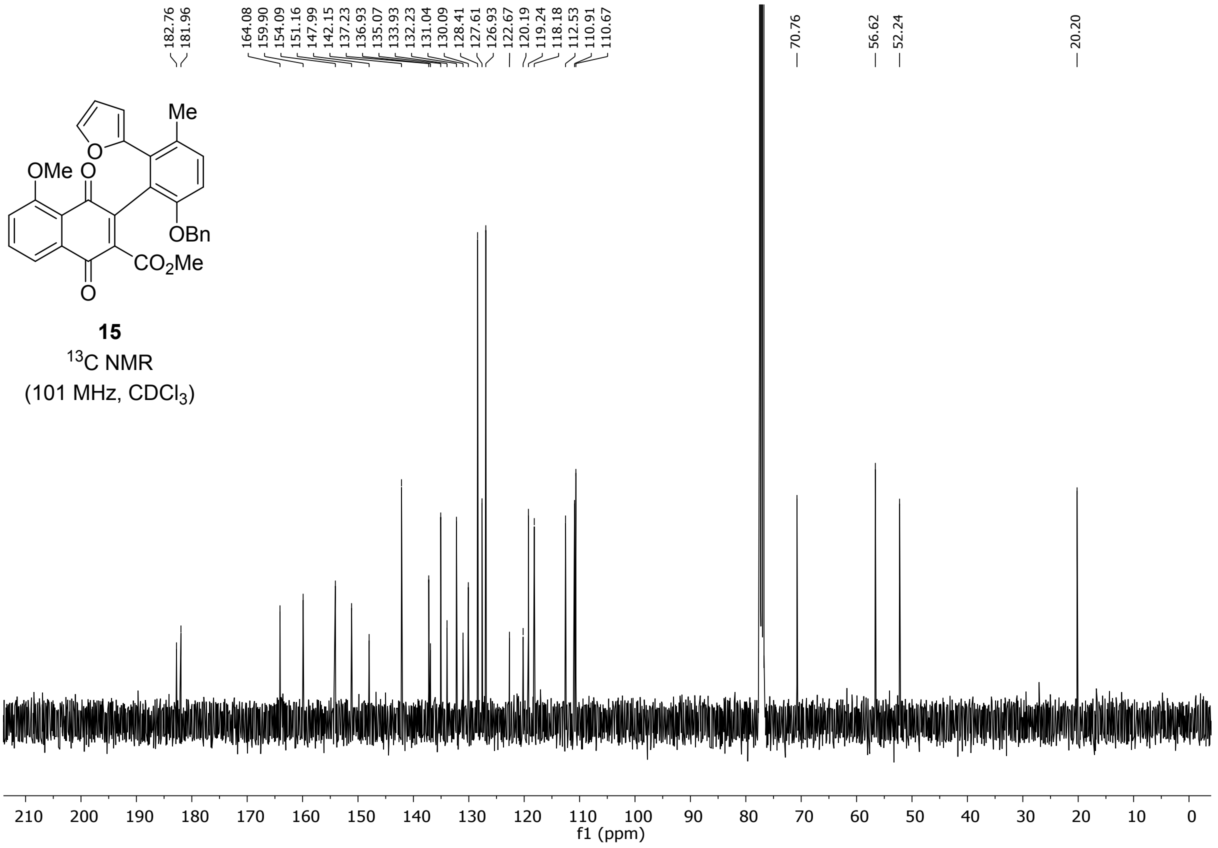
182.76 181.96
164.08 159.90 154.09 151.16 147.99 142.15 137.23 136.93 135.07 133.93 132.23 131.04 130.09 128.41 127.61 126.93 122.67 120.19 119.24 118.18 112.53 110.91 110.67
70.76
56.62 52.24
20.20
Me
OMe O
O
OBn
CO_2Me
O
15
^{13}C NMR
(101 MHz, $CDCl_3$)
210 200 190 180 170 160 150 140 130 120 110 100 90 80 70 60 50 40 30 20 10 0
f1 (ppm)

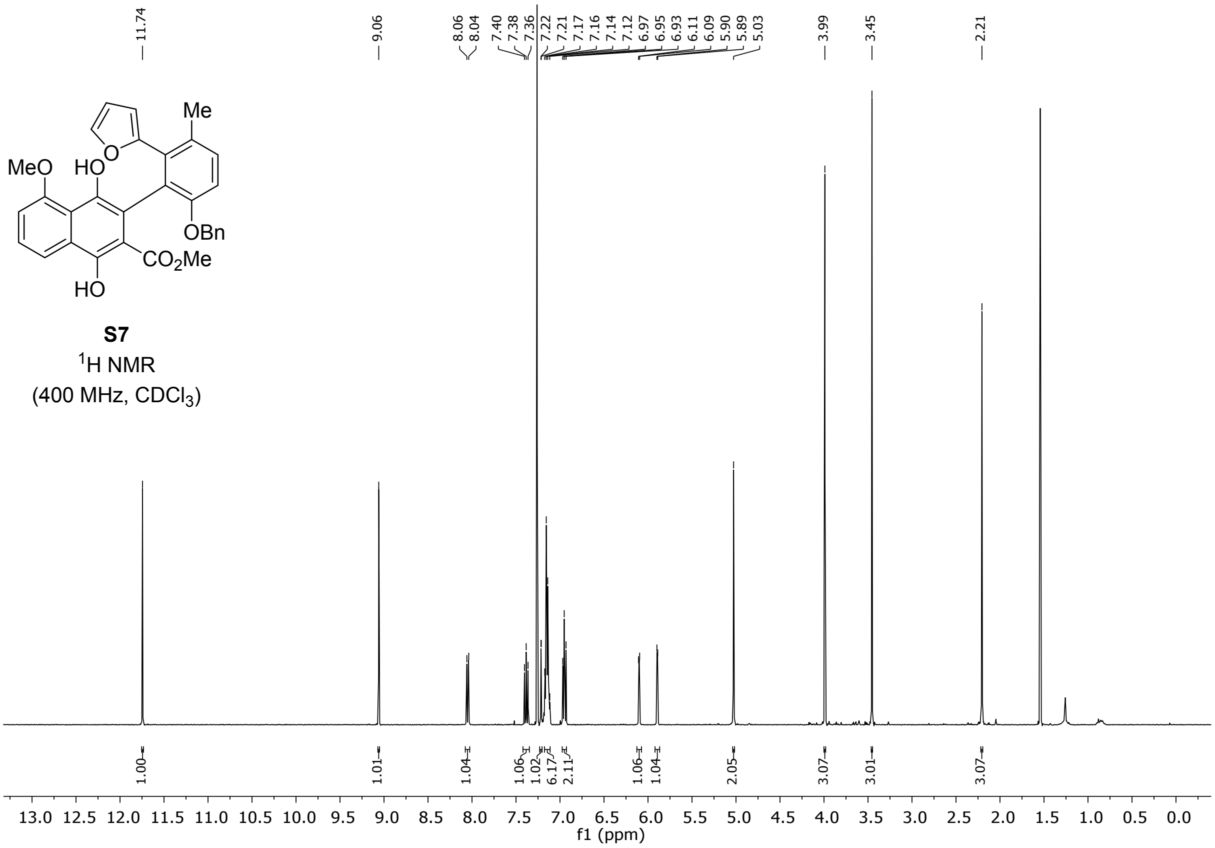
MeO
HO
Me
OBn
CO2Me
HO
S7
1H NMR
(400 MHz, CDCl3)
11.74
9.06
8.06
8.04
7.40
7.38
7.36
7.22
7.21
7.17
7.16
7.14
7.12
6.97
6.95
6.93
6.11
6.09
5.90
5.89
5.03
3.99
3.45
2.21
1.00
1.01
1.04
1.06
1.02
6.17
2.11
1.06
1.04
2.05
3.07
3.01
3.07
f1 (ppm)

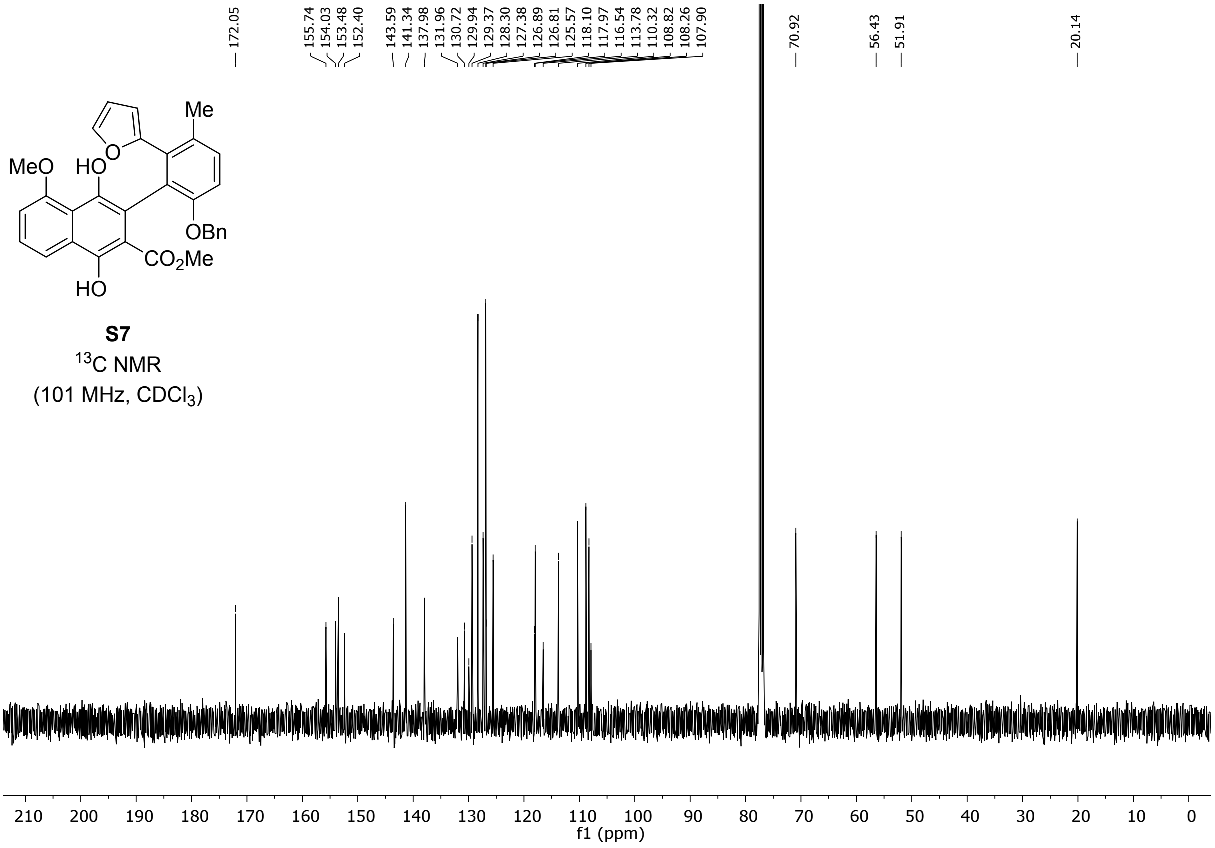
MeO
HO
Me
OBn
CO2Me
HO
S7
13C NMR
(101 MHz, CDCl3)
172.05
155.74
154.03
153.48
152.40
143.59
141.34
137.98
131.96
130.72
129.94
129.37
128.30
127.38
126.89
126.81
125.57
118.10
117.97
116.54
113.78
110.32
108.82
108.26
107.90
70.92
56.43
51.91
20.14
f1 (ppm)

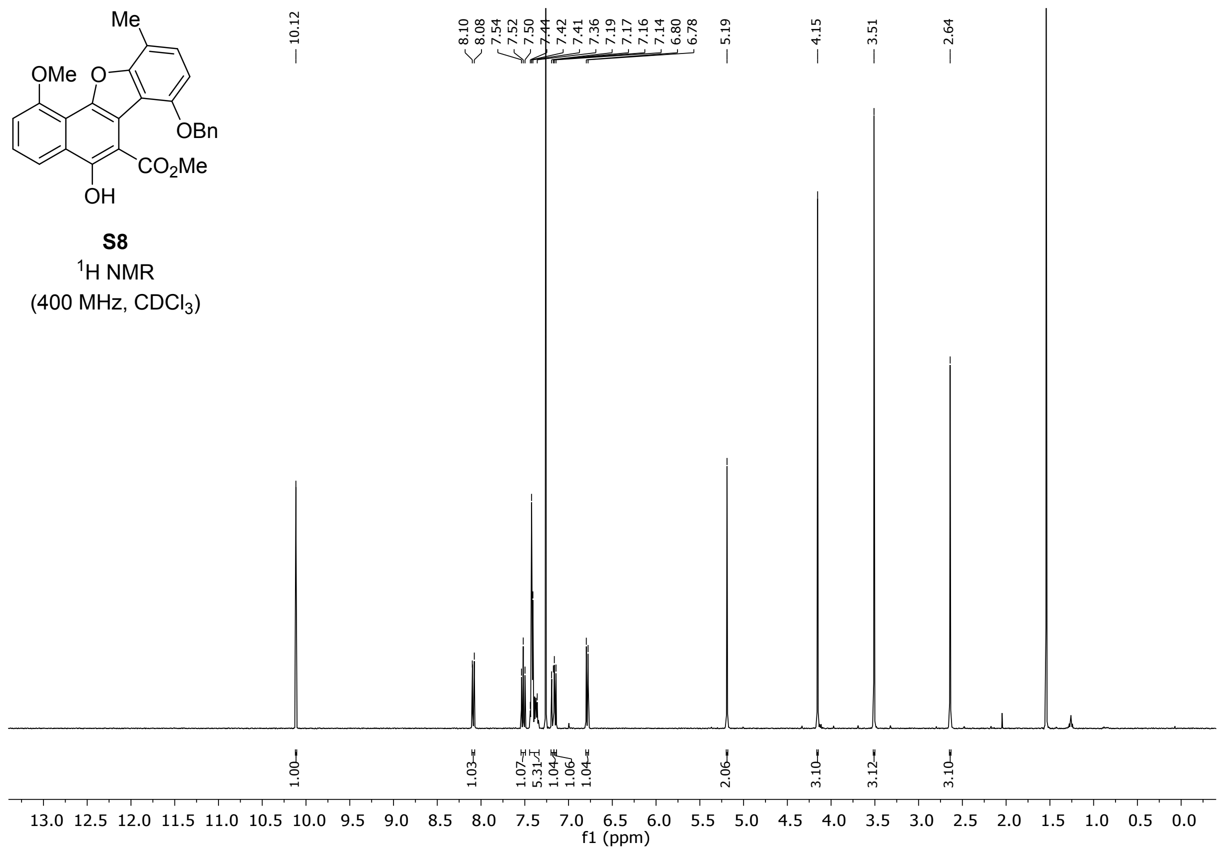

S8
^{1}H NMR
(400 MHz, $CDCl_3$)

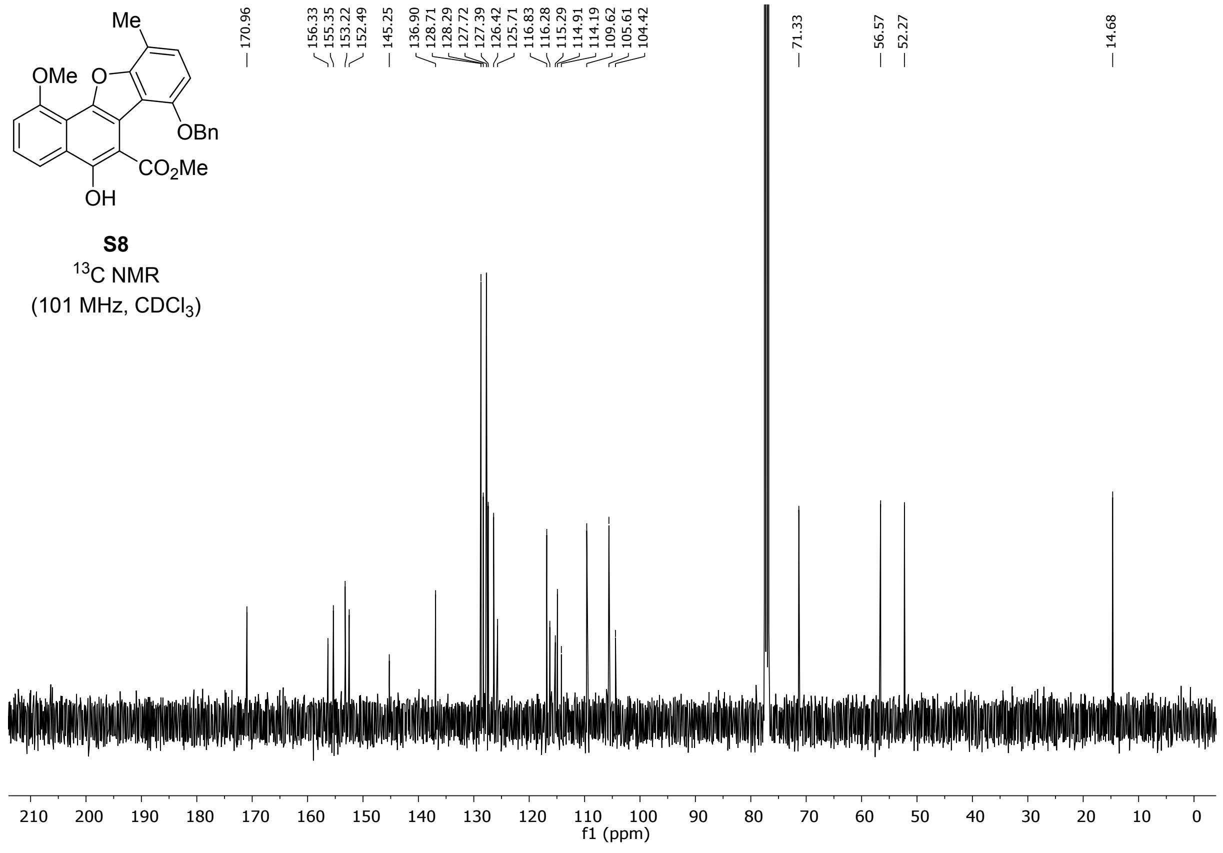

S8
^{13}C NMR
(101 MHz, $CDCl_3$)

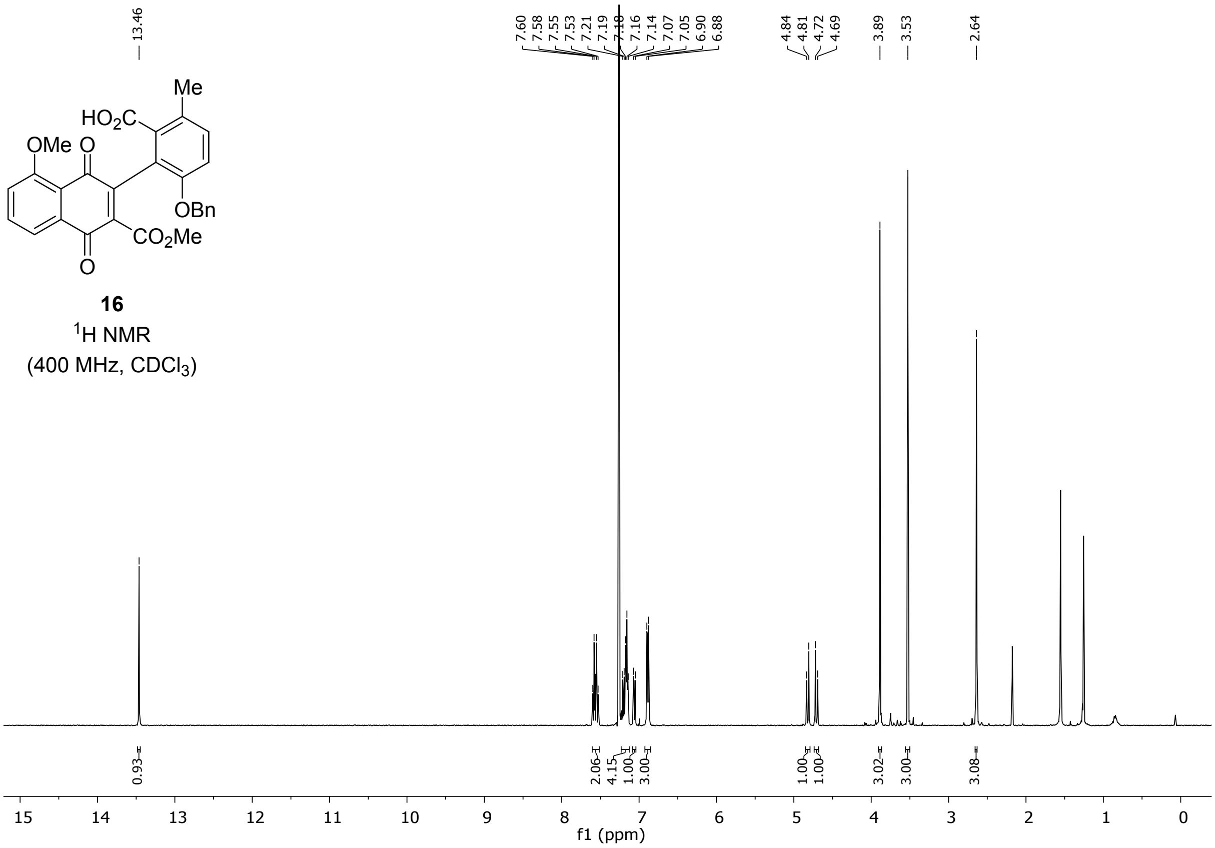

16
^{1}H NMR
(400 MHz, $CDCl_3$)

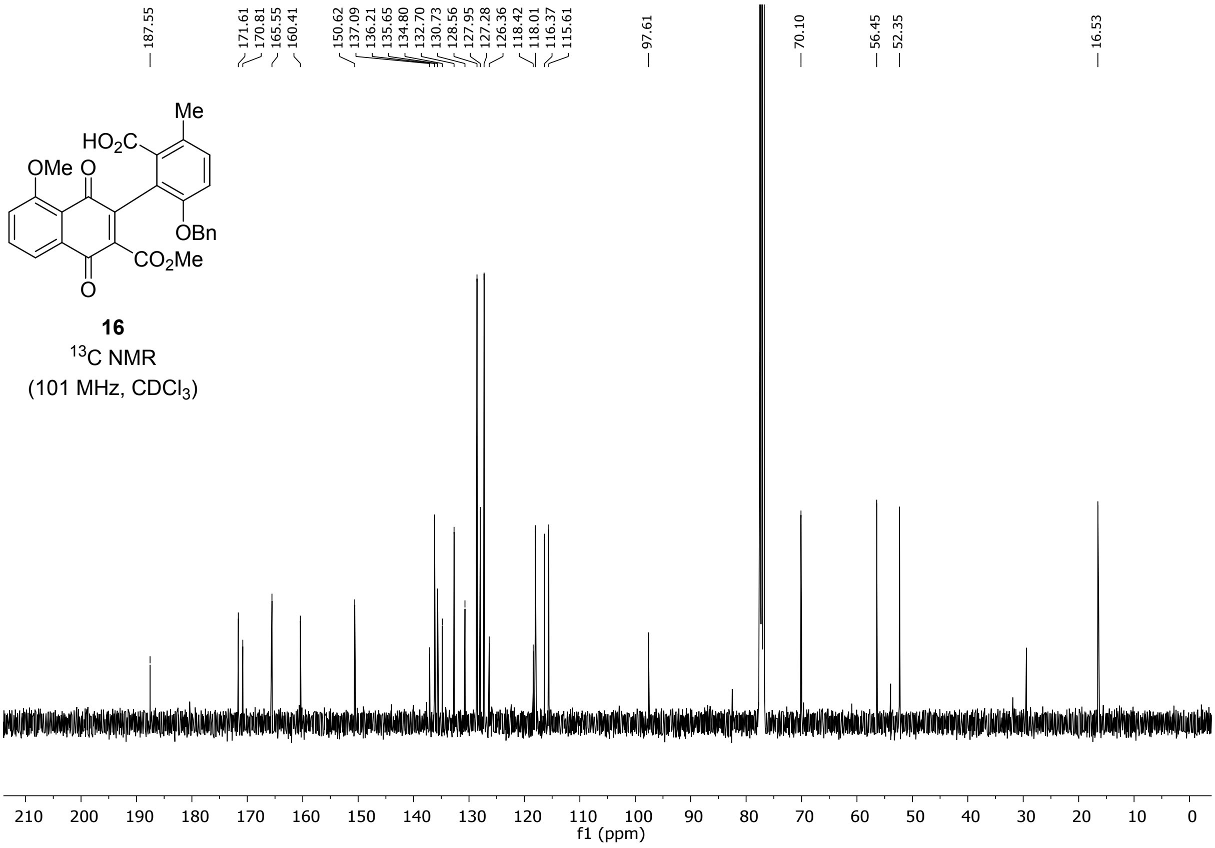

16
^{13}C NMR
(101 MHz, $CDCl_3$)

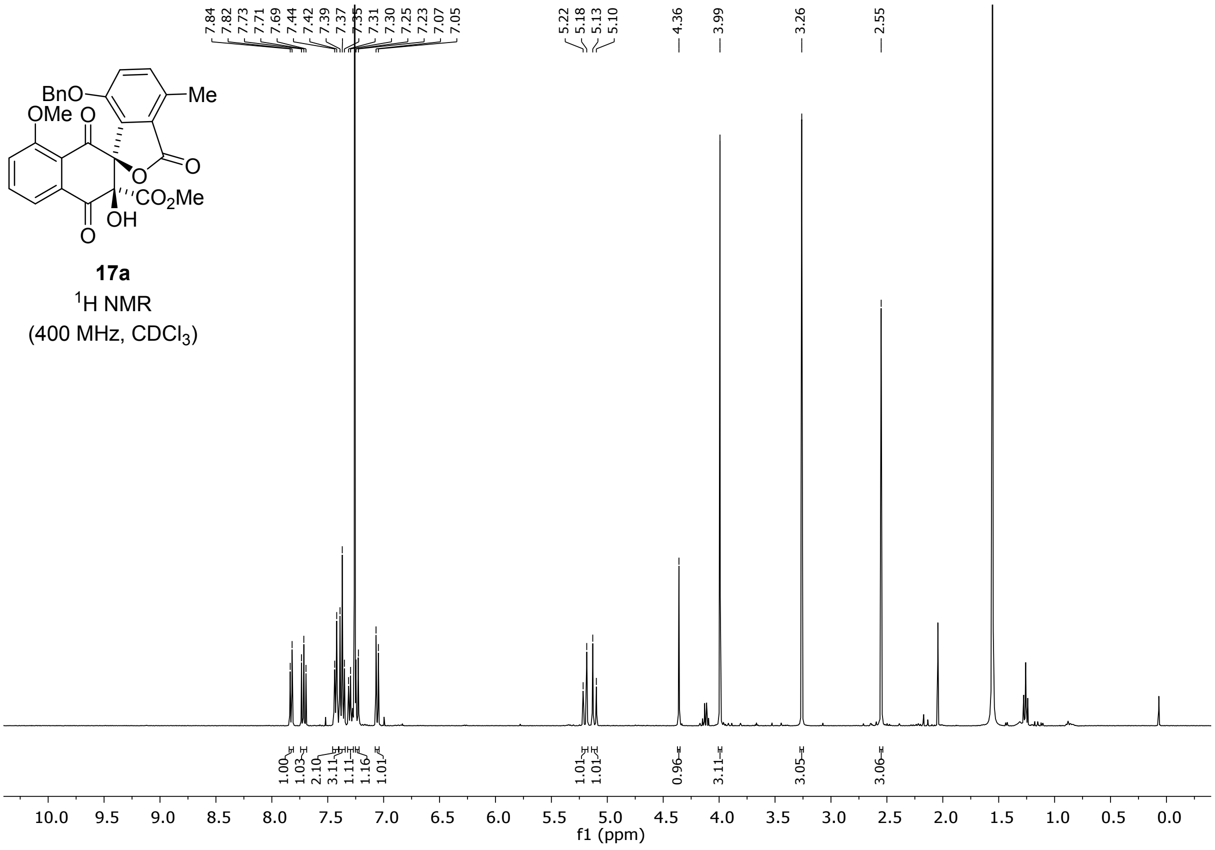

17a

^{1}H NMR

(400 MHz, $CDCl_3$)

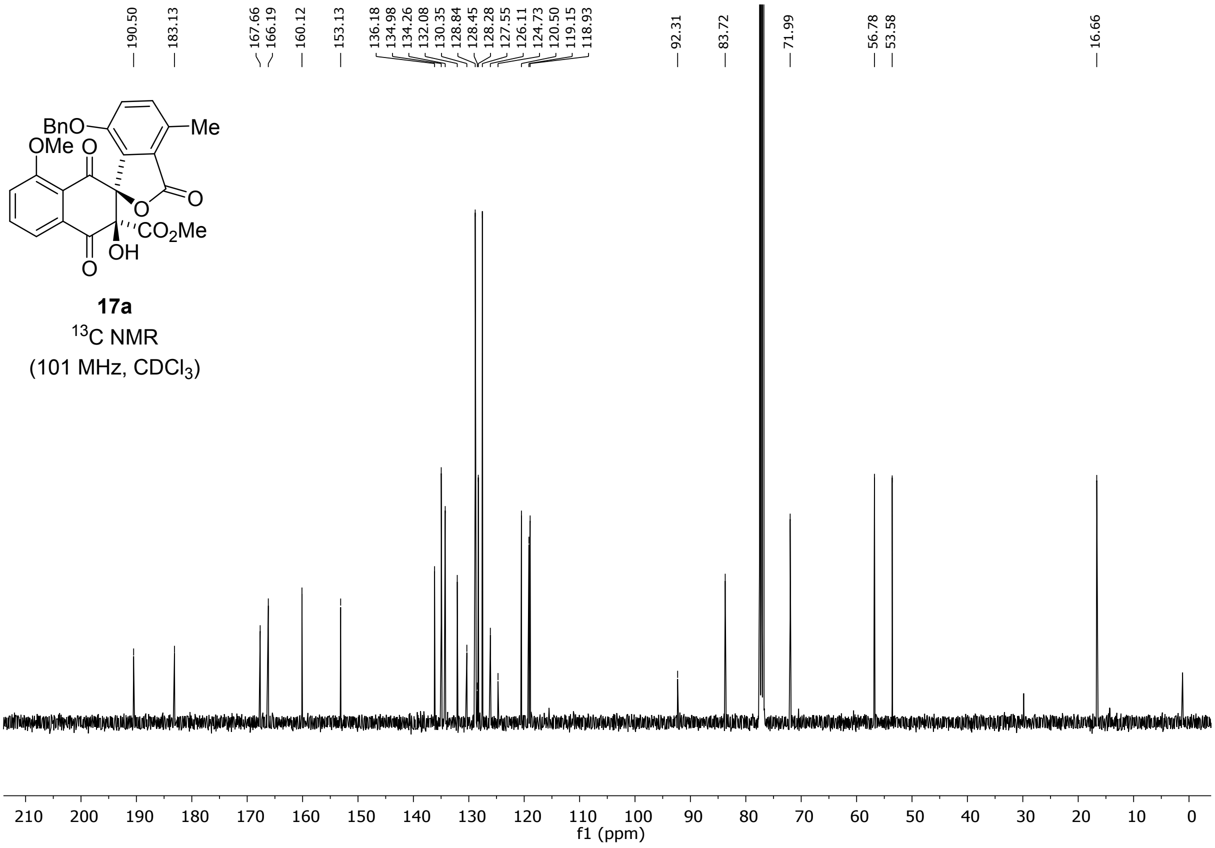

17a

^{13}C NMR

(101 MHz, $CDCl_3$)

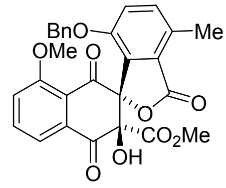

17b
^{1}H NMR
(700 MHz, $CDCl_3$)

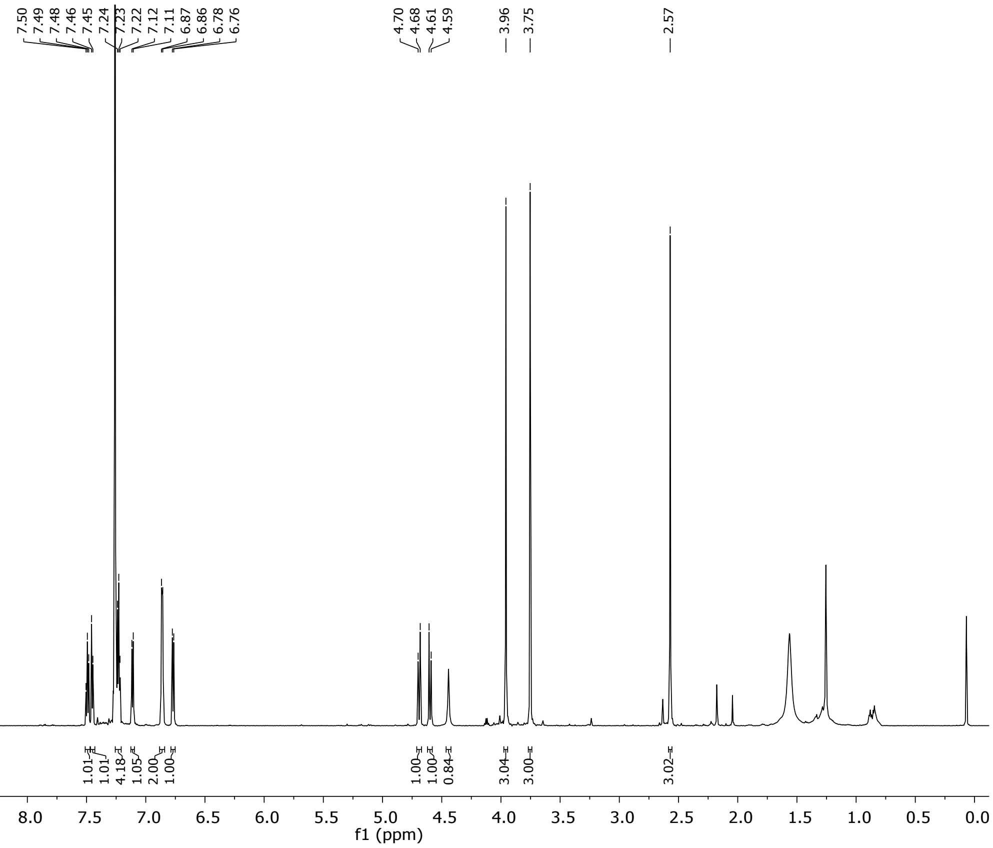

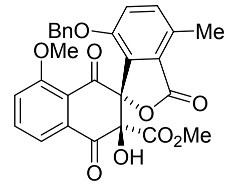

17b
^{13}C NMR
(176 MHz, $CDCl_3$)

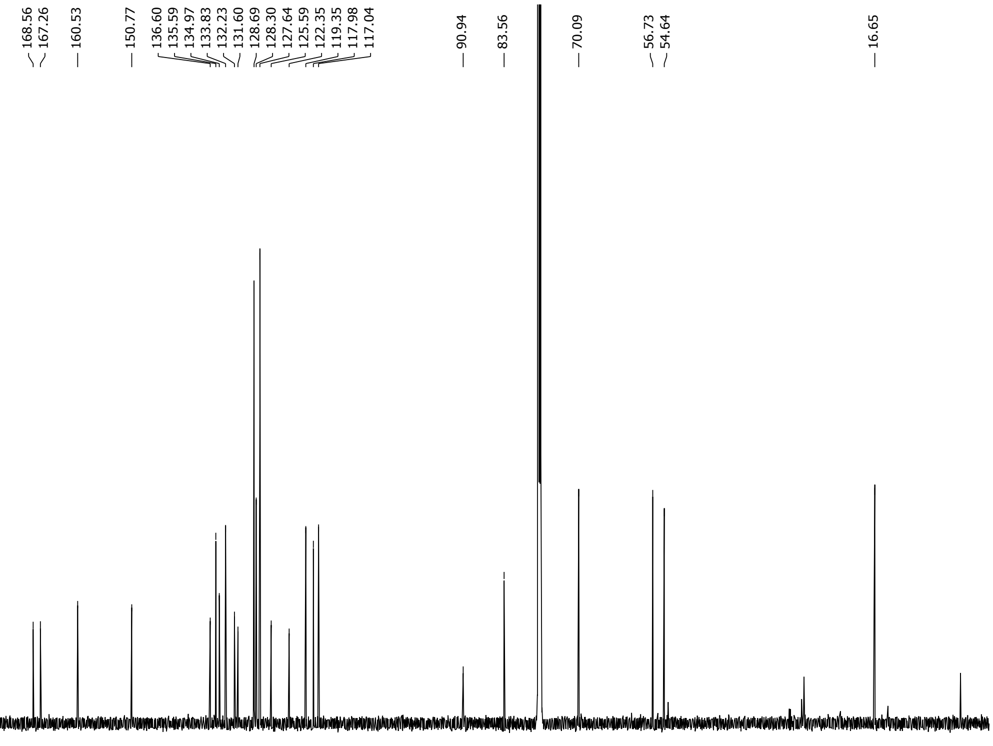

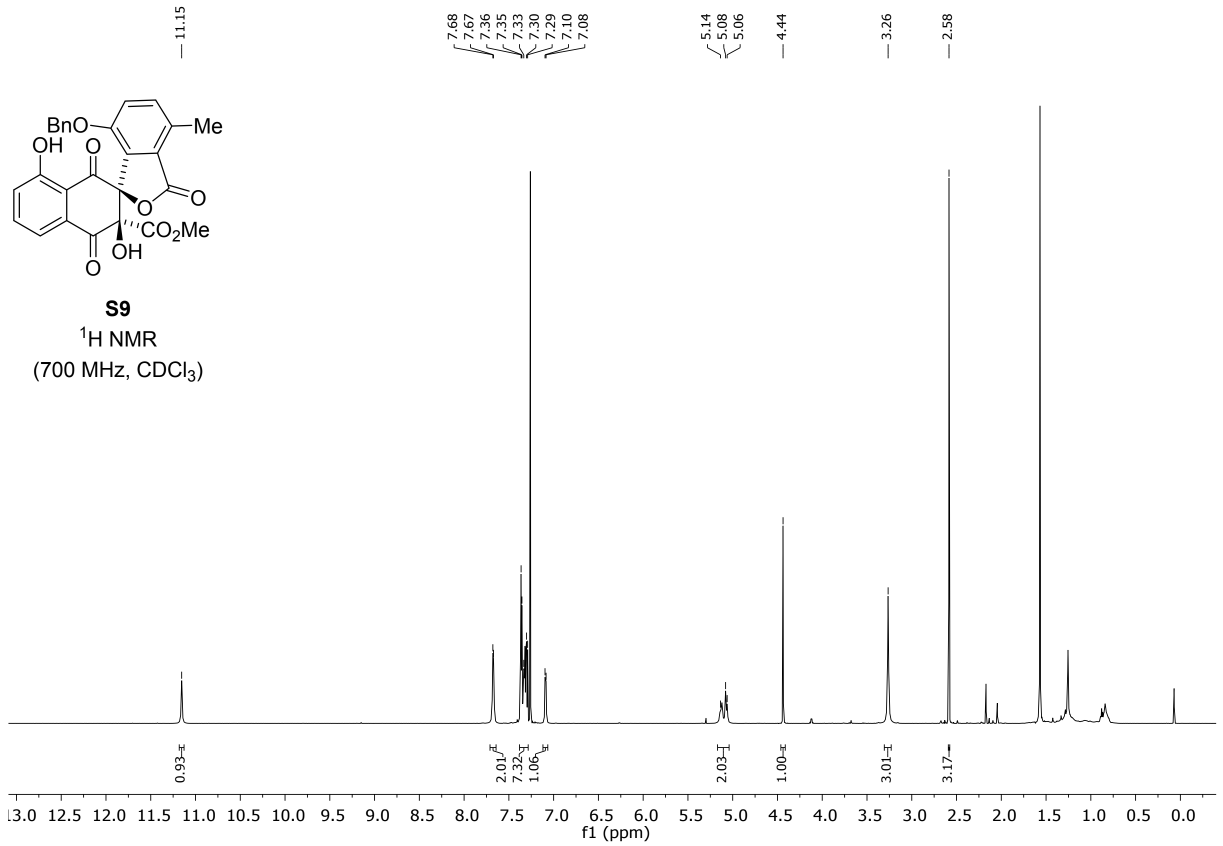

S9
^{1}H NMR
(700 MHz, $CDCl_3$)

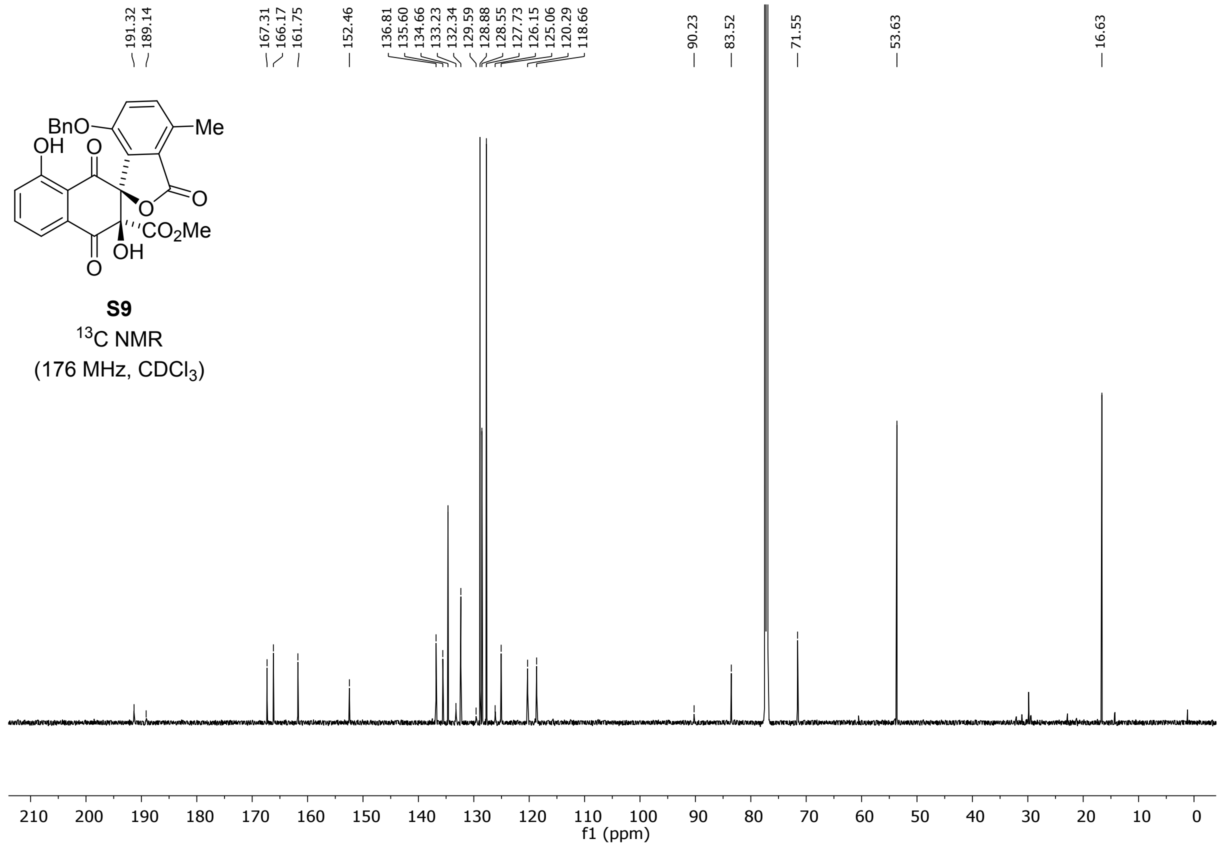

S9
^{13}C NMR
(176 MHz, $CDCl_3$)

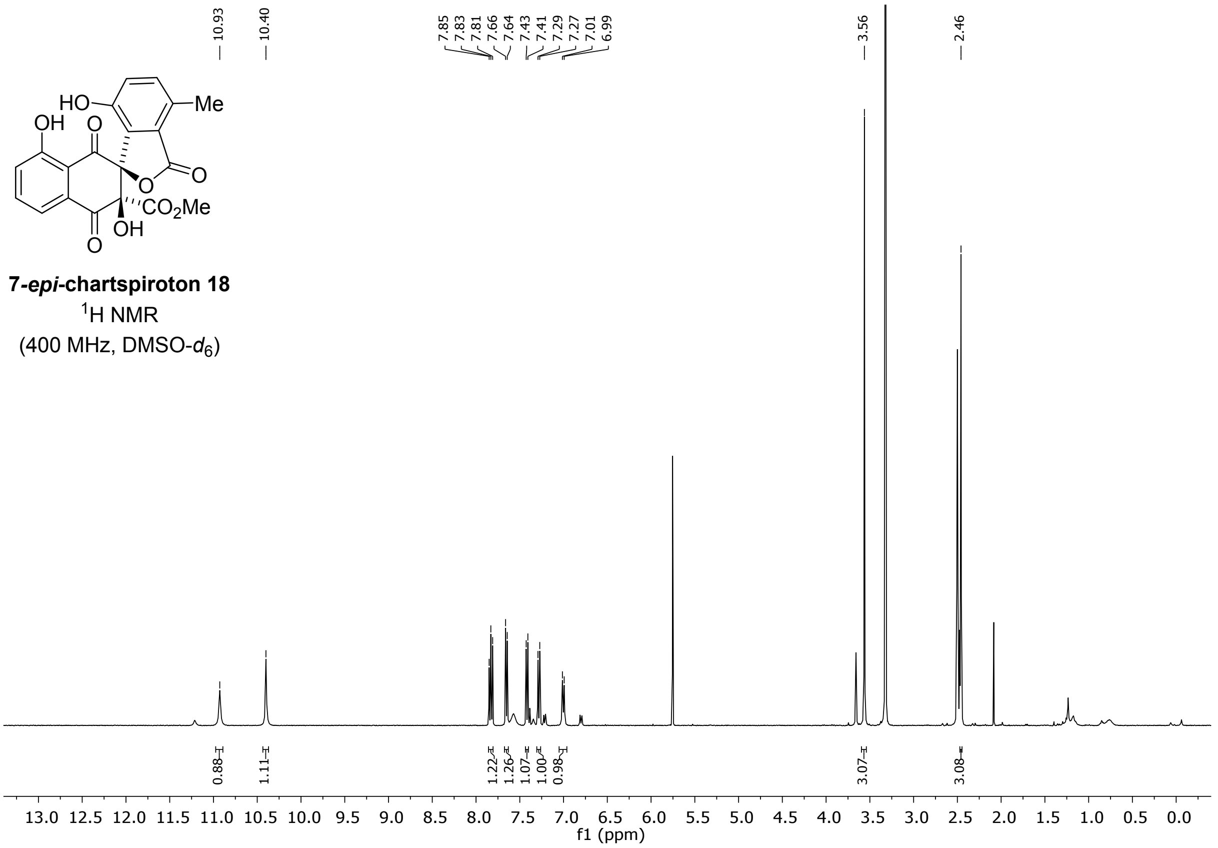
7-epi-chartspiroton 18
^{1}H NMR
(400 MHz, DMSO-d_6)
f1 (ppm)

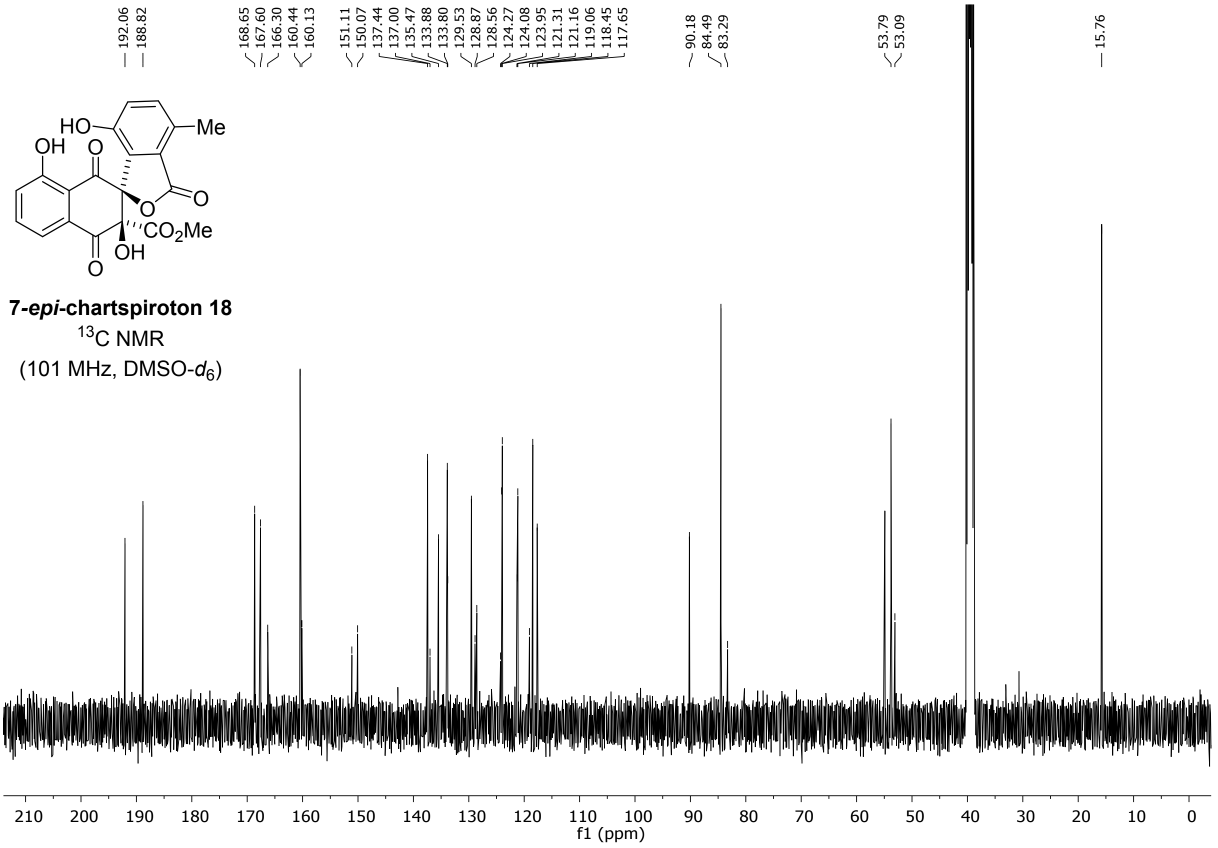
7-epi-chartspiroton 18
^{13}C NMR
(101 MHz, DMSO-d_6)
f1 (ppm)

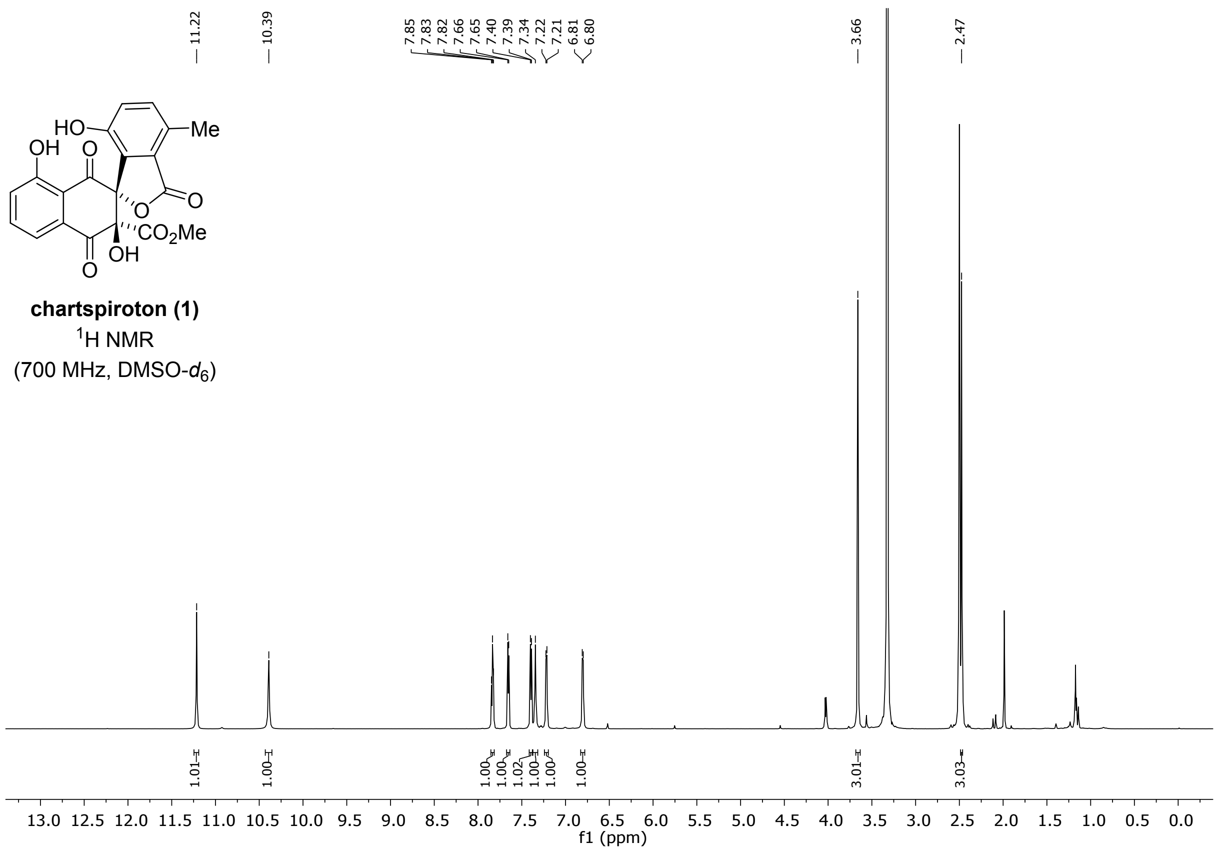
11.22
10.39
7.85
7.83
7.82
7.66
7.65
7.40
7.39
7.34
7.22
7.21
6.81
6.80
3.66
2.47
HO
Me
OH
O
O
O
CO2Me
OH
O
chartspiroton (1)
1H NMR
(700 MHz, DMSO-d6)
1.01
1.00
1.00
1.00
1.02
1.00
1.00
1.00
3.01
3.03
13.0 12.5 12.0 11.5 11.0 10.5 10.0 9.5 9.0 8.5 8.0 7.5 7.0 6.5 6.0 5.5 5.0 4.5 4.0 3.5 3.0 2.5 2.0 1.5 1.0 0.5 0.0
f1 (ppm)

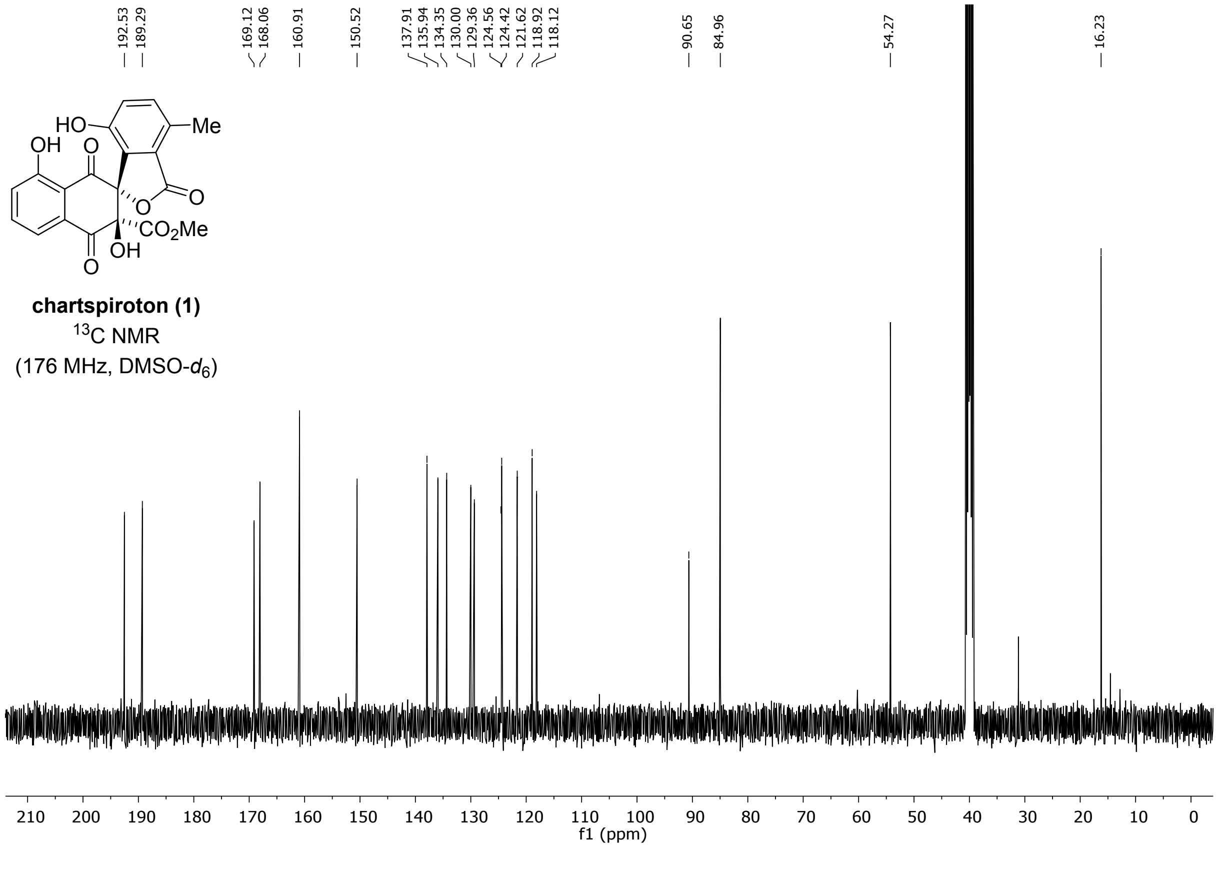
192.53
189.29
169.12
168.06
160.91
150.52
137.91
135.94
134.35
130.00
129.36
124.56
124.42
121.62
118.92
118.12
90.65
84.96
54.27
16.23
HO
Me
OH
O
O
O
CO2Me
OH
O
chartspiroton (1)
13C NMR
(176 MHz, DMSO-d6)
210 200 190 180 170 160 150 140 130 120 110 100 90 80 70 60 50 40 30 20 10 0
f1 (ppm)

3. Crystallographic Data

3-(Allyloxy)-2-(6-(benzyloxy)-2-bromo-3-methylphenyl)-7-methoxy-1*H*-inden-1-one 10b

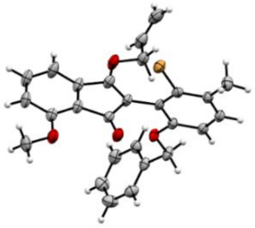

CCDC 2334636 contains the supplementary crystallographic data for **10b**.

Identification code	mar23_8	
Empirical formula	$C_{27}H_{23}BrO_4$	
Formula weight	491.36	
Temperature	173.00 K	
Wavelength	0.71073 Å	
Crystal system	Monoclinic	
Space group	$P2_1/c$ (no. 14)	
Unit cell dimensions	a = 11.2281(12) Å	α= 90°
	b = 21.412(2) Å	β= 102.274(3)°
	c = 9.8802(10) Å	γ = 90°
Volume	2321.1(4) $Å^3$	
Z	4	
Density (calculated)	1.406 Mg/m^3	
Absorption coefficient	1.802 mm^{-1}	
F(000)	1008	
Crystal size	0.18 x 0.1 x 0.015 mm^3	
Theta range for data collection	2.314 to 25.400°	
Index ranges	−13<=h<=13, −25<=k<=25, −10<=l<=11	
Reflections collected	19407	
Independent reflections	4245 [R(int) = 0.0630]	
Completeness to theta = 25.242°	99.7 %	
Absorption correction	Semi-empirical from equivalents	
Max. and min. transmission	0.9420 and 0.6287	
Refinement method	Full-matrix least-squares on F^2	
Data / restraints / parameters	4245 / 0 / 291	
Goodness-of-fit on F^2	1.060	
Final R indices [I>2sigma(I)]	R1 = 0.0431, wR2 = 0.0994	
R indices (all data)	R1 = 0.0639, wR2 = 0.1091	
Extinction coefficient	n/a	
Largest diff. peak and hole	0.431 and −1.008 e.$Å^{-3}$	

Methyl 3-(6-(benzyloxy)-2-bromo-3-methylphenyl)-5-methoxy-1,4-dioxo-1,4-dihydronaphthalene -2-carboxylate 5b

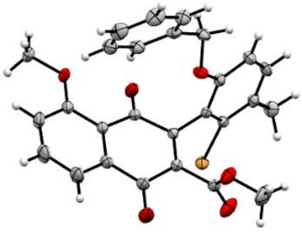

CCDC 2334635 contains the supplementary crystallographic data for **5b**.

Identification code	mar24_2	
Empirical formula	$C_{27}H_{21}BrO_6$	
Formula weight	521.35	
Temperature	173.00 K	
Wavelength	0.71073 Å	
Crystal system	Triclinic	
Space group	P-1 (no. 2)	
Unit cell dimensions	a = 9.8342(4) Å	α= 75.441(2)°
	b = 10.9798(5) Å	β= 83.455(2)°
	c = 11.1034(5) Å	γ = 84.754(2)°
Volume	1150.40(9) $Å^3$	
Z	2	
Density (calculated)	1.505 Mg/m^3	
Absorption coefficient	1.829 mm^{-1}	
F(000)	532	
Crystal size	0.15 x 0.11 x 0.08 mm^3	
Theta range for data collection	2.352 to 27.190°	
Index ranges	−12<=h<=12, −14<=k<=14, −14<=l<=14	
Reflections collected	68423	
Independent reflections	5100 [R(int) = 0.0341]	
Completeness to theta = 25.242°	99.8 %	
Absorption correction	Semi-empirical from equivalents	
Max. and min. transmission	0.8372 and 0.7859	
Refinement method	Full-matrix least-squares on F^2	
Data / restraints / parameters	5100 / 0 / 310	
Goodness-of-fit on F^2	1.062	
Final R indices [I>2sigma(I)]	R1 = 0.0219, wR2 = 0.0577	
R indices (all data)	R1 = 0.0231, wR2 = 0.0586	
Extinction coefficient	n/a	
Largest diff. peak and hole	0.383 and −0.229 e.$Å^{-3}$	

Methyl (1*S*,3'*R*)-7-(benzyloxy)-3'-hydroxy-8'-methoxy-4-methyl-1',3,4'-trioxo-3',4'-dihydro-1'*H*,3*H*-spiro[isobenzofuran-1,2'-naphthalene]-3'-carboxylate 17a

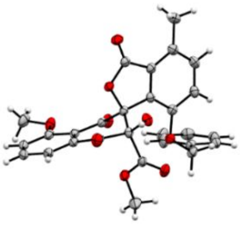

CCDC 2334637 contains the supplementary crystallographic data for **17a**.

Identification code	mar23_7	
Empirical formula	$C_{28}H_{22}O_9$	
Formula weight	502.45	
Temperature	173.00 K	
Wavelength	0.71073 Å	
Crystal system	Monoclinic	
Space group	$P2_1/c$ (no. 14)	
Unit cell dimensions	a = 11.9390(4) Å	α= 90°
	b = 17.8674(6) Å	β= 99.0760(10)°
	c = 10.9374(3) Å	γ = 90°
Volume	2303.94(13) $Å^3$	
Z	4	
Density (calculated)	1.449 Mg/m^3	
Absorption coefficient	0.109 mm^{-1}	
F(000)	1048	
Crystal size	0.18 x 0.1 x 0.08 mm^3	
Theta range for data collection	2.070 to 26.778°	
Index ranges	−15<=h<=14, −22<=k<=22, −13<=l<=12	
Reflections collected	40285	
Independent reflections	4910 [R(int) = 0.0366]	
Completeness to theta = 25.242°	99.9 %	
Absorption correction	Semi-empirical from equivalents	
Max. and min. transmission	0.9586 and 0.9371	
Refinement method	Full-matrix least-squares on F^2	
Data / restraints / parameters	4910 / 1 / 342	
Goodness-of-fit on F^2	1.045	
Final R indices [I>2sigma(I)]	R1 = 0.0347, wR2 = 0.0894	
R indices (all data)	R1 = 0.0416, wR2 = 0.0956	
Extinction coefficient	0.0037(8)	
Largest diff. peak and hole	0.304 and −0.236 e.$Å^{-3}$	
